# The Stepping Threshold Test for Reactive Balance: Validation of Two Observer-Based Evaluation Strategies to Assess Stepping Behavior in Fall-Prone Older Adults

**DOI:** 10.3389/fspor.2021.715392

**Published:** 2021-10-11

**Authors:** Michael Adams, Leon Brüll, Monika Lohkamp, Michael Schwenk

**Affiliations:** ^1^Network Aging Research, Heidelberg University, Heidelberg, Germany; ^2^School for Therapeutic Sciences, SRH University Heidelberg, Heidelberg, Germany; ^3^Department of Training and Movement Sciences, Humboldt-Universität zu Berlin, Berlin, Germany; ^4^Berlin School of Movement Science, Humboldt-Universität zu Berlin, Berlin, Germany; ^5^Institute of Sports and Sports Sciences, Heidelberg University, Heidelberg, Germany; ^6^Department of Sport Science, Human Performance Research Center, University of Konstanz, Konstanz, Germany

**Keywords:** reactive balance, assessment, step threshold, perturbation, validity, fall prevention, fallers

## Abstract

**Introduction:** Measurement of reactive balance is critical for fall prevention but is severely underrepresented in the clinical setting due to the lack of valid assessments. The Stepping Threshold Test (STT) is a newly developed instrumented test for reactive balance on a movable platform, however, it has not yet been validated for fall-prone older adults. Furthermore, different schemes of observer-based evaluation seem possible. The aim of this study was to investigate validity with respect to fall risk, interpretability, and feasibility of the STT using two different evaluation strategies.

**Methods:** This study involved 71 fall-prone older adults (aged ≥ 65) who underwent progressively increasing perturbations in four directions for the STT. Single and multiple-step thresholds for each perturbation direction were determined *via* two observer-based evaluation schemes, which are the 1) consideration of all steps (all-step-count evaluation, ACE) and 2) consideration of those steps that extend the base of support in the direction of perturbation (direction-sensitive evaluation, DSE). Established balance measures including global (Brief Balance Evaluations Systems Test, BriefBEST), proactive (Timed Up and Go, TUG), and static balance (8-level balance scale, 8LBS), as well as fear of falling (Short Falls Efficacy Scale—International, FES-I) and fall occurrence in the past year, served as reference measurements.

**Results:** The sum scores of STT correlated moderately with the BriefBEST (ACE: *r* = 0.413; DSE: *r* = 0.388) and TUG (ACE: *r* = −0.379; DSE: *r* = −0.435) and low with the 8LBS (ACE: *r* = 0.173; DSE: *r* = 0.246) and Short FES-I (ACE: *r* = −0.108; DSE: *r* = −0.104). The sum scores did not distinguish between fallers and non-fallers. No floor/ceiling effects occurred for the STT sum score, but these effects occurred for specific STT thresholds for both ACE (mean floor effect = 13.04%, *SD* = 19.35%; mean ceiling effect = 4.29%, *SD* = 7.75%) and DSE (mean floor effect = 7.86%, *SD* = 15.23%; mean ceiling effect = 21.07%, *SD* = 26.08). No severe adverse events occurred.

**Discussion:** Correlations between the STT and other balance tests were in the expected magnitude, indicating convergent validity. However, the STT could not distinguish between fallers and non-fallers, referring to a need for further studies and prospective surveys of falls to validate the STT. Current results did not allow a definitive judgment on the advantage of using ACE or DSE. Study results represented a step toward a reactive balance assessment application in a clinical setting.

## Introduction

Approximately every third person aged 65 and above experiences at least one fall annually (World Health Organization, 2007). Early detection of individuals at high risk for falls could help prevent falls and reduce health care costs. The most commonly used measurements to detect impairments in postural control are measures of static and dynamic balance (Sibley et al., 2011), such as the single-leg stance, the Berg Balance Scale (Berg, 1989), and the Timed Up and Go (TUG) Test (Podsiadlo and Richardson, 1991). However, neither these nor other fall risk assessments demonstrate sufficient ability to distinguish between older adults at high and low risk for falls (Balasubramanian et al., 2015; Lusardi et al., 2017; Park, 2018).

Reactive balance control, which is the ability to recover from an unexpected loss of balance, is a critical component of postural control for fall prevention (McIlroy and Maki, 1996). At the same time, reactive balance is the least assessed component of postural control in the clinical setting (Sibley et al., 2011). In a cross-sectional survey by Sibley et al. (2013), nearly 80% of the clinicians, who reported to assess reactive balance, used only non-standardized observation-based methods of assessing reactive control. Even clinicians who rely on standardized tools must cope with severe limitations. Validated tools, such as the Balance Evaluation Systems Test (BEST) (Horak et al., 2009) and the Tinetti Balance and Gait Test (Tinetti, 1986), that include reactive balance items, have limited accuracy due to few items and a coarse scale. These tests do not reproduce the unpredictability of unexpected loss of balance, which is an important requirement for testing reactive control (Maki and McIlroy, 2006) but is difficult to ensure in a standardized test. Accordingly, there is a concerning lack of clinical approaches for measuring reactive balance ability.

In the scientific setting, several approaches have been developed, e.g., perturbations by cable pull (Hilliard et al., 2008), sudden cable release of tethered lean (Carty et al., 2015), and platform motions (Maki and McIlroy, 2006; Madigan et al., 2018; Aviles et al., 2019). Emerging technologies enable the computerized application of perturbations in various directions, intensities, time intervals, and under controlled, safe conditions (Shapiro and Melzer, 2010). This provides the opportunity to simulate the unpredictability of events that lead to loss of balance in daily life.

In previous studies, reactive single-step and multi-step responses (respectively lower step thresholds) have been shown to be independent predictors of future falls in community-dwelling older adults (Hilliard et al., 2008; Batcir et al., 2020; Crenshaw et al., 2020). A recent meta-analysis of 12 studies came to the results that reactive stepping tests can distinguish moderately between fallers and non-fallers (Okubo et al., 2021), but the studies differ greatly in their applied methods and results. A unified and standardized measurement procedure of reactive control in healthy older adults that is both valid and feasible for clinical uptake is still missing.

In this context, the study of Handelzalts et al. (2019a,b) presented a promising test approach. They applied perturbations by platform translations in four directions and at six progressive intensity levels to assess reactive balance ability in healthy adults and individuals after stroke. The single-step and multiple stepping thresholds were determined. The assessment tool developed, hereafter referred to as the Stepping Threshold Test (STT), proved to be inter-observer reliable in both populations and convergent validity for individuals after stroke (Handelzalts et al., 2019b). However, data on the validity of the STT in healthy older adults are not yet available.

In previous studies, each step after a perturbation was counted to determine the number of steps required to regain balance (Mille et al., 2013; Crenshaw et al., 2020) or the step and stepping thresholds (Batcir et al., 2018, 2020; Handelzalts et al., 2019a,b). The study of Handelzalts et al. (2019a) defined steps on the basis of an extension of the base of support (BoS). The study of Arampatzis et al. (2008) also considered the direction of perturbation in their definition. They used a cable release system and defined a multiple stepping as any second step taken by the recovery limb or an anterior exceeding of the first step by the contralateral limb (Arampatzis et al., 2008).

From a biomechanical view, a consideration of the extension of the BoS and the direction of perturbation could lead to a further refinement of the step evaluation strategy of the STT. Perturbations lead to a movement of the center of mass (CoM) (Maki and McIlroy, 1997). Step and stepping strategies aim to modify the BoS in order to maintain the CoM within the stability limits of the BoS (Maki and McIlroy, 1997). Thus, if a step extends the BoS in a different direction than the CoM movement, it cannot directly support rebalancing and cannot be considered as part of an efficient reactive strategy. Accordingly, an efficient step and stepping strategy at the step threshold extends the BoS toward CoM motion and therefore opposite to the direction of surface translation. Other strategies could reflect an inadequate reaction or might merely serve to increase standing comfort. When considering multiple steps, it should be taken into account that the BoS has already changed after the first step. Consequently, every single step that follows the first step should be evaluated based on the actual (newly formed) BoS.

For this reason, we developed a new strategy to evaluate the step and stepping behavior of the STT, which we called the ‘direction-sensitive evaluation' (DSE). As opposed to counting every step is taken (Handelzalts et al., 2019b; Batcir et al., 2020; Crenshaw et al., 2020), which we called the ‘all-step-count evaluation' (ACE), our approach considers two important characteristics in the step and stepping behavior. First, our approach leads to a direction-specific consideration since steps counted only in the opposite direction to the surface translation. Second, single steps and multiple steps are counted only if they extended the actual BoS.

This investigation had three aims. Our first aim was to test the convergent validity of the STT in fall-prone older adults with respect to fall risk. For this purpose, we used an established method and investigated associations between widely used clinical measures of balance and fall risk (Handelzalts et al., 2019b) and the STT sum score (convergent validity). We expected to find moderate correlations with the Brief Balance Evaluations Systems Test (BriefBEST, global balance), moderate correlation with the TUG (proactive balance), low to moderate correlations with the 8-level balance scale (8LBS, static balance), and low to moderate correlations with the Short Falls Efficacy Scale—International (Short FES-I, fear of falling). This expectation is based on the results of Handelzalts et al. (2019b), Crenshaw et al. (2018), and a meta-analysis by Kiss et al. (2018) who found associations between reactive balance and other balance domains. Our second aim was to explore the association between the STT and the experience of at least one fall in the past 12 months. We hypothesized to find significant differences in the STT sum score between fallers and non-fallers in the past year (discriminative validity). Past falls are among the strongest risk factors for future falls (Ek et al., 2019) and fallers use significantly more recovery steps after perturbations than non-fallers (Okubo et al., 2021). Our third aim was to evaluate the feasibility and interpretability of the STT. We hypothesized the test to be safe and feasible in fall-prone older adults. The study of Handelzalts et al. (2019b) successfully applied the STT in the vulnerable group of individuals with stroke. Our fourth aim was to compare the validity of the ACE and DSE in order to explore the advantages of a differentiated step evaluation and to advance the standardization of the measurement process. We hypothesized to find stronger evidence for convergent and discriminative validity in the DSE compared with the ACE since the DSE leads to a more differentiated consideration of stepping behavior.

## Methods

### Study Participants

**Figure 1:**
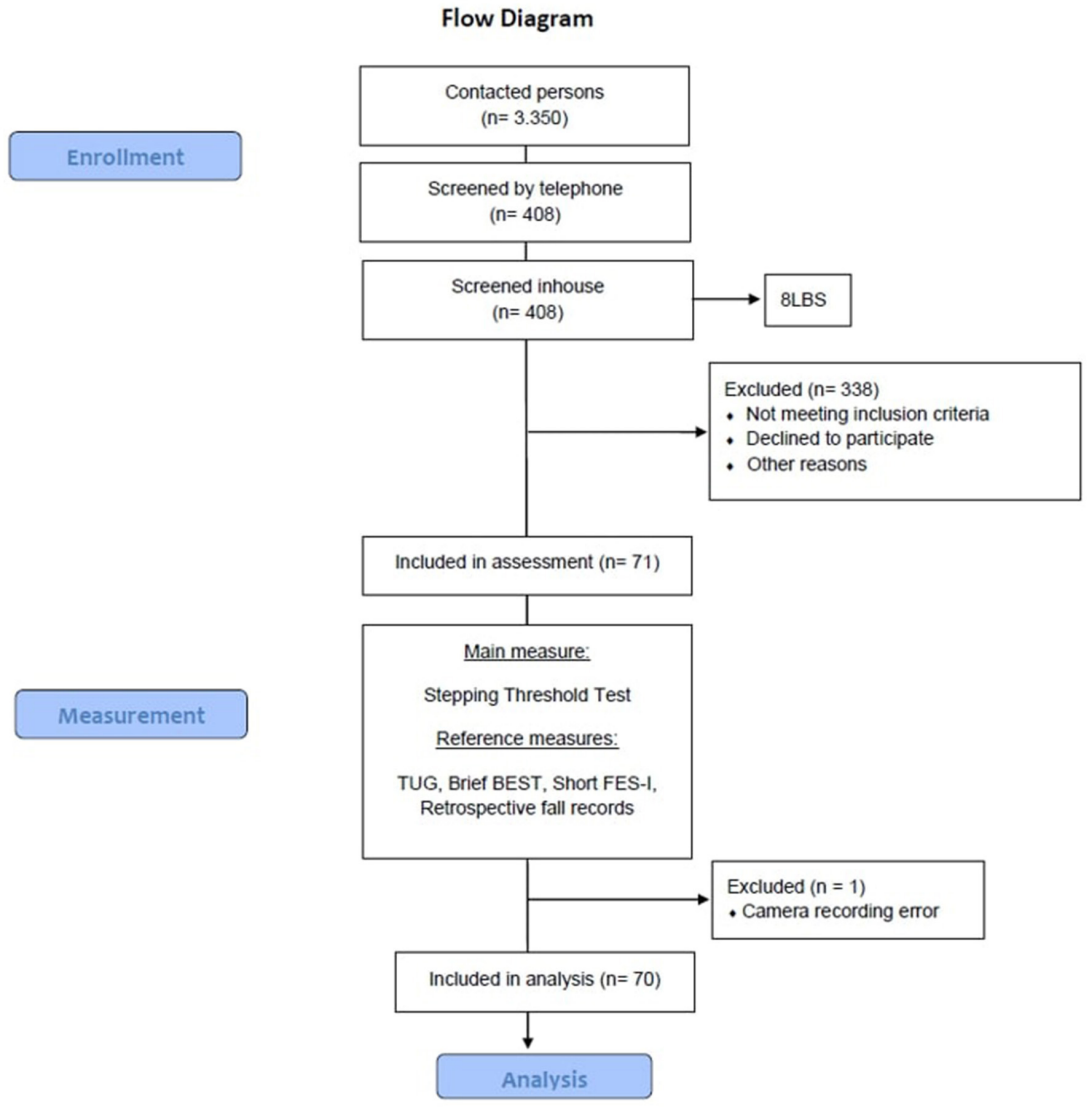
The course of study. BriefBEST, Brief Balance Evaluations Systems Test; FES-I, Short Falls Efficacy Scale—International; TUG, Timed Up and Go Test; 8LBS, Eight level balance scale.

This methodological study used baseline data of an intervention study on perturbation-based balance training registered at clinicaltrials.gov (trial register number: NCT04087512). A sample of 71 community-dwelling adults aged 65 and older was recruited. We contacted 3,350 people *via* a random selection by the local resident registration office. Eligibility criteria were assessed in a two-step procedure consisting of a standardized telephone screening and a face-to-face screening (Figure 1). Eligible subjects were invited to the baseline assessment. Subjects had to be able to walk for at least 20 min without a walking aid and had to be fall-prone. The latter could be met in two ways. It was identified either the subject has experienced a fall in the last 12 months or a subjective feeling of a decrease in balance ability in the past year and a deficit in balance ability, defined as a loss of balance ability on the 8LBS (Clemson et al., 2012; Weber et al., 2018) to level 4 (tandem standing with eyes closed). Exclusion criteria included severe metabolic, cardiovascular, pulmonary, neurological, or orthopedic diseases. Moreover, subjects were excluded if cognitive impairment was suspected due to a score below eight on DemTect (Kessler et al., 2000). Other reasons for exclusion were strong dizziness, a body mass index above 30, significant visual or sensory impairments, and participation in balance training in the last 3 months. This study was carried out in accordance with the Declaration of Helsinki and approved by the ethics committee of Heidelberg University (reference: AZ Schwe 2019 /1-2).

### Measurements

Demographic characteristics and falls within the last 12 months (retrospective) were assessed and recorded during standardized interviews. For this purpose, a fall was operationally defined as an unexpected event in which a person walking, standing, sitting, or lying down involuntarily, suddenly, and uncontrollably comes to rest on the ground or another lower level (Hauer et al., 2006). Participants were classified as non-fallers and fallers (at least one fall in the past 12 months) (Crenshaw et al., 2020).

**Figure 2:**
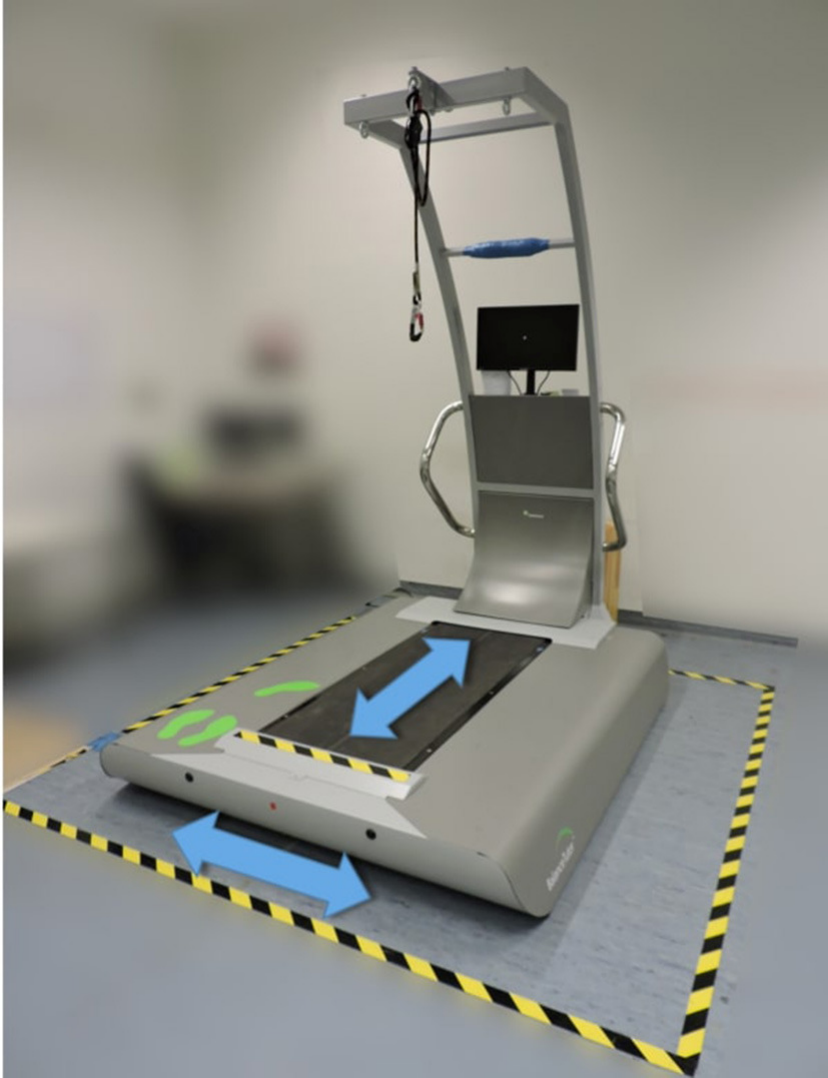
Balance tutor and directions of surface translation.

For the testing procedure of the STT, we used a commercial perturbation treadmill (Balance Tutor, MediTouch, Israel) (Figure 2). The study of Shapiro and Melzer (2010) described the system configuration. Starting from approaches of previous studies (Batcir et al., 2018, 2020; Handelzalts et al., 2019a,b) that use step and stepping thresholds to estimate reactive balance, we defined the STT as follows: Participants were instructed to stand on the Balance Tutor in their shoes with their both feet together and to respond to unannounced surface translation perturbations (backward, forward, left, and right) with as few compensatory steps as possible. The test was composed of six levels with increasing intensity (Table 1). Each level contained four unannounced surface translations, one in each direction. An additional perturbation that was not included in the analysis was added to the sequence (in level 4 of 6) to ensure the unpredictability of the perturbation direction. The order of directions varied randomly between the levels (Supplementary Material 1). The order of perturbation intensity was not randomized but gradually increasing because we aimed to determine participants' single-step and multiple stepping thresholds. Participants were exposed to each perturbation only once. The perturbations lasted 0.5 s and the intervals in between were 10 and 19.5 s (Supplementary Material 1). Familiarization with the perturbation treadmill consisted of 10–20 min of normal walking on the treadmill at the face-to-face screening (12.3 ± 4.7 days before the actual assessment), a full body weight relief into the harness system, and two perturbations at the lowest intensity level prior to the test. Subsequently, the STT was performed.

**Table 1:** Characteristics of surface translations.

**Level of the STT**	**Displacement anteroposterior (cm)** ^**a**^	**Displacement mediolateral (cm)** ^**b**^
1	7.4	3.3
2	12.9	6.3
3	18.5	9.2
4	23.9	12.1
5	29.5	15.1
6	35.0	18.0

a *Displacement of treadmill surface in the forward and backward direction*.

b *Displacement of treadmill surface in left and right direction*.

**Figure 3:**
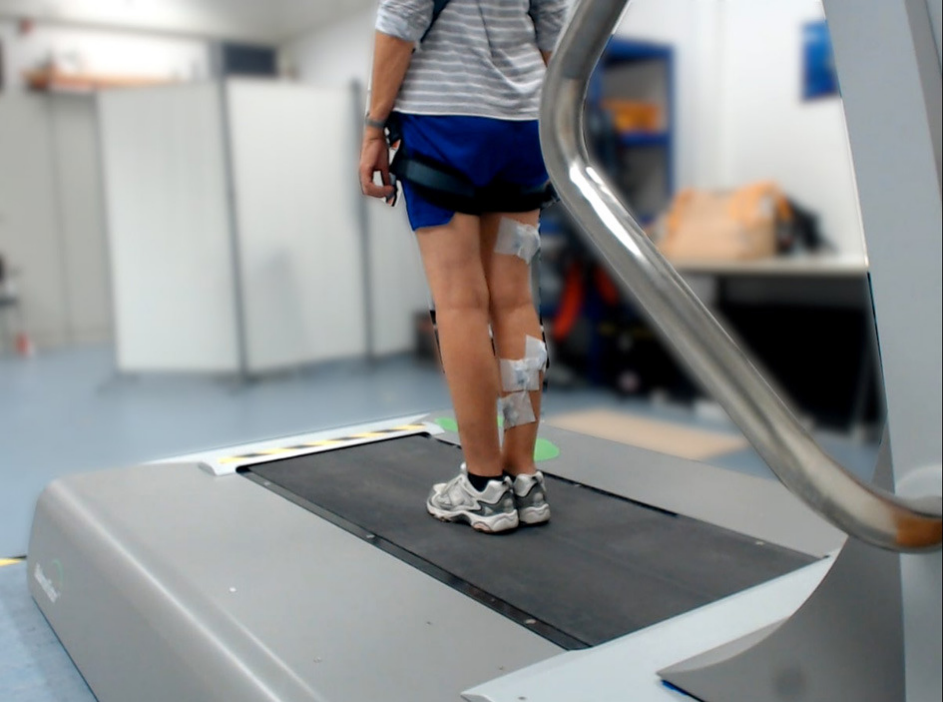
The perspective of the camera.

The stepping behavior of the participants was evaluated for all 24 surface translations. In order to avoid injuries, the participants wore a safety harness that protected them from falling. The rope length was adjusted so that in the event of a fall, the knees of the participants would come to rest ~10 cm above the treadmill surface. In case of a fall or excessive fear by the participant, the test was terminated prematurely. The testing process was recorded on video from the thoracic spine of the participant downwards. The camera system (Logitech C920HD Pro Webcam, Logitech, Apples, Switzerland) was placed at a distance of 2.1 m and an angle of 35° dorsolateral to the participant (Figure 3) and recorded at frame rates of 30 Hz.

The evaluation of the stepping behavior of the participants was assessed by video analysis. Stepping behavior was scored as no step, single step, or multiple stepping. For the ACE, we counted each step up to the point where the subject regained balance, based on the observational judgment of a static steady-state balance, i.e., maintaining a steady position while standing with a stable trunk. For this purpose, we defined a step as an observable change in the bipedal BoS. In the DSE, we specified a step as reaction behavior that leads to a sensible extension of the BoS in the opposite direction of the surface translation. To be counted as a single step, the BoS in the basic test position had to be extended by one step in the opposite direction to the surface translation. To be counted as a multiple-step, the actual BoS had to be additionally extended by one or more steps in the opposite direction to the surface translation. Subsequently, single-step and multiple stepping thresholds were determined for each direction of surface translation (forward, backward, left, and right). In the increasing perturbation protocol, the single-step threshold was defined as the first perturbation displacement from which the subject needed to take a step to recover. The multiple stepping threshold was defined as the first perturbation displacement from which the subject needed to take multiple steps, i.e., at least two steps, to recover. To ensure that the threshold was reached, two successive perturbations in the same direction each had to result in a single step or multiple stepping for the threshold to be scored (Batcir et al., 2018). The first of these two consecutive perturbations was set as the threshold. Some participants did not reach all of the eight step and stepping thresholds (original thresholds). In this case, the threshold value was set at one level above the highest executed level as conducted before (Handelzalts et al., 2019a). The thresholds were termed according to the direction of the surface translation (e.g., single-step threshold forward).

We used several established and widely used clinical assessments for balance and fall risk as reference measures. The Brief Balance Evaluations Systems Test was obtained by an assessor as described elsewhere (Marques et al., 2016). It is a shortened version of Horak's BESTest (Padgett et al., 2012) and consists of six items, measuring aspects of static, dynamic, proactive, and reactive postural control in standing and walking. The Timed Up and Go Test is a widely used performance-based assessment of dynamic balance and fall risk (Podsiadlo and Richardson, 1991). It was assessed per protocol, by measuring the time needed by the participant to stand up from a chair, walk three meters at a brisk but safe pace, turn 180 degrees, and walk back to the chair to sit down. The 8-level balance scale is a further development of the Short Physical Performance Battery (Guralnik et al., 1994). It comprises eight static balance tasks with increasing difficulty. Every task needed to be performed for 15 s without external support, the use of a reactive step, or compensatory arm movements (Clemson et al., 2012; Gordt et al., 2020). Fear of falling was assessed by the interviewer using the Short FES-I (Kempen et al., 2008). Participants rated their level of confidence during seven activities of daily life on a 4-point Likert scale, with a lower value representing more confidence.

### Statistical Analysis

Statistical analyses were performed using IBM SPSS Statistics Version 26 (IBM, New York, NY, USA) and MS Excel 2010 (Microsoft, Redmond, Washington, USA). Hypotheses were two-sided evaluated at the alpha level at *p* < 0.05. The primary outcome was the STT sum score, calculated as the sum of all eight original single-step and multiple stepping thresholds. Secondary outcomes included the STT subscores, i.e., sums of single-step thresholds, multiple stepping thresholds, mediolateral (left and right) step and stepping thresholds, anteroposterior (forward and backward) step and stepping thresholds, and the original thresholds for each (forward, backward, left, and right) single-step and multiple stepping threshold.

Descriptive statistics were used to characterize the study population. Differences between non-fallers and fallers with regards to demographics, postural balance capacity, and fear of falling were analyzed by means of Chi^2^-Test for categorical variables, and by either the Mann-Whitney-U test or the independent *t*-test, as indicated, for continuous variables. Normal distribution was tested by means of the Shapiro-Wilk W test. For the estimation of convergent validity, we investigated the association of the STT with the TUG, BriefBEST, 8LBS, and the Short-FES-I applying the Spearman's rank correlation coefficient. Correlation coefficients of *r* = 0.1–0.29 indicate a small, *r* = 0.3–0.49 moderate and *r* ≥ 0.50 strong correlations (Cohen, 1988). Discriminative validity was calculated by the Mann-Whitney U test and non-parametric receiver operating characteristic (ROC) curve analysis. The Mann-Whitney U statistics were applied to determine differences between the groups of fallers with respect to the STT. The receiver operating characteristic curve analysis was used to determine the prognostic value in order to evaluate a difference between fallers and non-fallers, by means of the area under the curve (AUC). As the non-parametric ROC analysis is based on the Mann-Whitney U statistic, we reported only the ROC curves of the STT variables that were significantly different between different groups of fallers. The area under the curve values of the ROC were classified into non-informative (AUC = 0.5), less accurate (0.5 < AUC ≤ 0.7), moderately accurate (0.7 < AUC ≤ 0.9), very accurate (0.9 < AUC <1), and perfect (AUC = 1) (Greiner et al., 2000). Information about feasibility was examined based on the rate of early test terminations and the occurrence of adverse events during the STT. Adverse events were defined as any unfavorable or unintended event that occurs in the course of this study (Ory et al., 2005). Floor and ceiling effects occur when a distinct percentage of subjects achieve the worst or best possible score and reflect an incomplete distribution of sample within a test and insufficiency to distinguish subjects at the lower and upper ends of the measurement system (McHorney and Tarlov, 1995). They were defined to be present if more than 15% of subjects reached the highest and lowest level, respectively (McHorney and Tarlov, 1995). A sensitivity analysis using G*Power 3.1.9.7 (Faul et al., 2009) showed that with a sample size of *n* = 70 a correlation of 0.327 can be shown with power 0.8 using a significance level of 0.05.

## Results

### Demographics

A consecutively recruited sample of 70 fall-prone older adults with a mean age of 74.8 years (*SD* = 6) was included in the analysis (Table 2). From the 71 recruited participants, one had to be excluded from analysis due to technical problems and incomplete data. Among the included participants, 32 (46.5%) had experienced at least one fall in the past 12 months and were therefore classified as fallers. There were significantly more women categorized as fallers than as non-fallers (*p* = 0.007). No further significant differences were found in regards to age, gender, balance capacity, and fear of falling between non-fallers and fallers (Table 2).

**Table 2:** Study population characteristics.

	**All participants (*n* = 70)**	**Non-fallers(*n* = 38)**	**Fallers** **(*n* = 32)**	**Sign**.
N (%) Women	45 (64.3)	19 (50.0)	26 (81.3)	0.007
Mean Age ± SD	74.8 ± 6.0	75.4 ± 6.4	74.0 ± 5.3	0.385
Median BriefBEST (IQR)	18 (4.25)	17.5 (5)	18 (3.75)	0.374
Mean TUG ± SD	7.8 ± 1.3	8.1 ± 1.4	7.5 ± 1.0	0.600
Median 8LBS (IQR)	5 (1)	5 (2)	5 (1)	0.104
Median Short FES-I (IQR)	8 (2.25)	8 (3)	8.5 (2)	0.370

*Sign., One-tailed significance level was set to p < 0.05. BriefBEST, Brief Balance Evaluations Systems Test; FES-I, Short Falls Efficacy Scale—International. IQR, Interquartile range; TUG, Timed Up and Go Test; 8LBS, Eight level balance scale; P-value calculated by means of the Mann-Whitney-U test*.

### Convergent Validity

The Stepping Threshold Test sum score (ACE) correlated moderately with the BriefBEST (*r* = 0.413) and the TUG (*r* = −0.379). In addition, the STT sum score (ACE) correlated low with the 8LBS (*r* = 0.173) and the Short FES-I (*r* = 0.108) (Table 3). The Stepping Threshold Test subscores (ACE) correlated low (*r* = 0.102 to |−0.297|) in 8 of 16 values, moderately in 6 values (*r* = 0.312 to |−0.433|), and did not correlate with the reference measures in 2 values (Table 3). The single-step thresholds (ACE) correlated in low 8 of 16 values (*r* = 0.107 to |−0.293|), moderately in 5 values (*r* = 0.300 to |−0.390|), and did not correlate in 3 values with the reference measures (Supplementary Material 2.1). The multiple stepping thresholds (ACE) correlated low in 12 of 16 values (*r* = 0.104 to |−0.292|), moderately in 1 value (*r* = 0.309), and did not show correlations with the reference measures in 3 values (Supplementary Material 2.1). Correlation plots for visual inspection are presented in the appendices (Supplementary Material 3.1).

**Table 3:** Correlation between STT sum score and subscores (ACE) and reference measures.

	**STT-Thresholds**		**BriefBEST**	**TUG**	**8LBS**	**Short FES-I**
**Primary outcome**	STT sum score	r	0.413	−0.379	0.173	−0.108
		CI95	0.19–0.6	−0.57–0.15	−0.07–0.39	−0.34–0.13
**Secondary outcome**	SS subscore	r	0.425	−0.433	0.144	−0.021
		CI95	0.2–0.61	−0.61–0.21	−0.1–0.37	−0.25–0.22
	MS subscore	r	0.318	−0.280	0.135	−0.120
		CI95	0.08–0.52	−0.49–0.04	−0.1–0.36	−0.35–0.12
	AP subscore	r	0.372	−0.297	0.161	−0.195
		CI95	0.14–0.56	−0.5–0.06	−0.08–0.38	−0.41–0.04
	ML subscore	r	0.312	−0.314	0.102	−0.008
		CI95	0.08–0.51	−0.52–0.08	−0.14–0.33	−0.24–0.23

*Sign., Two-tailed significance; SS, Single-step; MS, Multiple Stepping; AP, Anteroposterior; ML, Mediolateral; CI95, confidence interval of 95%; r, correlation coefficient rho, calculated by means of the Spearman-rank-correlation; TUG, Timed Up and Go Test; BriefBEST, Brief Balance Evaluations Systems Test; 8LBS, Eight level balance scale; FES-I, Short Falls Efficacy Scale—International*.

The Stepping Threshold Test sum score (DSE) correlated moderately with the BriefBEST (*r* = 0.388) and the TUG (*r* = −0.435). In addition, the STT sum score (DSE) correlated low with the 8LBS (*r* = 0.246) and Short FES-I (*r* = −0.104) and (Table 4). The Stepping Threshold Test subscores (DSE) correlated low (*r* = |−0.104| to |−0.279|) in 7 of 16 values, moderately in 6 values (*r* = 0.305 to |−0.447|), and did not correlate with the reference measures in 3 values (Table 4). The single-step thresholds of the DSE correlated low in 9 of 16 values (*r* = |−0.105| to |−0.238|), moderately in 2 values (*r* = |−0.342| to 0.415), and did not correlate with the reference measures in 5 values (Supplementary Material 4.1). The multiple stepping thresholds (DSE) correlated low in 13 of 16 values (*r* =0.106 to |−0.267|), moderately in 1 value (*r* = |−0.318|), and did not show correlations with the reference measures in 2 values (Supplementary Material 4.1). Correlation plots for visual inspection are presented in the appendices (Supplementary Material 3.2).

**Table 4:** Correlation between STT sum score and subscores (DSE) and reference measures.

	**STT-Thresholds**		**BriefBEST**	**TUG**	**8LBS**	**Short FES-I**
**Primary outcome**	STT sum score	r	0.388	−0.435	0.246	−0.104
		CI95	0.16–0.58	−0.61–0.21	0.01–0.46	−0.33–0.13
**Secondary outcome**	SS subscore	r	0.276	−0.354	0.055	−0.120
		CI95	0.04–0.48	−0.55–0.12	−0.18–0.29	−0.35–0.12
	MS subscore	r	0.377	−0.378	0.305	−0.068
		CI95	0.15–0.57	−0.57–0.15	0.07–0.51	−0.3–0.17
	AP subscore	r	0.430	−0.447	0.272	−0.249
		CI95	0.21–0.61	−0.62–0.23	0.04–0.48	−0.46–0.01
	ML subscore	r	0.227	−0.279	0.113	0.085
		CI95	−0.01–0.44	−0.49–0.04	−0.13–0.34	−0.15–0.31

*Sign., Two-tailed significance; SS, Single-step; MS, Multiple Stepping; AP, Anteroposterior; ML, Mediolateral; CI95, confidence interval of 95%; r, correlation coefficient rho, calculated by means of the Spearman-rank-correlation; TUG, Timed Up and Go Test; BriefBEST, Brief Balance Evaluations Systems Test; 8LBS, Eight level balance scale; FES-I, Short Falls Efficacy Scale—International*.

### Discriminative Validity

The Stepping Threshold Test sum score and subscores (ACE) showed no significant differences between fallers and non-fallers (Table 5). Significant differences were found in the single-step threshold backward, with advantages for the fallers compared with the non-fallers (*p* = 0.034) (Supplementary Material 2.2).

**Table 5:** Differences between non-fallers and fallers in the STT sum scores (ACE).

		**Non-fallers (n** **=** **38)**					**Fallers (n** **=** **32)**					
		**Mean**	**Median**	**IQR**	**Min**	**Max**	**Mean**	**Median**	**IQR**	**Min**	**Max**	***P*-value**
**Primary outcome**	STT sum score	25.61	27	7	12	36	26.22	26	8.5	15	37	0.897
**Secondary outcome**	SS subscore	9.05	9	3.25	4	14	9.56	9	3	6	14	0.571
	MS subscore	16.55	16.5	5.25	8	25	16.66	17	4.75	9	26	1.000
	AP subscore	10.71	10	4	4	18	11.25	10	5	5	21	0.647
	ML subscore	14.89	15	3.5	7	21	14.97	15	5.75	10	21	0.817

*SS, Single-step; MS, Multiple Stepping AP, Anteroposterior; ML, Mediolateral; Two-tailed p-value calculated by means of the Mann-Whitney-U test. The significance level was set to p < 0.05*.

The Stepping Threshold Test sum score and subscores (DSE) showed no significant differences (Table 6) between fallers and non-fallers. Significant differences were found in the single-step threshold right (*p* = 0.015) with higher thresholds for the non-fallers compared with the fallers (Supplementary Material 4.2). The subsequent ROC-analysis indicated an AUC of 0.634 (95CI = 0.511–0.775).

**Table 6:** Differences between non-fallers and fallers in the STT sum scores (DSE).

		**Non-fallers (n** **=** **38)**					**Fallers (n** **=** **32)**					
		**Mean**	**Median**	**IQR**	**Min**	**Max**	**Mean**	**Median**	**IQR**	**Min**	**Max**	***P*-value**
**Primary outcome**	STT sum score	35.5	35	7.25	26	44	35.13	35	7.75	27	43	0.799
**Secondary outcome**	SS subscore	12.68	12	3	6	18	12.32	12	3	8	16	0.501
	MS subscore	22.82	23	4.25	16	27	22.81	23.5	5	17	28	0.972
	AP subscore	14.29	14	3	7	20	14.31	14	4.75	9	19	0.976
	ML subscore	21.21	21.5	2	16	27	20.81	21.5	5	16	26	0.807

*SS, Single-step; MS, Multiple Stepping AP, Anteroposterior; ML, Mediolateral; Two-tailed p-value calculated by means of the Mann-Whitney-U test. The significance level was set to p < 0.05*.

### Interpretability of the STT

The Stepping Threshold Test sum score of both ACE and DSE showed no floor (0%) or ceiling effect (0%). The subscores of both the ACE and DSE also revealed no ceiling or floor effects (0–4.29%) (Supplementary Materials 5.1, 5.2). In both the ACE and DSE, floor effects occurred in the single-step thresholds forward (41.43–57.14%) and backward (20–21.43%) (Tables 7, 8). In the ACE ceiling effect occurred only in the multiple stepping threshold left (21.43%) (Table 7). In the DSE ceiling effects were observed for the multiple stepping threshold backward (54.3%), left (48.6%), and right (54.3%) (Table 8).

**Table 7:** Floor and ceiling effects of the STT (ACE).

	**Floor effect** ^**a**^		**Ceiling effect** ^**b**^	
	**Single step**	**Multiple step**	**Single step**	**Multiple step**
Forward	57.14%	14.29%	0.00%	0.00%
Backward	21.43%	1.43%	0.00%	21.43%
Left	5.71%	0.00%	0.00%	2.86%
Right	4.29%	0.00%	0.00%	10.00%

*Floor or ceiling effect exists if the value is above 15 %*.

a *Percentage of participants who reached the lowest level in the single or multiple stepping thresholds*.

b *Percentage of participants who reached the highest single or multiple stepping thresholds*.

**Table 8:** Floor and ceiling effects of the STT (DSE).

	**Floor effect** ^**a**^		**Ceiling effect** ^**b**^	
	**Single step**	**Multiple step**	**Single step**	**Multiple step**
Forward	41.43%	0.00%	0.00%	7.14%
Backward	20.00%	0.00%	0.00%	54.29%
Left	0.00%	0.00%	1.43%	48.57%
Right	1.43%	0.00%	2.86%	54.29%

*Floor or ceiling effect exists if the value is above 15 %*.

a *Percentage of participants who reached the lowest level in the single or multiple stepping thresholds*.

b *Percentage of participants who reached the highest single or multiple stepping thresholds*.

### Feasibility

In total, 1,593 of 1,680 (94.8 %) perturbations were applied. The test was terminated prematurely in 18 subjects (25%) with an average of 19.7 out of 25 applied perturbations (*SD* = 2.7). In the ACE, 17 of these 18 (94.44%; in total, 69 of 70, 98.57%) subjects had already reached all single-step thresholds and 10 of these 18 subjects (55.55%) had already reached all multiple stepping thresholds. In the DSE, 9 of these 18 (50%; in total, 61 of 70, 87.14%) subjects had already reached all single-step thresholds and none of these 18 subjects (0%) had already reached all multiple stepping thresholds. Accordingly, 62 of 70 (88.57%) participants reached all thresholds in the ACE and 52 of 70 (75%) participants reached all stepping thresholds in the DSE during the testing procedure. For five participants (7.14 %), fall thresholds were documented (mean perturbation = 21.4, *SD* = 2.3), whereas the earliest fall appeared in perturbation number 18 and the latest in perturbation 23. We were able to include every participant but one due to technical problems (98.59%) in the analysis of both ACE and DSE using the calculated thresholds. There were no adverse events, but some participants reported high stress levels and anxiety during the higher intensities of the STT.

## Discussion

This study is the first empiric investigation of the psychometric properties of the STT in fall-prone older adults. We provided evidence of the convergent validity of this reactive balance test with respect to fall risk and introduce a newly developed DSE to evaluate stepping behavior. Discriminative validity could not be demonstrated. Floor and ceiling effects were found in the original thresholds for ACE and DSE, but not in the sum scores and subscores. Completion rates of the STT indicated sufficient feasibility for the ACE, but not for the DSE.

### Convergent Validity

Previous studies reported correlations between reactive balance and measures of other balance domains between 0.03 and 0.691 (Crenshaw et al., 2018; Kiss et al., 2018; Handelzalts et al., 2019b). The Brief Balance Evaluation Systems Test is a testing battery that contains measures of all four balance domains (Marques et al., 2016) defined by Shumway-Cook and Woollacott (2017), i.e., static, dynamic, proactive, and reactive balance. Accordingly, we expected to find moderate correlations between the STT and global balance as measured by the BriefBEST. Thus, moderate correlations between the STT sum score and the BriefBEST of 0.413 (ACE) and 0.388 (DSE) confirmed our hypothesis related to convergent validity.

The meta-analysis by Kiss et al. (2018) found a low correlation between reactive balance and proactive balance (*r* = 0.14), but this result was based on only a single study (Owings et al., 2000). The study of Handelzalts et al. (2019b) performed the STT in 15 persons with stroke and correlated balance measures with the fall thresholds, i.e., the perturbation intensity that could not be compensated and led to unambiguous support by the harness. They found correlations of *r* = 0.691 between the STT and the Berg Balance Scale, a test battery that primarily consists of proactive balance items. Accordingly, we also hypothesized to find moderate correlations between measures of reactive and proactive balance. Correlations between the STT sum score and the TUG of *r* = −0.379 (ACE) and *r* = −0.435 (DSE) confirmed our hypothesis. Lower correlations in our study compared with the study of Handelzalts et al. (2019b) may be attributed to the different sample characteristics, i.e., stroke patients vs. older adults.

The study of Crenshaw et al. (2018) explored correlations between standing postural control and anteroposterior step and stepping thresholds and revealed low to moderate correlations (*r* = 0.21–0.38). The study of Kiss et al. (2018) included five studies in their analysis with respect to the relationship of static balance and reactive balance and found a correlation coefficient of *r* = 0.19. Accordingly, we expected low to moderate correlations between reactive balance and static balance in our study. Correlation coefficients between the SST sum scores and the 8LBS of *r* = 0.173 (ACE) and *r* = 0.246 (DSE) confirmed our hypothesis of a low to moderate the relationship between reactive and static balance measures.

In a recent study, Batcir et al. (2020) applied mediolateral perturbations in a comparable sample and found moderate correlations (single-step threshold: *r* = −0.398 and multiple stepping threshold: −0.302) between the single-step and multiple stepping thresholds and the fear of falling. The study of Crenshaw et al. (2018) applied anteroposterior perturbations and found low correlations (*r* = 0.19–0.20) of single-step thresholds and moderate correlations (*r* = 0.39–0.40) of multiple stepping thresholds with activity-specific balance confidence, a construct which is similar to fear of falling. Accordingly, we hypothesized to find low to moderate correlations between the STT sum score and the Short FES-I. We determined lower correlation coefficients of *r* = −0.108 (ACE) and *r* = −0.104 (DSE), which are, however, within the expected range. Interestingly, anteroposterior subscores were higher (ACE: *r* = −0.195 and DSE: *r* = −0.249) and mediolateral subscore did not indicate any correlation (ACE: *r* = −0.008 and DSE: *r* = 0.085). These findings are in line with experiences gained during the testing procedure that AP perturbations seemed to be the most uncomfortable especially for anxious participants. Anteroposterior step and stepping thresholds might be closer related to fear of falling since backward perturbations require a particular fast step reaction (Sturnieks et al., 2013). In addition, forward step and stepping motion is a very common lower extremity motion in daily life and is also addressed in the Short FES-I (Kempen et al., 2008). The absence of more and higher correlations can be explained by the fact, that the median Short FES-I score was very low in our study population. A reason may be that mainly individuals with a lower fear of falling were willing to participate in our study (recruitment bias).

These results are supplemented by numerous correlations between the reference measures, the STT subscores (ACE: 14 of 16 values, *r* = 0.102 to |−0.433|; DSE: 13 of 16 values, *r* = |−0.104| to |−0.447|) and the original single-step and multiple stepping thresholds (ACE: 26 of 32 values, *r* = 0.104 to |−0.390|; DSE: 25 of 32 values, *r* = |−0.105| to 0.415). Due to the high numbers of variables in our secondary outcomes the possibility of type-I error must be considered here. However, only the primary outcome, i.e., the STT sum score, was considered in hypothesis testing and secondary outcomes do not affect the conclusion of this study. In summary, our hypothesis regarding the convergent validity of the STT with other assessments of balance and fall risk was confirmed.

### Discriminative Validity

Our initial hypothesis regarding the discriminative validity of the STT could not be confirmed. None of the sum scores or subscores showed significant differences in the comparison of fallers and non-fallers. In the DSE, we found one original threshold, i.e., single-step threshold right, at which non-fallers performed significantly better than fallers. However, since we conducted several analyses for the same hypothesis, single results should be interpreted with caution and could be due to chance (Streiner and Norman, 2011). In addition, we also found a threshold in the ACE at which fallers performed significantly better. Several previous studies showed reactive step and stepping thresholds to be capable to distinguish between non-fallers and fallers (Hilliard et al., 2008; Batcir et al., 2020; Crenshaw et al., 2020). However, our results are aligned with other studies that could not show significant differences between non-fallers and fallers by means of reactive balance tests (Mille et al., 2013; Sturnieks et al., 2013; Fujimoto et al., 2015).

On one hand, the lack of significant results might be due to our inclusion criterion of fall proneness resulting in low heterogeneity between fallers and non-fallers. Although normal age-related physiological changes, balance deficits, and fear of falling are relevant to falls (Ambrose et al., 2013), we did not find any significant difference between fallers and non-fallers. On the other hand, retrospective fall assessment is accompanied by a risk of inaccurate data because of recall bias (Ganz et al., 2005), and prospective fall assessment is preferable. Previous studies compared non-fallers with recurrent fallers (at least two falls) (Balasubramanian et al., 2015; Lima et al., 2018; Batcir et al., 2020) to increase discriminatory power between the groups and to ensure that subjects are not classified as fall-prone because of an unavoidable event that leads to a fall, but because of endogenous factors that significantly increase fall risk. However, the number of recurrent fallers in our study sample was too small to allow this, and further studies with a higher number of recurrent fallers are needed. In addition, strong floor and ceiling effects had occurred that may have limited the validity of the test procedure. Determining a fall threshold, i.e., the level of perturbation at which participants fall into a harness system, as done in the study by Handelzalts et al. (2019b), could lead to benefits in terms of discriminative validity. However, in our study population, only five participants had experienced a fall into the harness system during test use, so statistical evaluation of this threshold was not possible.

### Interpretability

Neither for the sum score nor subscores floor or ceilings effects were found. However, strong floor and ceiling effects were observed in consideration of the individual step and stepping thresholds in both, the ACE and DSE. Since the criteria for whether a step is counted as such are more demanding in the DSE, it is plausible that stronger ceiling effects occurred here, whereas floor effects were more pronounced in the ACE. The greatest floor effects appeared in the single-step threshold forward. This threshold represented a forward displacement of the surface and thus a backward displacement of the CoM of the participants. Center of mass translations in the backward direction require a particular fast step reaction, as the location of the CoM is relatively close to the base-of-support border (Sturnieks et al., 2013) and muscular stabilization in this direction is more demanding (Hall and Jensen, 2002). In addition to the higher demand for this perturbation, the fact that the first perturbation was applied in this direction might have increased the floor effect even due to insecurity.

Even though we used the highest perturbation intensities that the utilized perturbation treadmill (Balance Tutor) is capable of, ceiling effects in the multiple stepping thresholds appeared in the DSE in all directions except in the forward translations. This is surprising, since mediolateral reactive stepping strategies, such as the cross-over step are also very demanding for older adults (Mille et al., 2013). Thus, depending on the target population higher intensities in mediolateral and backward surface translations might be necessary to evaluate multiple stepping thresholds with the DSE. Due to the limited system, one might consider other ways of increasing demand, e.g., limiting arm movements and reducing BoS in standing, but taking into account ecological validity (Reis and Judd, 2000) and the construct of reactive balance. Another potential improvement could be the inclusion of more levels of perturbation to expand the ability to stratify participants. However, this would increase the duration of the test and thus further increase the psychological and physical stress.

### Feasibility

Previous studies regarded feasibility as sufficient if at least 85% of the measurements were successful (Malmberg et al., 2002; Waninge et al., 2011). For a clinical setting, even a higher rate of completion than 85% would be desirable. In this study, 75% of the participants performed all perturbations, but more than half (55.55%) had already reached all thresholds in the ACE leading to sufficient completion rates for this evaluation strategy. In the DSE, none had already reached all thresholds, resulting in an insufficient completion rate of 75%. Accordingly, we presented preliminary evidence that the STT is feasible using ACE in the scientific setting. For the feasibility of the DSE, higher completion rates should be achieved for the multiple stepping thresholds.

Sufficient test completion rates are already present for the single-step thresholds (ACE: 98.57% and DSE: 87.14%). In this study, results for the Single-step subscore were similar or only slightly different to the STT sum score and the Multiple stepping subscore in both ACE and DSE. The study of Crenshaw et al. (2018) found correlations of 0.29–0.68 between anteroposterior single-step and multiple stepping thresholds. Future studies should examine whether there is a substantial benefit by multiple stepping thresholds compared with single-step thresholds that justify the significantly higher burden placed on participants during the assessment.

Since the perturbation treadmill and the camera system we used are commercially available, the test application is also transferable to other settings. To increase feasibility, especially in the DSE, stress and anxiety levels should be reduced for example by more extensive familiarization with the test prior to the actual test administration. Further studies are needed to investigate the different areas of feasibility such as acceptability, practicality, and implementation (Bowen et al., 2009) of the STT in the scientific and clinical setting. Since no adverse events occurred, the STT can be considered safe.

### ACE vs. DSE

When comparing ACE and DSE, we observed differences in the evaluation of 660 out of 1,593 (41.43%) applied perturbations. This high frequency of differences in the evaluations confirmed the need for a differentiated view of these two observer-based evaluation methods. For both evaluation strategies, namely the ACE and DSE, we presented evidence for convergent validity. Based on discriminative validity, neither DSE nor ACE shows advantages over the other evaluation strategy. The total number of correlating thresholds was slightly higher in the ACE compared with the DSE, the subscores, and the original thresholds. There are tendencies that the ACE might be more valid in mediolateral single-step thresholds and the DSE be more valid in anterior multiple-stepping thresholds (Supplementary Materials 2.1, 4.1). Furthermore, ceiling effects in the DSE suggested that that the full potential of this approach has not yet been exploited. Higher perturbation intensities could lead to an even more precise and differentiated assessment of reactive balance capacity, especially in the DSE, and to even clearer results regarding validity. In conclusion, we cannot make a clear recommendation on which evaluation strategy should be used in future assessments of reactive balance in community-dwelling, fall-prone older adults. Nonetheless, our results showed that a differentiated consideration of these two approaches is an important step on the way to a valid and feasible reactive balance test for this population. This will require further studies comparing the results of both approaches with other measurements of reactive balance and, if available, with a gold standard. To compare the utility of both approaches in assessing fall risk, prospective studies with higher numbers of participants and a less homogeneous population should be conducted.

### Limitations

A limitation of this study is the retrospective characterization of participants as fallers or non-fallers. The number of recurrent fallers in our sample was too small to conduct an analysis of such a subsample. While our STT protocol was unpredictable with respect to perturbation direction, the gradual increase of the perturbation intensity might have been predictable. While our results indicated convergent validity, future validation studies could use a specific reactive balance test as a reference measure.

### Recommendations for Future Research

Finally, we would like to provide recommendations based on our findings and experiences during the study process:

1. To avoid floor and ceiling effects, future studies should determine the optimal intensity in terms of magnitude, velocity, acceleration, duration of and the number of surface translations for each direction for different populations. In community-dwelling, fall-prone older adults this includes both higher and lower magnitudes than applied in this study. On the same note, care must be taken to avoid excessive demands and to ensure safety. Particular attention should be paid to the proper balance of mediolateral, anterior, and posterior perturbations intensities.
2. In consideration of the floor effects that occurred only in anteroposterior perturbations as well as the described associations between anteroposterior step and stepping thresholds and fear of falling, we recommend either starting the STT with mediolateral perturbations or with a lower intensity.
3. When using unexpected perturbations, participant anxiety and stress levels should be considered when planning studies. Future investigations should refrain from extending the duration of the test, e.g., by a higher number of applied perturbations, to avoid a further increase in the stress level and a resulting physical and psychological overload of the participants. In this context, we would like to point out psychological consequences of fall experiences such as post-fall anxiety syndrome (Rubenstein, 2006) and advice against pushing fall thresholds in older adults at risk for falls, especially those with previous fall experiences.
4. Perform perturbation treadmill familiarization consisting of treadmill walking, being caught by the harness, and small perturbations to keep stress and anxiety levels as low as possible. At the same time, the learning effect must be considered and kept as low as possible when performing a reactive balance test.
5. The calculation of sum scores, as presented in this study, contributes to higher validity and should be considered as a further variant with regard to the analysis of step and stepping thresholds.
6. Further validation studies are needed that compare results of the STT with other measures of reactive balance, e.g., the lean and release test (Inness et al., 2015).

## Conclusion

The Stepping Threshold Test is a promising assessment tool of reactive balance applicable on commercially available computerized treadmill systems. We demonstrated evidence for convergent validity in fall-prone older adults. Furthermore, we presented a new approach with respect to the evaluation of reactive step and stepping behavior and gave concrete recommendations for further application of the test. Although current evidence is not sufficient to use the STT as fall risk assessment, we recommend further research in order to optimize the test protocol with respect to different target populations. If this succeeds, the STT has the potential to be applied as a regular, valid assessment for reactive balance in the clinical setting.

## Data Availability Statement

The datasets presented in this study can be found in online repositories. The names of the repository/repositories and accession number(s) can be found below: https://heibox.uni-heidelberg.de/d/cfd40c46c8be43b1b519.

## Ethics Statement

The studies involving human participants were reviewed and approved by Ethics Committee of Heidelberg University (reference: AZ Schwe 2019 /1-2). The patients/participants provided their written informed consent to participate in this study.

## Author Contributions

MA was involved in data analysis and interpretation and drafting of the manuscript. LB was involved in the conception, experimental design, data acquisition, data interpretation, and drafting of the manuscript. ML was involved in data interpretation and critical revision of the manuscript. MS was involved in the conception, data interpretation, and drafting of the manuscript. All authors contributed to the article and approved the submitted version.

## Funding

This study was embedded in the interventional study for disorder training registered at clinicaltrials.gov (study registry number: NCT04087512). The mentioned study is supported by the Klaus Tschira Foundation. The responsibility for the content of this paper lies with the authors. The funders did not take any part in this work.

## Conflict of Interest

The authors declare that the research was conducted in the absence of any commercial or financial relationships that could be construed as a potential conflict of interest.

## Publisher's Note

All claims expressed in this article are solely those of the authors and do not necessarily represent those of their affiliated organizations, or those of the publisher, the editors and the reviewers. Any product that may be evaluated in this article, or claim that may be made by its manufacturer, is not guaranteed or endorsed by the publisher.
