# Supplementary material for: The Stepping Threshold Test for Reactive Balance: Validation of Two Observer-Based Evaluation Strategies to Assess Stepping Behavior in Fall-Prone Older Adults
Source: Front Sports Act Living. 2021 Oct 11;3:715392. doi: 10.3389/fspor.2021.715392 (PMC8542787; doi:10.3389/fspor.2021.715392)
Supplement: Supplementary file 1 [file Data_Sheet_1.zip › Supplement 1.DOCX]

Supplementary Material

Supplement 1. Random order of perturbations and time intervals between

| **Level of Perturbation** | **Direction of surface translation** | **Time intervals between perturbations** |
| --- | --- | --- |
| *Level 1* | Forward | 16.5 sec |
|  | Left | 12.5 sec |
|  | Right | 10 sec |
|  | Backward | 10 sec |
| *Level 2* | Right | 11.5 sec |
|  | Forward | 10.5 sec |
|  | Left | 11.5 sec |
|  | Backward | 11.5 sec |
| *Level 3* | Backward | 11.5 sec |
|  | Right | 12.5 sec |
|  | Forward | 11.5 sec |
|  | Left | 13.5 sec |
| *Level 4* | Right | 14.5 sec |
|  | Backward | 12.5 sec |
|  | Left | 14.5 sec |
|  | Left (Not rated) | 14.5 sec |
|  | Forward | 14.5 sec |
| *Level 5* | Forward | 14.5 sec |
|  | Left | 17.5 sec |
|  | Backward | 14.5 sec |
|  | Right | 19.5 sec |
| *Level 6* | Backward | 16.5 sec |
|  | Right | 19.5 sec |
|  | Forward | 16.5 sec |
|  | Left | X |

Sec.: seconds; X: No time interval, since no further perturbations followed.
