# Supplementary material for: The Stepping Threshold Test for Reactive Balance: Validation of Two Observer-Based Evaluation Strategies to Assess Stepping Behavior in Fall-Prone Older Adults
Source: Front Sports Act Living. 2021 Oct 11;3:715392. doi: 10.3389/fspor.2021.715392 (PMC8542787; doi:10.3389/fspor.2021.715392)
Supplement: Supplementary file 1 [file Data_Sheet_1.zip › Supplement 2.1, 2.2.DOCX]

Supplementary Material

Supplement 2.1 Correlation between STT (ACE) and reference measures

|  | **STT-Thresholds** |  | **Brief BEST** | **TUG** | **8LBS** | **Short FES-I** |
| --- | --- | --- | --- | --- | --- | --- |
| Antero-posterior | Single Step Forward | r | 0.289 | -0.275 | 0.107 | -0.167 |
|  |  | CI95 | 0.05 - 0.49 | -0.48 - -0.04 | -0.13 - 0.33 | -0.39 - 0.07 |
|  | Single Step Backward | r | 0.364 | -0.293 | 0.234 | -0.192 |
|  |  | CI95 | 0.13 - 0.56 | -0.5 - -0.06 | 0 - 0.45 | -0.41 - 0.05 |
| Medio-lateral | Single Step Left | r | 0.300 | -0.390 | 0.134 | 0.134 |
|  |  | CI95 | 0.06 - 0.5 | -0.58 - -0.16 | -0.11 - 0.36 | -0.11 - 0.36 |
|  | Single Step Right | r | 0.324 | -0.319 | 0.043 | 0.046 |
|  |  | CI95 | 0.09 - 0.52 | -0.52 - -0.08 | -0.19 - 0.28 | -0.19 - 0.28 |
| Antero-posterior | Multiple Stepping Forward | r | 0.290 | -0.292 | 0.128 | -0.140 |
|  |  | CI95 | 0.05 - 0.5 | -0.5 - -0.06 | -0.11 - 0.35 | -0.36 - 0.1 |
|  | Multiple Stepping Backward | r | 0.237 | -0.147 | 0.104 | -0.080 |
|  |  | CI95 | 0 - 0.45 | -0.37 - 0.09 | -0.13 - 0.33 | -0.31 - 0.16 |
| Medio-lateral | Multiple Stepping Left | r | 0.134 | -0.123 | 0.209 | -0.140 |
|  |  | CI95 | -0.11 - 0.36 | -0.35 - 0.12 | -0.03 - 0.43 | -0.36 - 0.1 |
|  | Multiple Stepping Right | r | 0.309 | -0.256 | 0.025 | -0.061 |
|  |  | CI95 | 0.07 - 0.51 | -0.47 - -0.02 | -0.21 - 0.26 | -0.29 - 0.18 |

CI95: confidence interval of 95%; r: correlation coefficient rho, calculated by means of the Spearman-rank-correlation; TUG: Timed Up and Go Test; Brief BEST: Brief Balance Evaluations Systems Test; 8LBS: Eight level balance scale; FES-I: Short Falls Efficacy Scale – International

Supplement 2.2 Differences between non-fallers and fallers in the STT (ACE)

|  | Non-fallers (n=38) | | | | |  | Fallers (n=32) | | | | |  |  |
| --- | --- | --- | --- | --- | --- | --- | --- | --- | --- | --- | --- | --- | --- |
|  | Mean | Median | IQR | Min. | Max. |  | Mean | Median | IQR | Min. | Max. |  | Sign. |
| Single Step Forward | 1.58 | 1 | 1 | 1 | 3 |  | 1.53 | 1 | 1 | 1 | 4 |  | 0.862 |
| Single Step Backward | 1.97 | 2 | 2 | 1 | 3 |  | 2.38 | 2 | 1 | 1 | 4 |  | 0.034* |
| Single Step Left | 2.61 | 3 | 1 | 1 | 4 |  | 2.97 | 3 | 1 | 2 | 7 |  | 0.468 |
| Single Step Right | 2.89 | 3 | 2 | 1 | 5 |  | 2.91 | 3 | 1 | 2 | 7 |  | 0.598 |
| Multiple Stepping Forward | 3.16 | 3 | 2 | 1 | 6 |  | 2.75 | 2 | 1 | 1 | 7 |  | 0.075 |
| Multiple Stepping Backward | 4.00 | 4 | 2 | 1 | 7 |  | 4.44 | 4 | 2 | 2 | 7 |  | 0.110 |
| Multiple Stepping Left | 4.66 | 5 | 1 | 2 | 7 |  | 5.66 | 7 | 3 | 2 | 7 |  | 0.980 |
| Multiple Stepping Right | 4.74 | 5 | 1 | 2 | 7 |  | 5.00 | 5 | 2 | 2 | 7 |  | 0.831 |

Sign.: Two-tailed significance calculated by means of the Mann-Whitney-U test. Significance level was set to p < 0.05. ; * p < 0.05
