# Supplementary material for: The Stepping Threshold Test for Reactive Balance: Validation of Two Observer-Based Evaluation Strategies to Assess Stepping Behavior in Fall-Prone Older Adults
Source: Front Sports Act Living. 2021 Oct 11;3:715392. doi: 10.3389/fspor.2021.715392 (PMC8542787; doi:10.3389/fspor.2021.715392)
Supplement: Supplementary file 1 [file Data_Sheet_1.zip › Supplement 3.1, 3.2.DOCX]

Supplementary Material

**Supplement 3.1 Scatterplots – Correlations between STT (ACE) and reference measures: S. 2 – 52**

Scatter plot 1 – 32: Orginal Thresholds (ACE)

Scatter plot 33 – 48: Subscores (ACE)

Scatter plot 49 – 52: STT Sum scores (ACE)

**Supplement 3.2 Scatterplots – Correlations between STT (DSE) and reference measures: S. 53 - 104**

Scatter plot 53 – 84: Orginal Thresholds (ACE)

Scatter plot 85 – 100: Subscores (ACE)

Scatter plot 101 – 104: STT Sum scores (ACE)

**Supplement 3.1 Scatterplots – Correlations between STT (ACE) and reference measures**


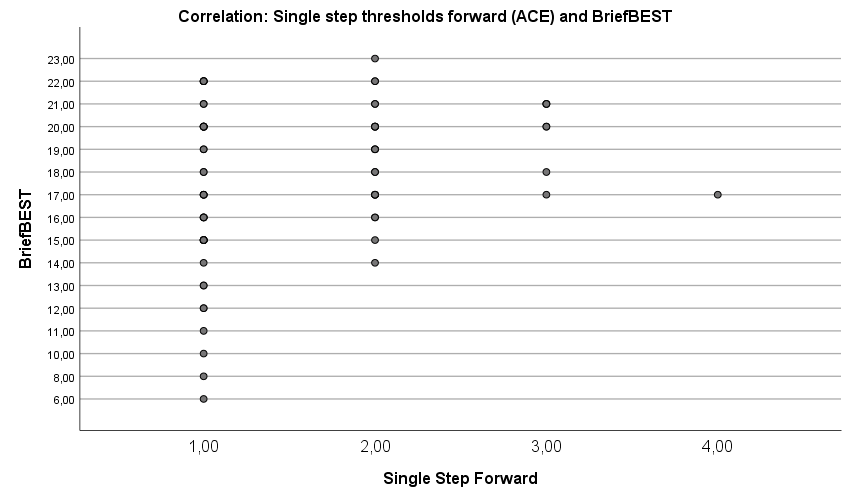


Scatter plot 1. ACE: All-step-count evaluation. BriefBEST: Brief Balance Evaluation Systems Test.


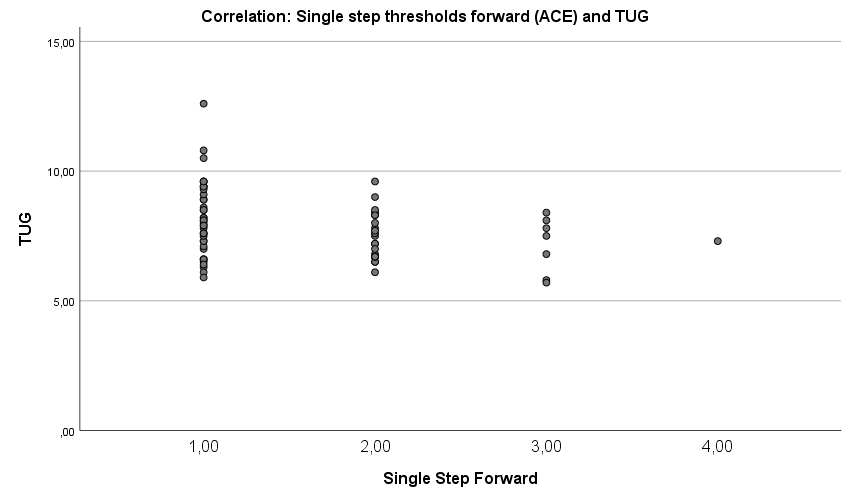


Scatter plot 2. ACE: All-step-count evaluation. TUG: Timed Up and Go


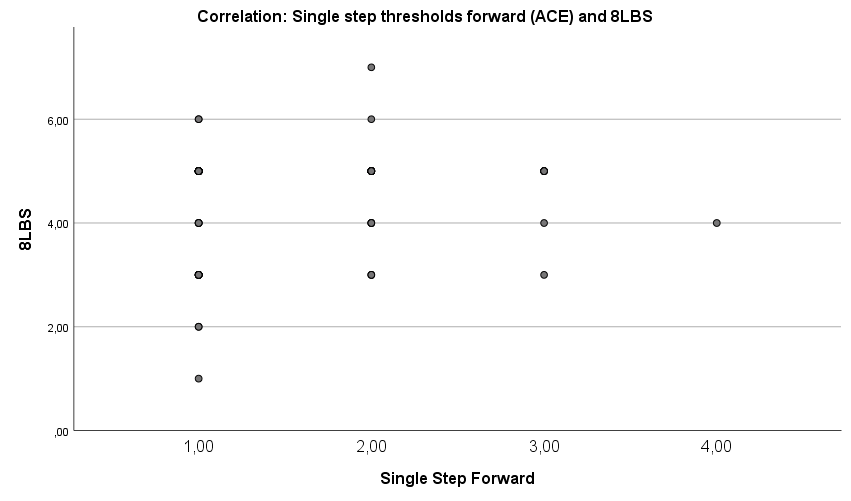


Scatter plot 3. ACE: All-step-count evaluation. 8LBS: 8-level balance scale


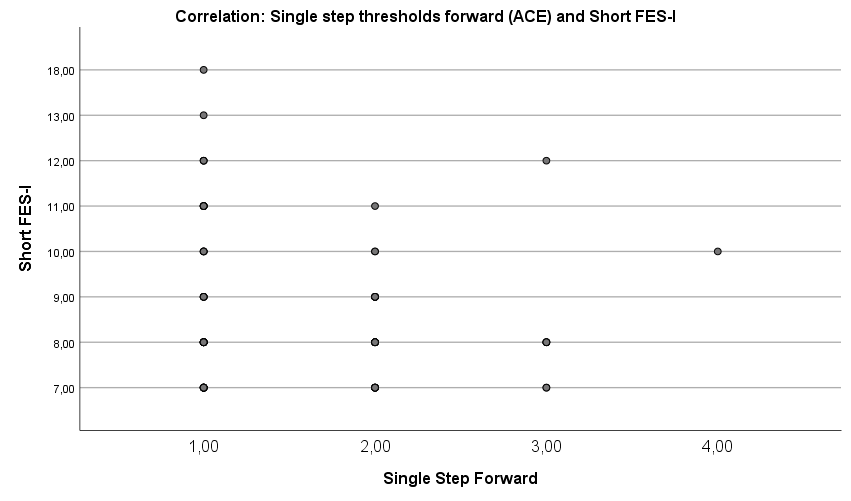


Scatter plot 4. ACE: All-step-count evaluation. Short FES-I: Short Falls Efficacy Scale – International.


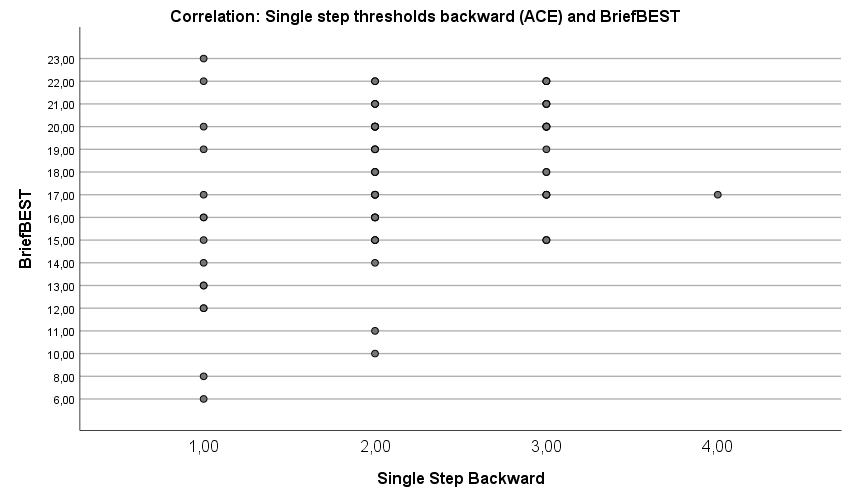


Scatter plot 5. ACE: All-step-count evaluation. BriefBEST: Brief Balance Evaluation Systems Test.


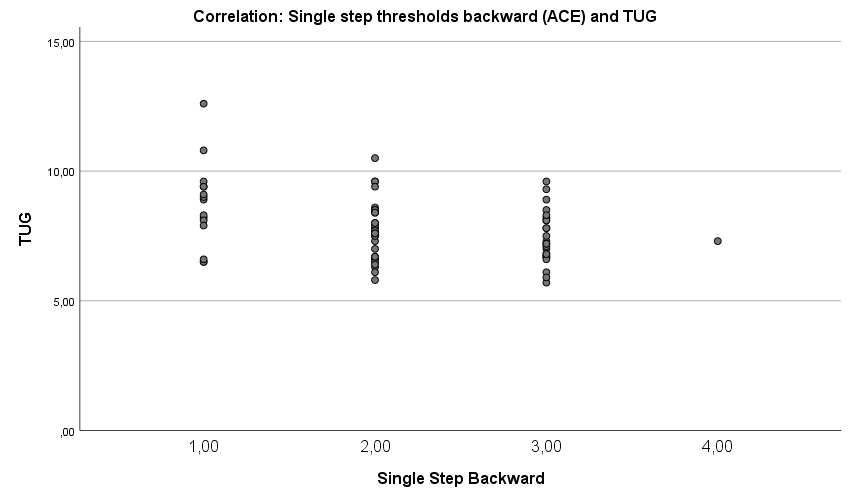


Scatter plot 6. ACE: All-step-count evaluation. TUG: Timed Up and Go


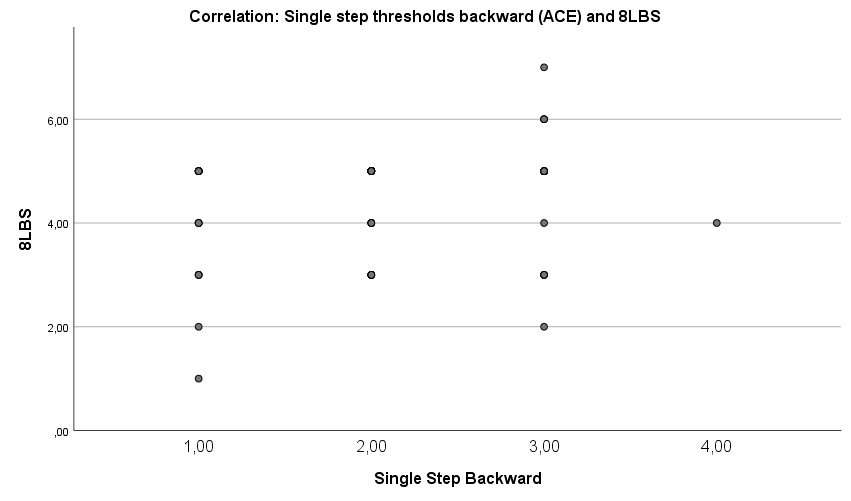


Scatter plot 7. ACE: All-step-count evaluation. 8LBS: 8-level balance scale


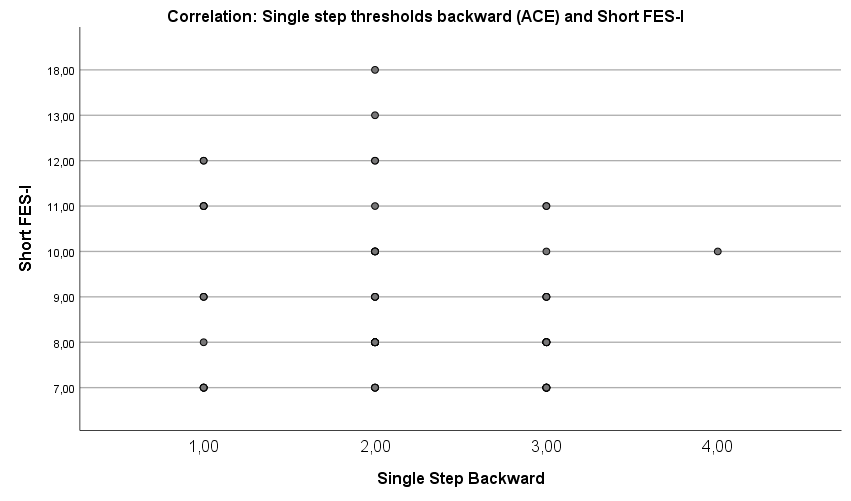


Scatter plot 8. ACE: All-step-count evaluation. Short FES-I: Short Falls Efficacy Scale – International.


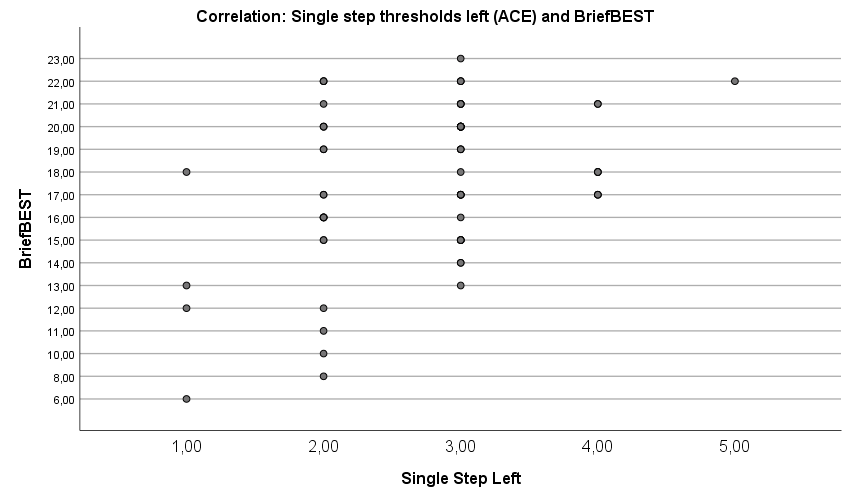


Scatter plot 9. ACE: All-step-count evaluation. BriefBEST: Brief Balance Evaluation Systems Test.


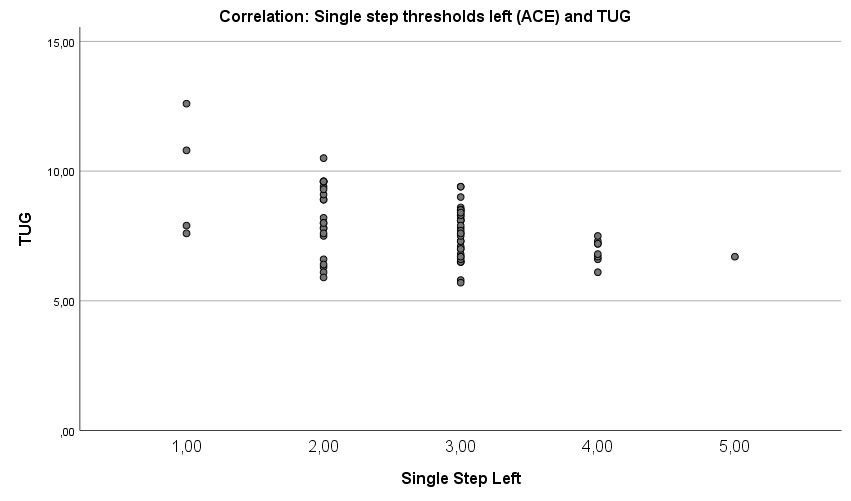


Scatter plot 10. ACE: All-step-count evaluation. TUG: Timed Up and Go


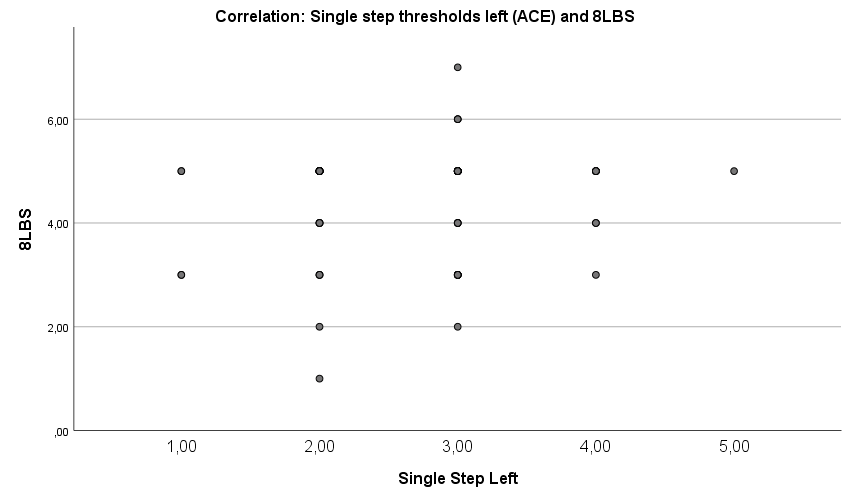


Scatter plot 11. ACE: All-step-count evaluation. 8LBS: 8-level balance scale


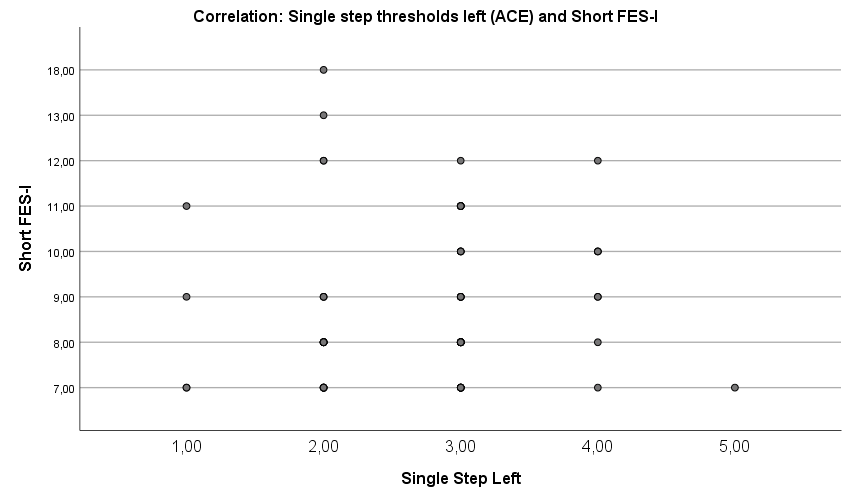


Scatter plot 12. ACE: All-step-count evaluation. Short FES-I: Short Falls Efficacy Scale – International.


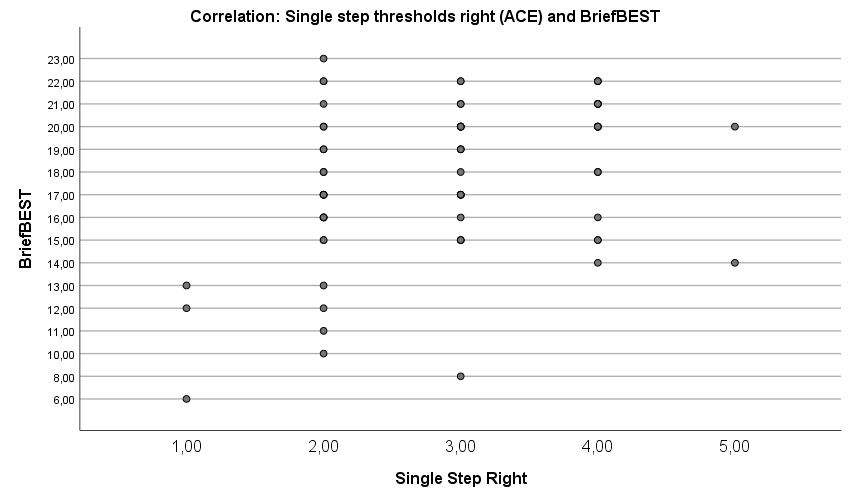


Scatter plot 13. ACE: All-step-count evaluation. BriefBEST: Brief Balance Evaluation Systems Test


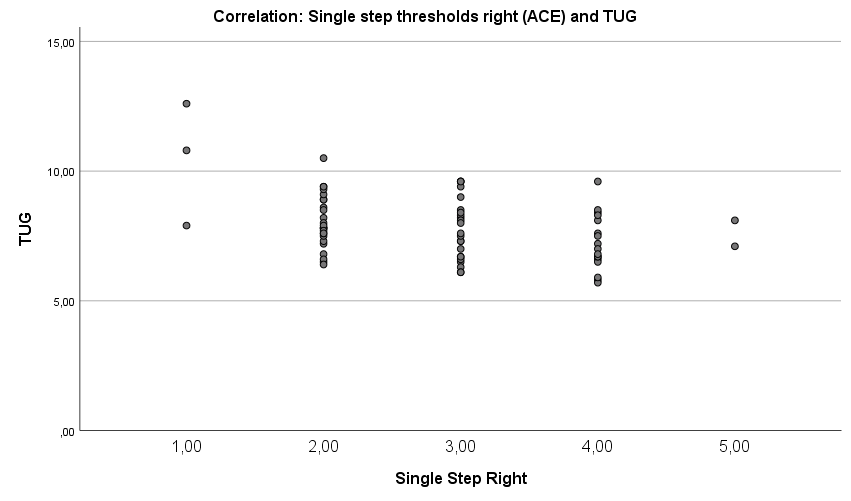


Scatter plot 14. ACE: All-step-count evaluation. TUG: Timed Up and Go


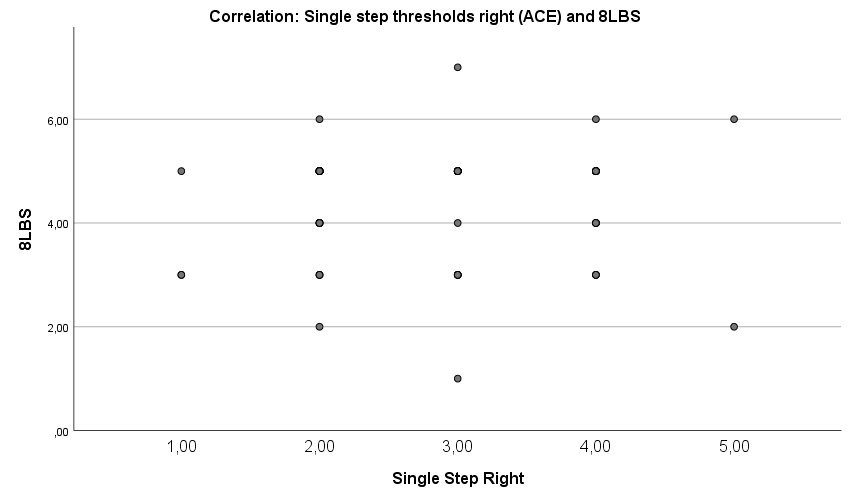


Scatter plot 15. ACE: All-step-count evaluation. 8LBS: 8-level balance scale


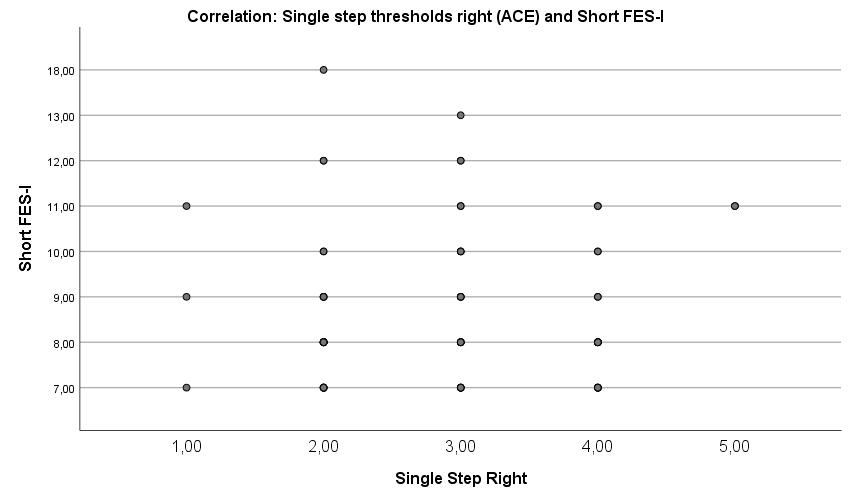


Scatter plot 16. ACE: All-step-count evaluation. Short FES-I: Short Falls Efficacy Scale – International.


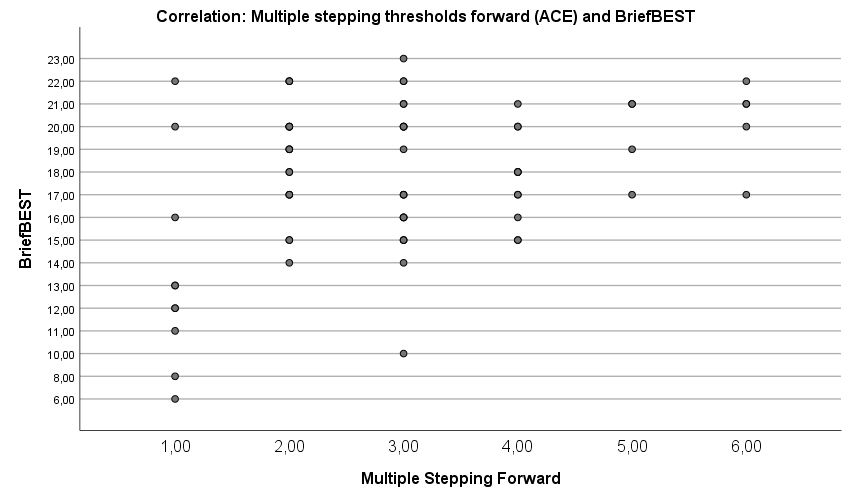


Scatter plot 17. ACE: All-step-count evaluation. BriefBEST: Brief Balance Evaluation Systems Test


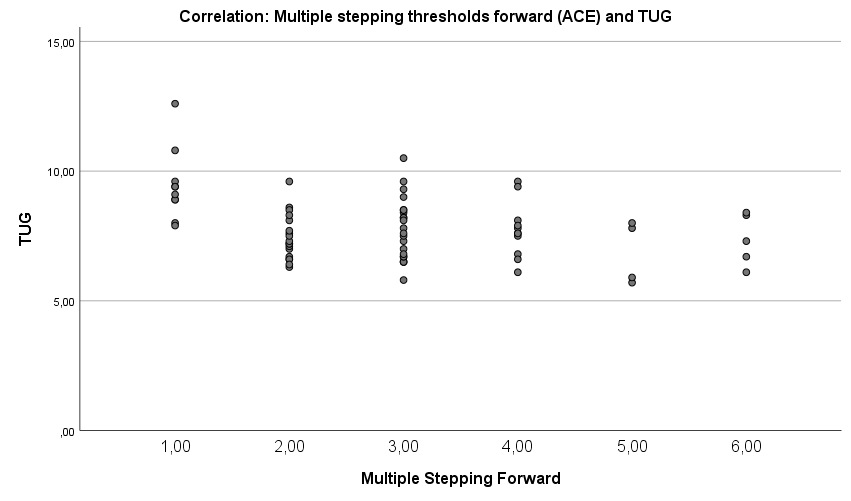


Scatter plot 18. ACE: All-step-count evaluation. TUG: Timed Up and Go


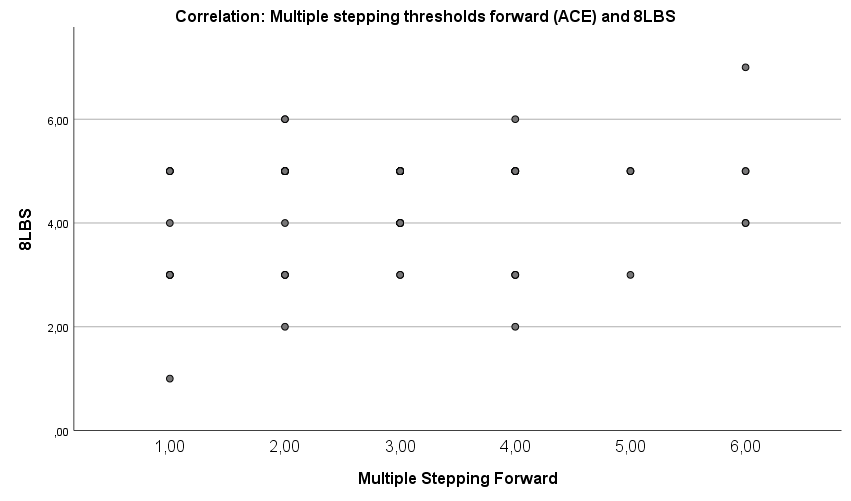


Scatter plot 19. ACE: All-step-count evaluation. 8LBS: 8-level balance scale


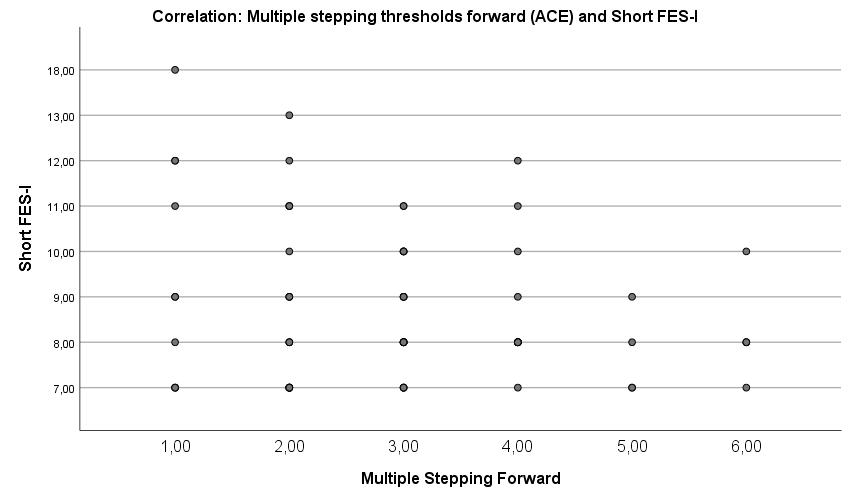


Scatter plot 20. ACE: All-step-count evaluation. Short FES-I: Short Falls Efficacy Scale – International.


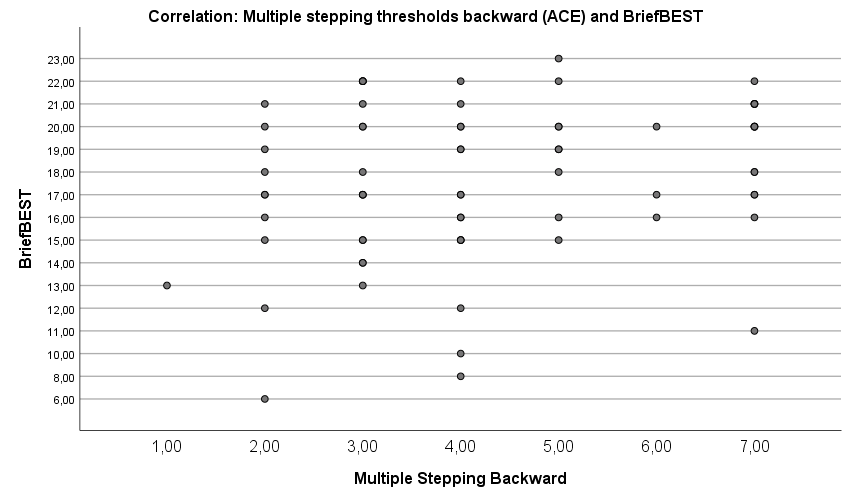


Scatter plot 21. ACE: All-step-count evaluation. BriefBEST: Brief Balance Evaluation Systems Test


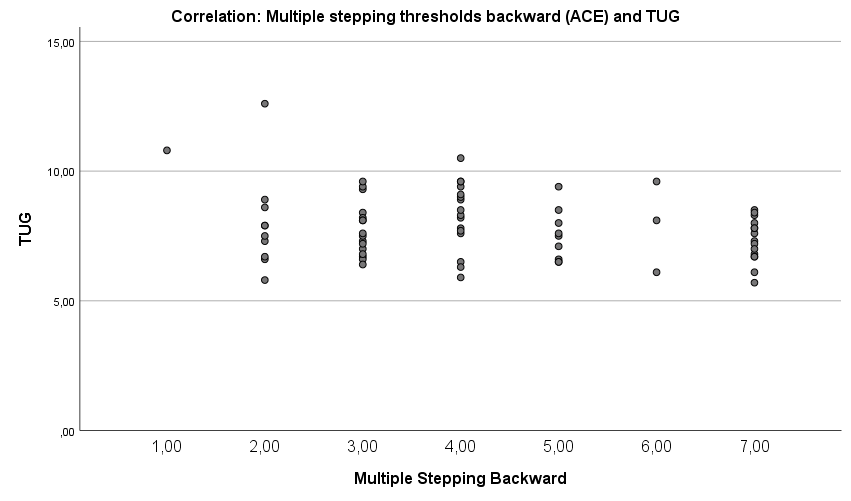


Scatter plot 22. ACE: All-step-count evaluation. TUG: Timed Up and Go


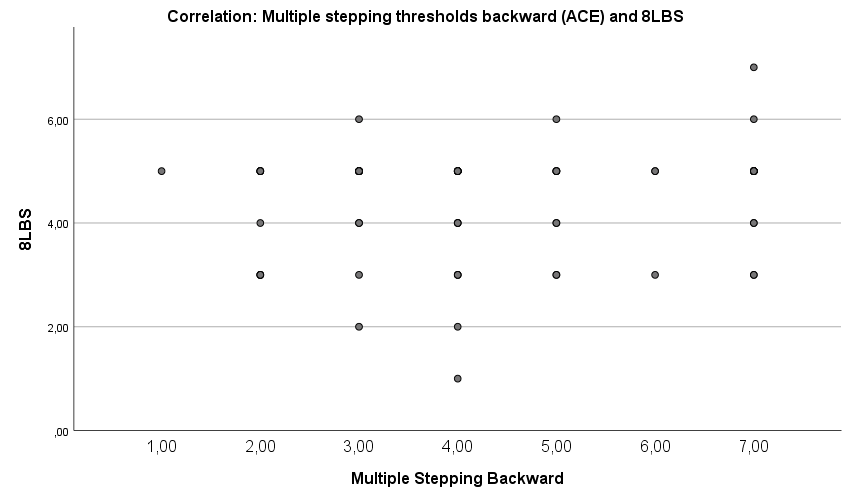


Scatter plot 23. ACE: All-step-count evaluation. 8LBS: 8-level balance scale


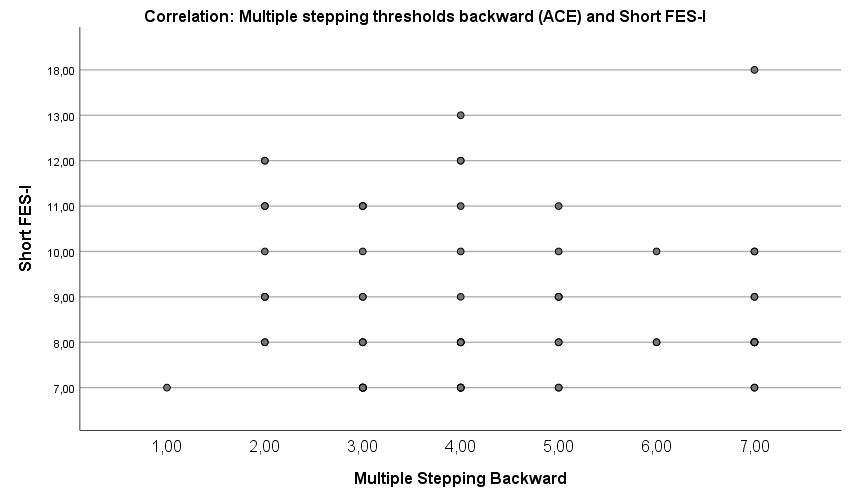


Scatter plot 24. ACE: All-step-count evaluation. Short FES-I: Short Falls Efficacy Scale – International.


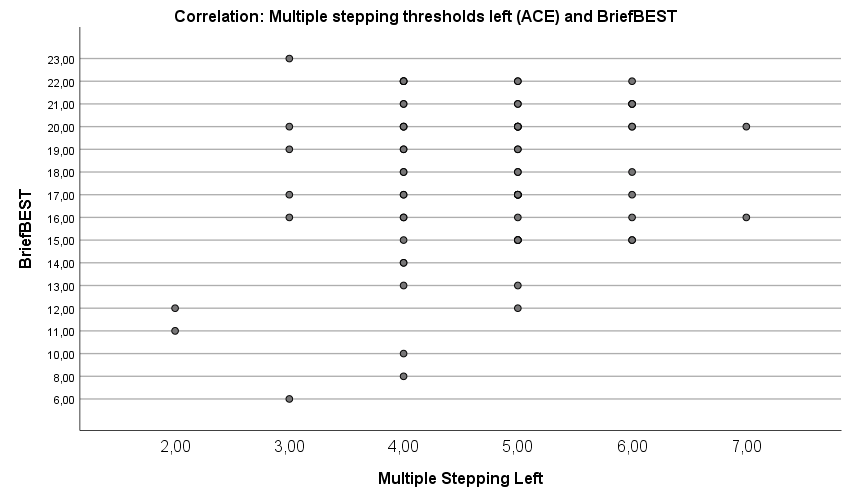


Scatter plot 25. ACE: All-step-count evaluation. BriefBEST: Brief Balance Evaluation Systems Test


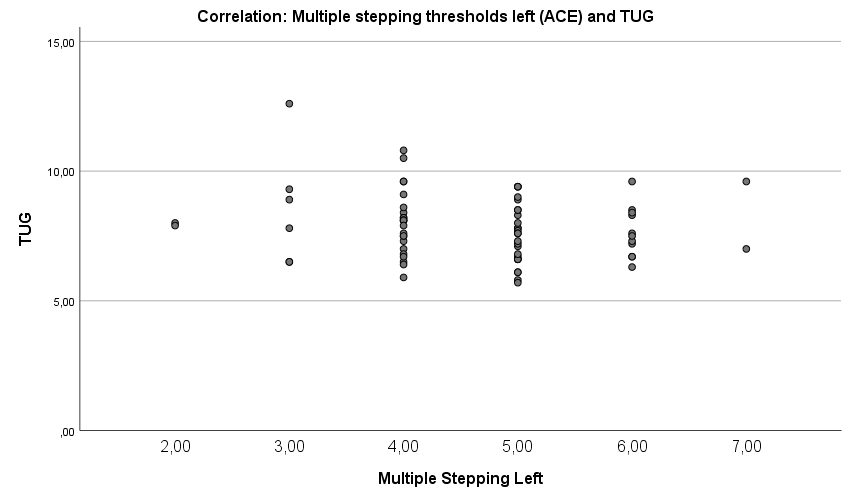


Scatter plot 26. ACE: All-step-count evaluation. TUG: Timed Up and Go


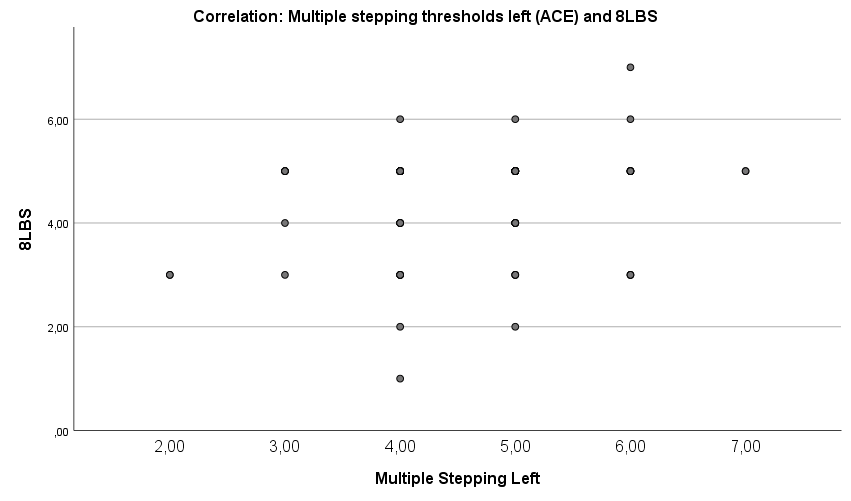


Scatter plot 27. ACE: All-step-count evaluation. 8LBS: 8-level balance scale


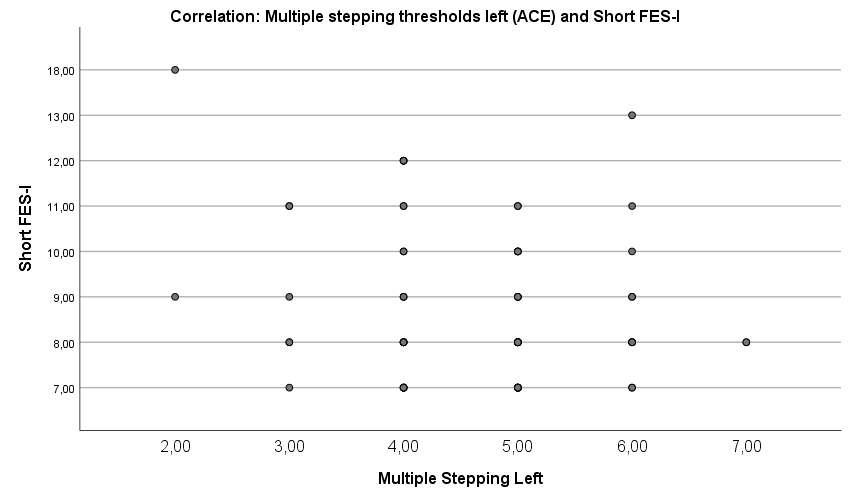


Scatter plot 28. ACE: All-step-count evaluation. Short FES-I: Short Falls Efficacy Scale – International.


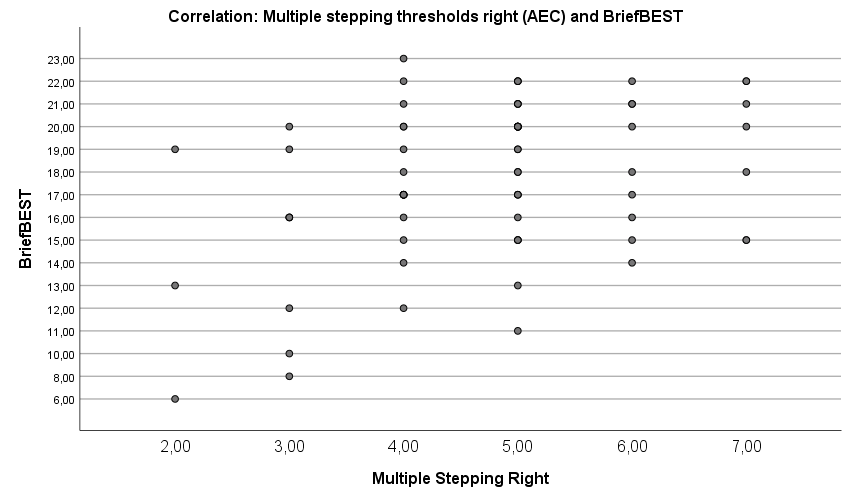


Scatter plot 29. ACE: All-step-count evaluation. BriefBEST: Brief Balance Evaluation Systems Test


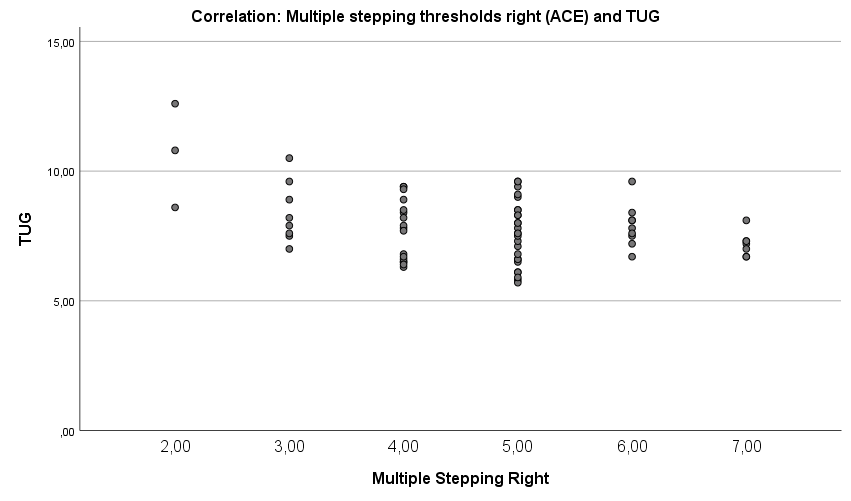


Scatter plot 30. ACE: All-step-count evaluation. TUG: Timed Up and Go


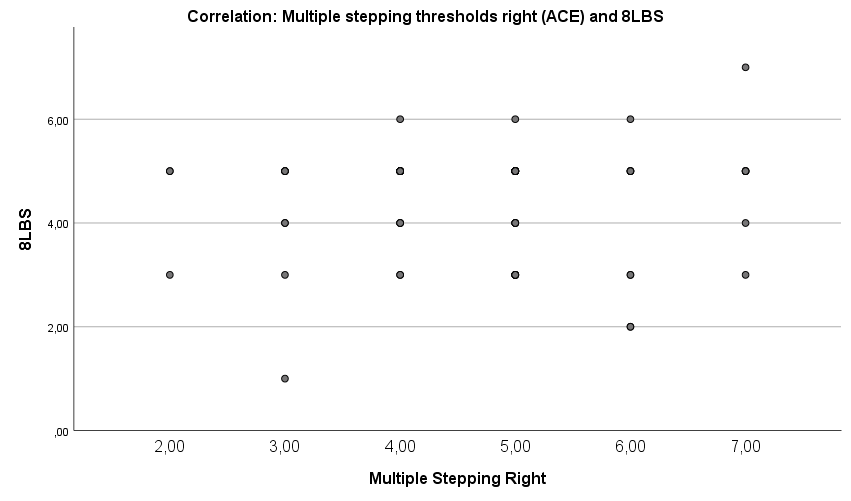


Scatter plot 31. ACE: All-step-count evaluation. 8LBS: 8-level balance scale


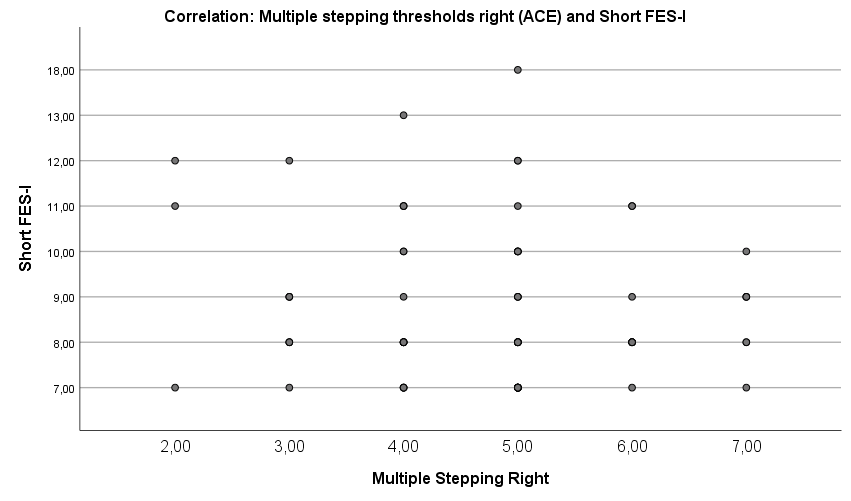


Scatter plot 32. ACE: All-step-count evaluation. Short FES-I: Short Falls Efficacy Scale – International.


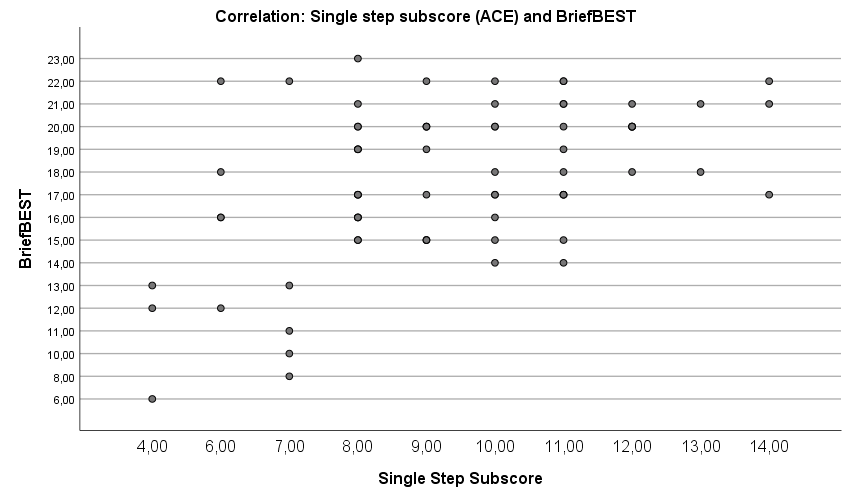


Scatter plot 33. ACE: All-step-count evaluation. BriefBEST: Brief Balance Evaluation Systems Test


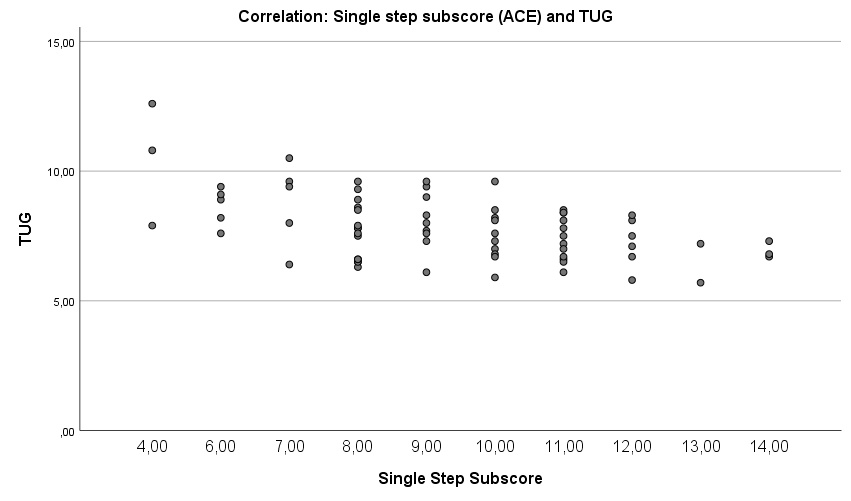


Scatter plot 34. ACE: All-step-count evaluation. TUG: Timed Up and Go


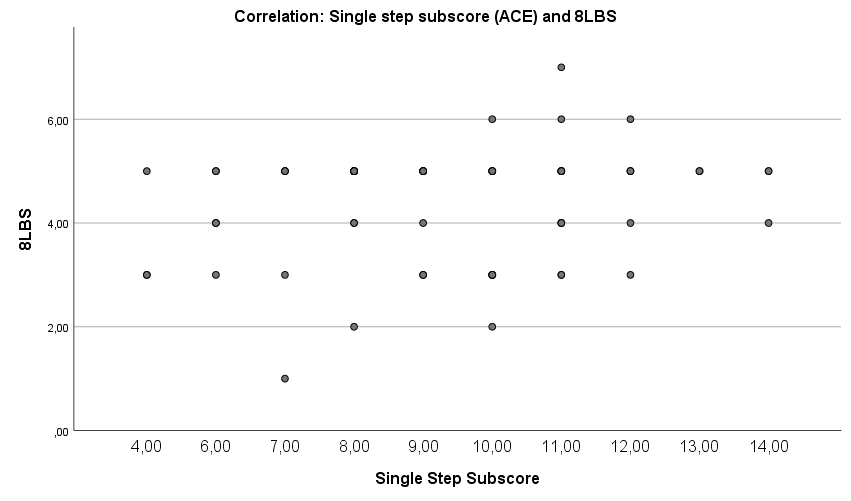


Scatter plot 35. ACE: All-step-count evaluation. 8LBS: 8-level balance scale


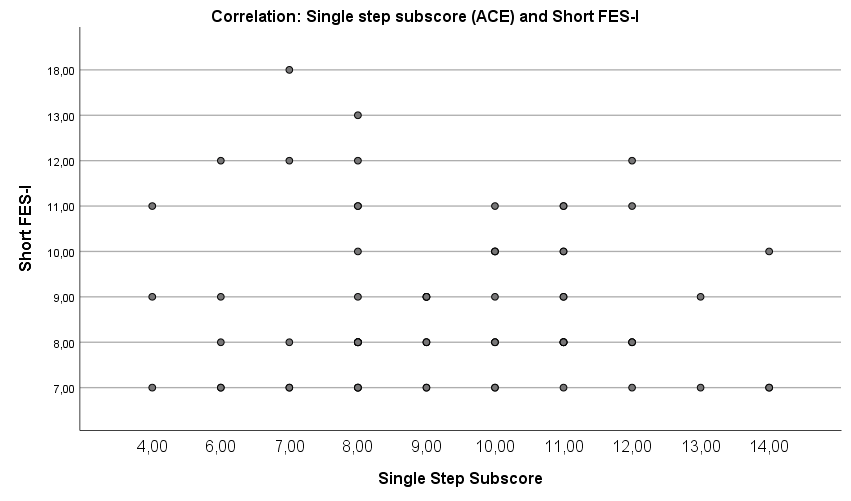


Scatter plot 36. ACE: All-step-count evaluation. Short FES-I: Short Falls Efficacy Scale – International


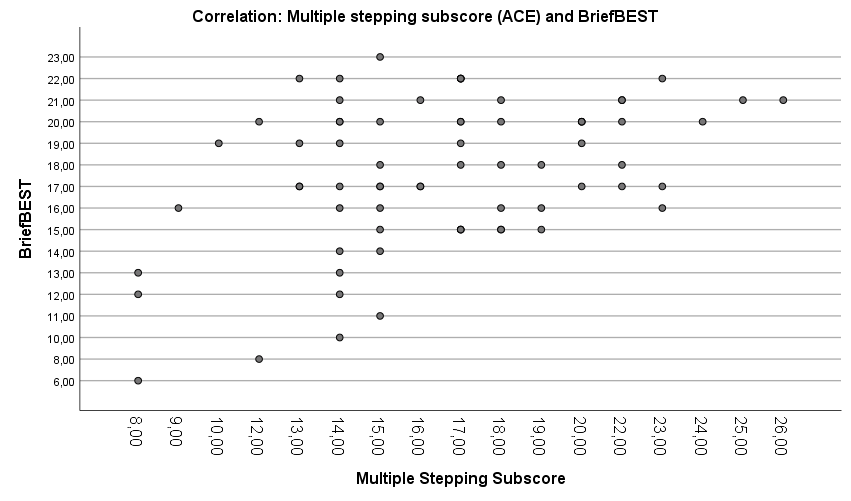


Scatter plot 37. ACE: All-step-count evaluation. BriefBEST: Brief Balance Evaluation Systems Test


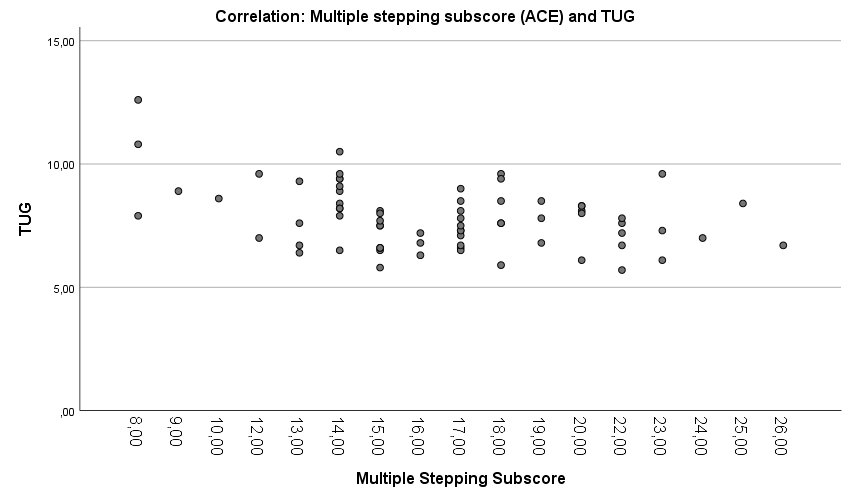


Scatter plot 38. ACE: All-step-count evaluation. TUG: Timed Up and Go


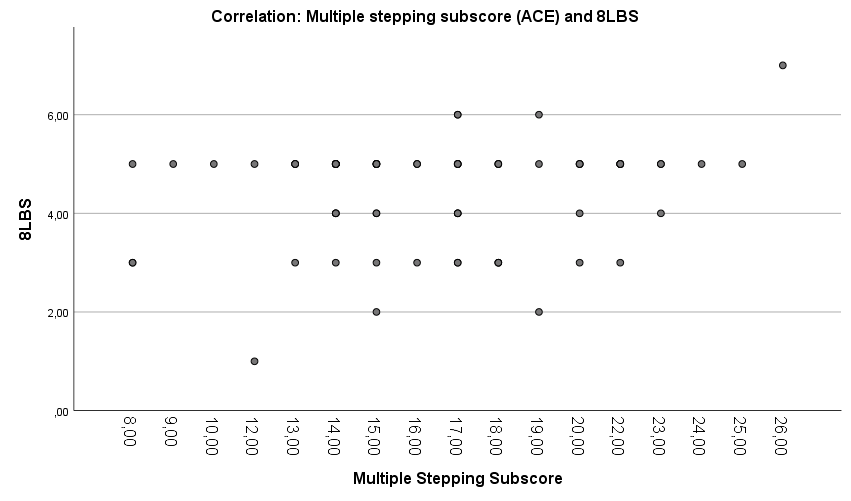


Scatter plot 39. ACE: All-step-count evaluation. 8LBS: 8-level balance scale


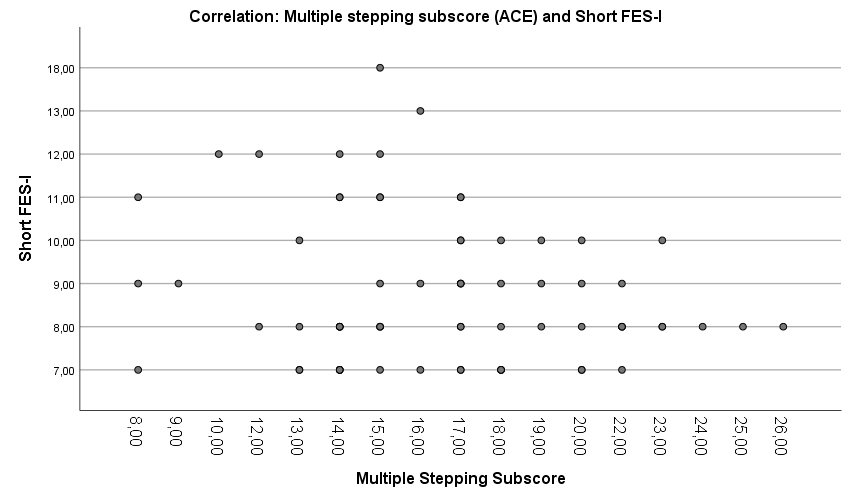


Scatter plot 40. ACE: All-step-count evaluation. Short FES-I: Short Falls Efficacy Scale – International


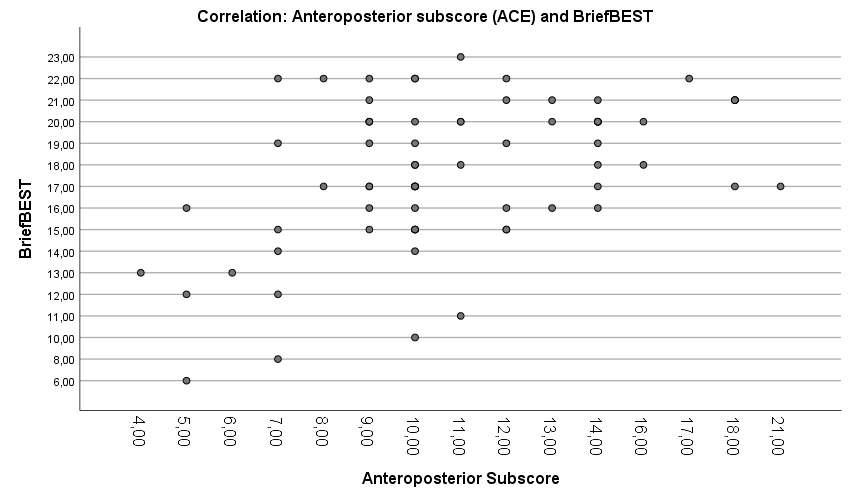


Scatter plot 41. ACE: All-step-count evaluation. BriefBEST: Brief Balance Evaluation Systems Test


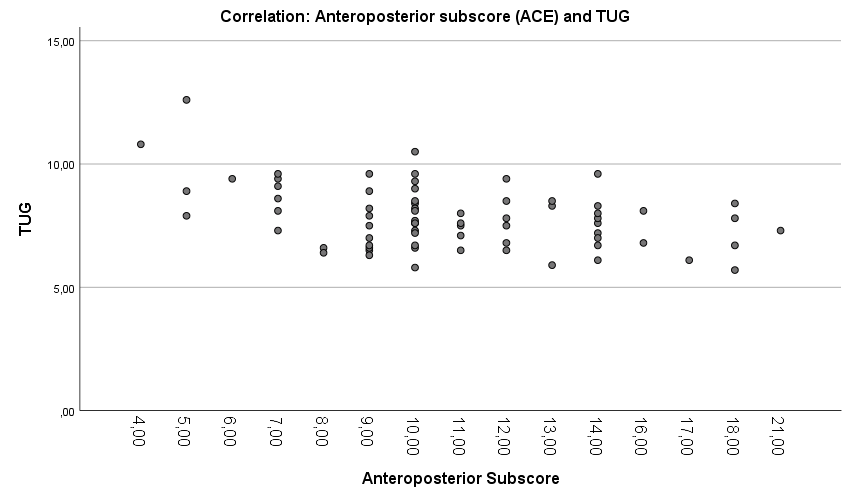


Scatter plot 42. ACE: All-step-count evaluation. TUG: Timed Up and Go


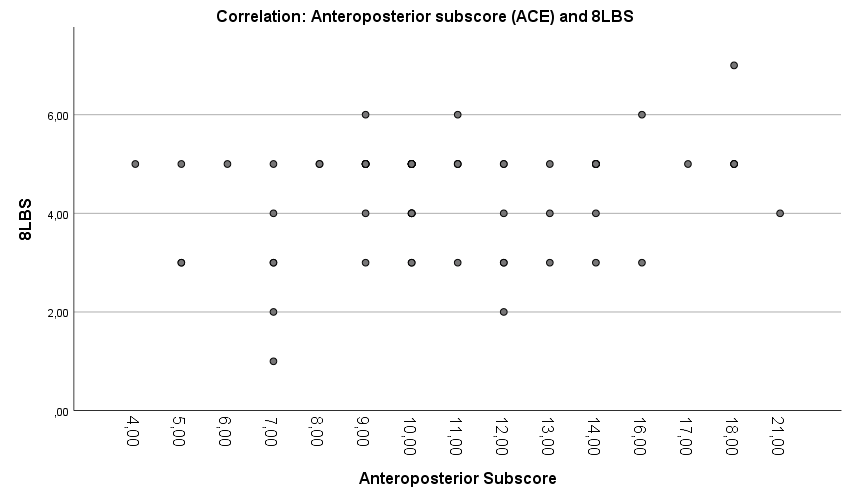


Scatter plot 43. ACE: All-step-count evaluation. 8LBS: 8-level balance scale


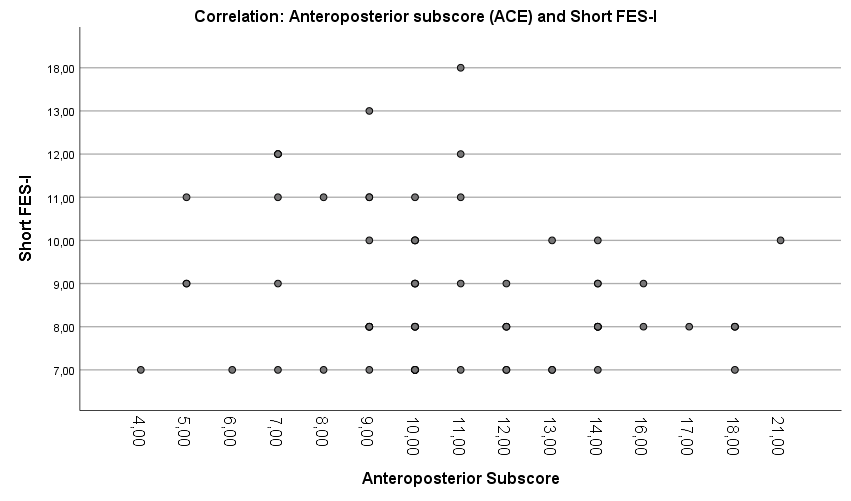


Scatter plot 44. ACE: All-step-count evaluation. Short FES-I: Short Falls Efficacy Scale – International


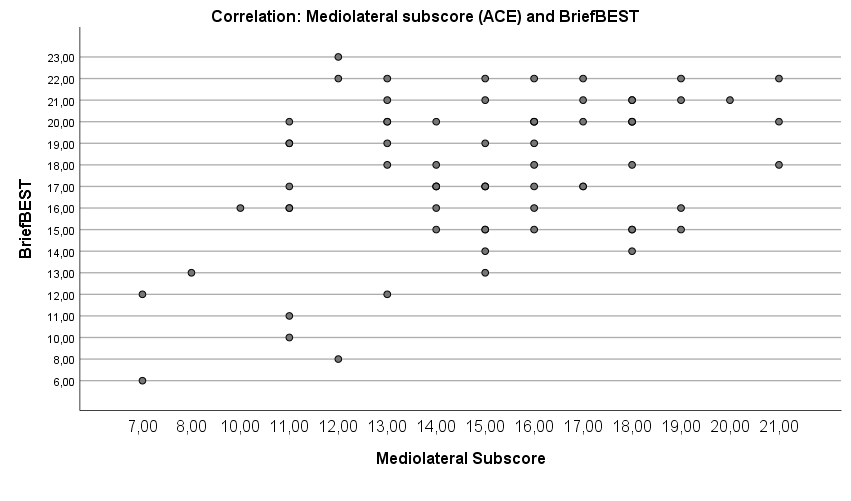


Scatter plot 45. ACE: All-step-count evaluation. BriefBEST: Brief Balance Evaluation Systems Test


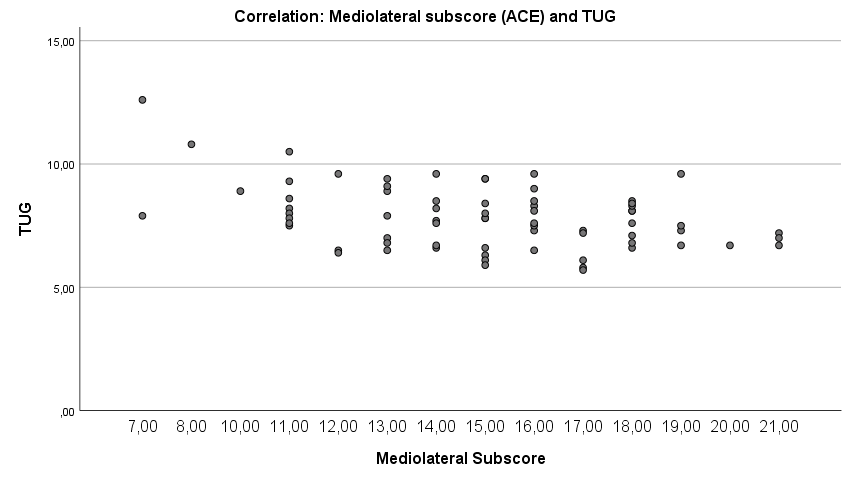


Scatter plot 46. STT: Stepping Threshold Test. ACE: All-step-count evaluation. TUG: Timed Up and Go


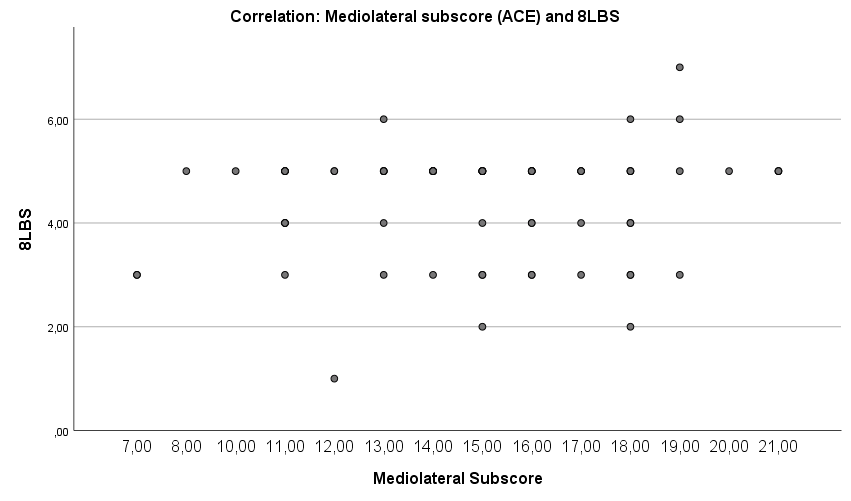


Scatter plot 47. ACE: All-step-count evaluation. 8LBS: 8-level balance scale


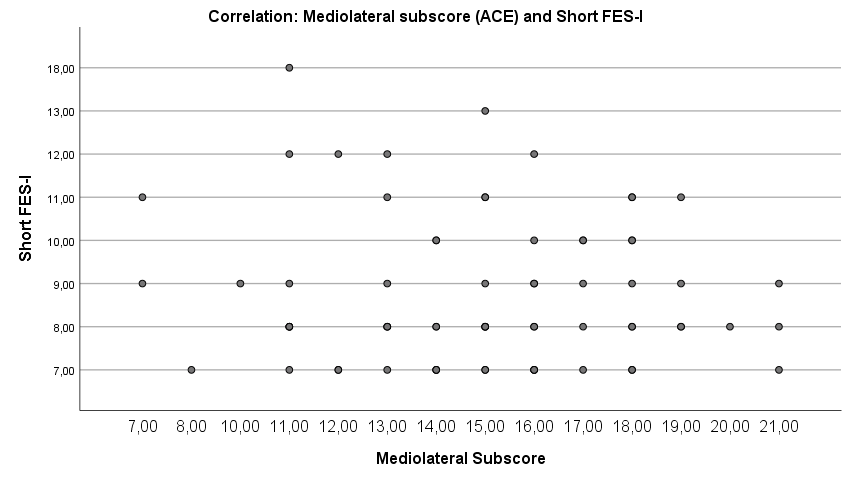


Scatter plot 48. ACE: All-step-count evaluation. Short FES-I: Short Falls Efficacy Scale – International


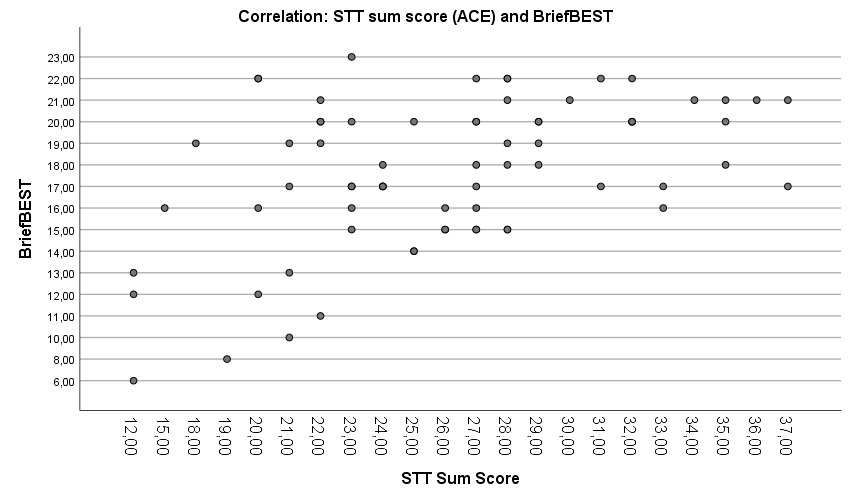


Scatter plot 49. STT: Stepping Threshold Test. ACE: All-step-count evaluation. BriefBEST: Brief Balance Evaluation Systems Test


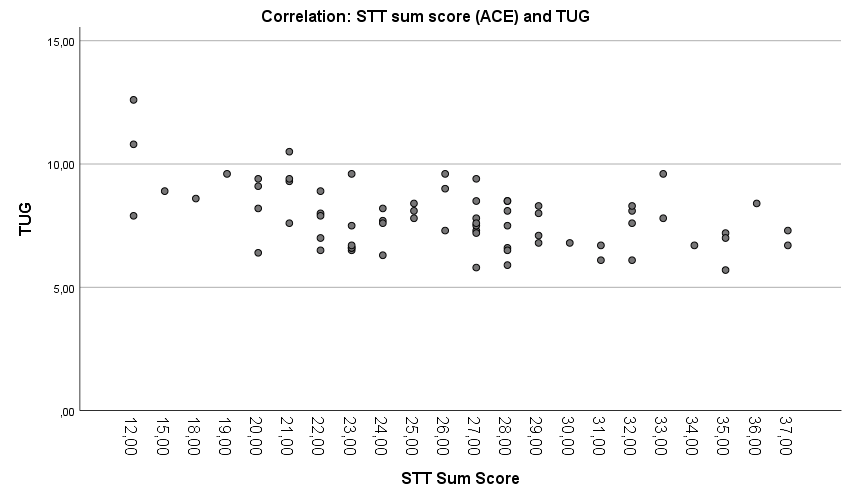


Scatter plot 50. STT: Stepping Threshold Test. ACE: All-step-count evaluation. TUG: Timed Up and Go


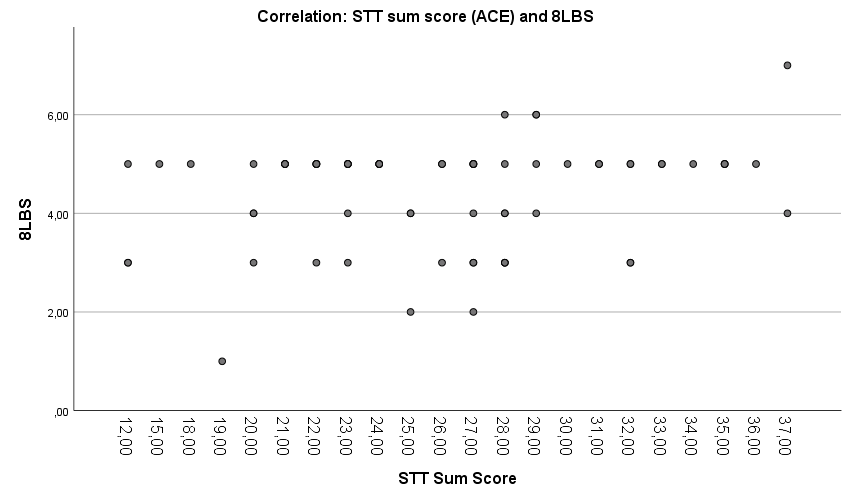


Scatter plot 51. STT: Stepping Threshold Test. ACE: All-step-count evaluation. 8LBS: 8-level balance scale


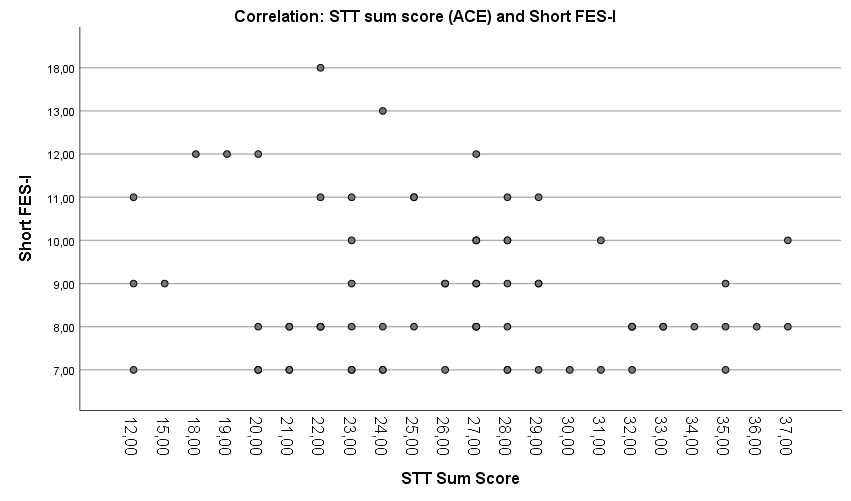


Scatter plot 52. STT: Stepping Threshold Test. ACE: All-step-count evaluation. Short FES-I: Short Falls Efficacy Scale – International

**Supplement 3.1 Scatterplots – Correlations between STT (ACE) and reference measures**


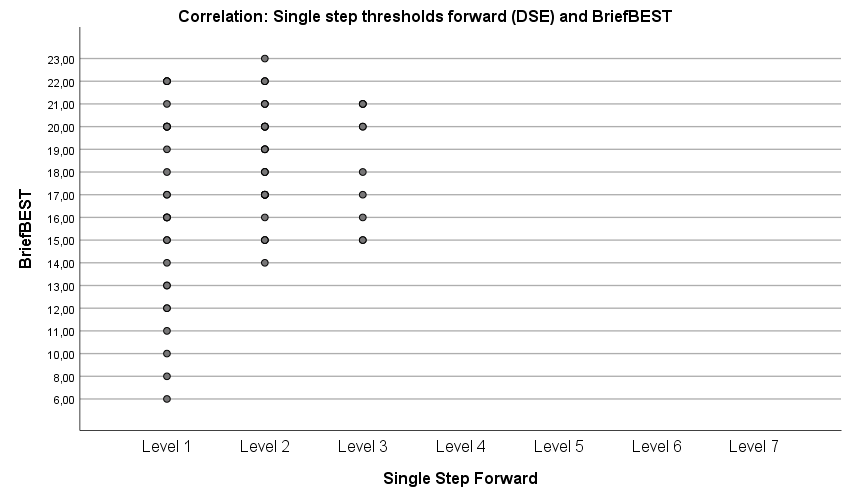


Scatter plot 53. DSE: Direction-Sensitive Evaluation. BriefBEST: Brief Balance Evaluation Systems Test.


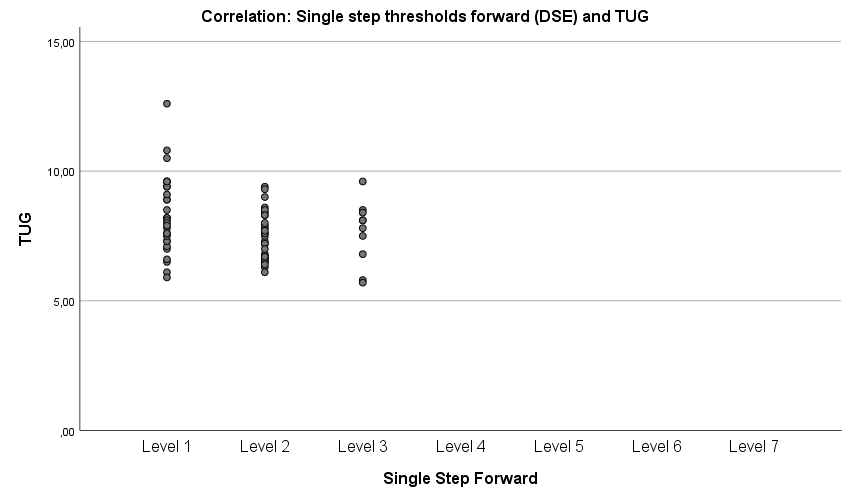


Scatter plot 54. DSE: Direction-Sensitive Evaluation. TUG: Timed Up and Go


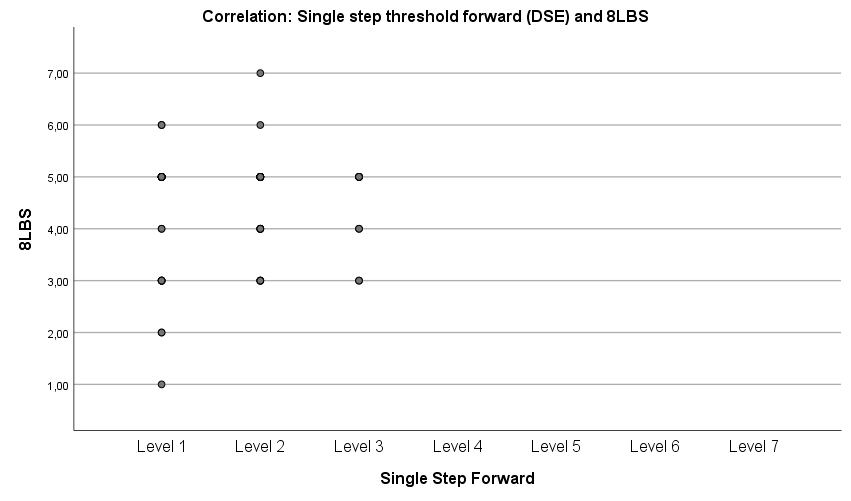


Scatter plot 55. DSE: Direction-Sensitive Evaluation. 8LBS: 8-level balance scale


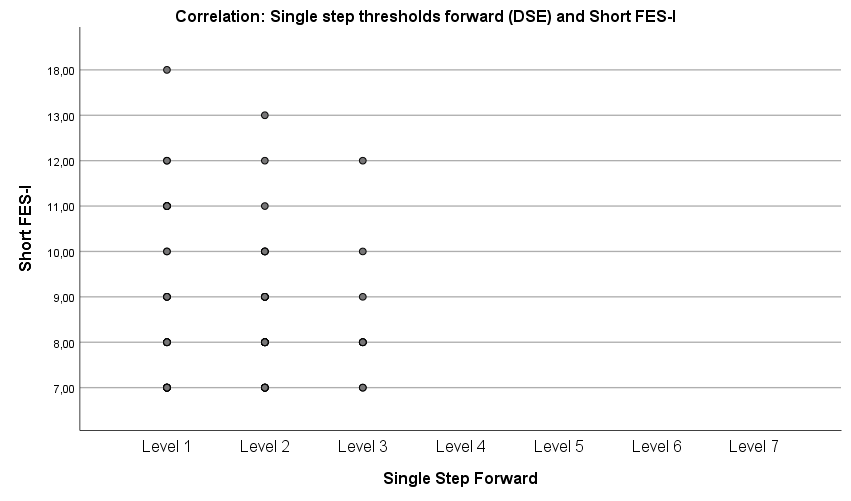


Scatter plot 56. DSE: Direction-Sensitive Evaluation. Short FES-I: Short Falls Efficacy Scale - International


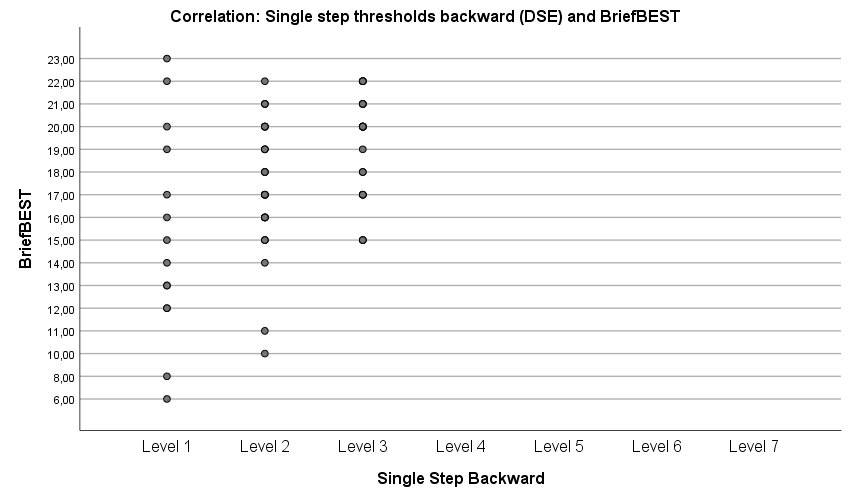


Scatter plot 57. DSE: Direction-Sensitive Evaluation. BriefBEST: Brief Balance Evaluation Systems Test.


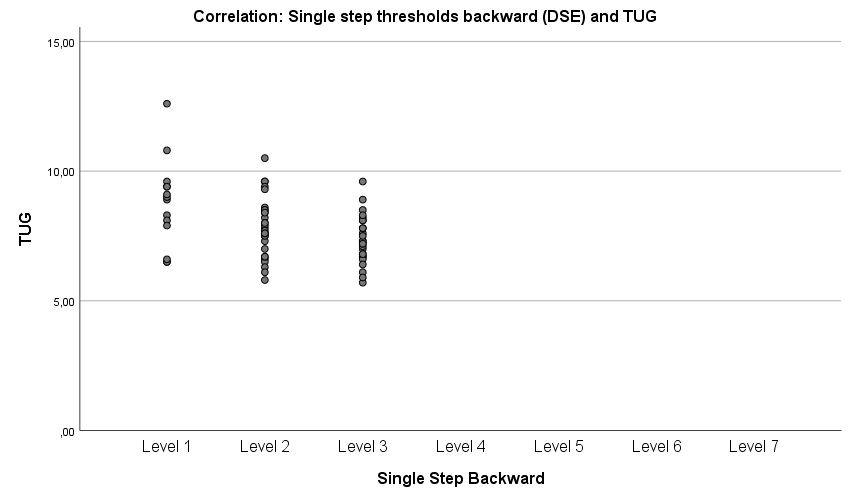


Scatter plot 58. DSE: Direction-Sensitive Evaluation. TUG: Timed Up and Go


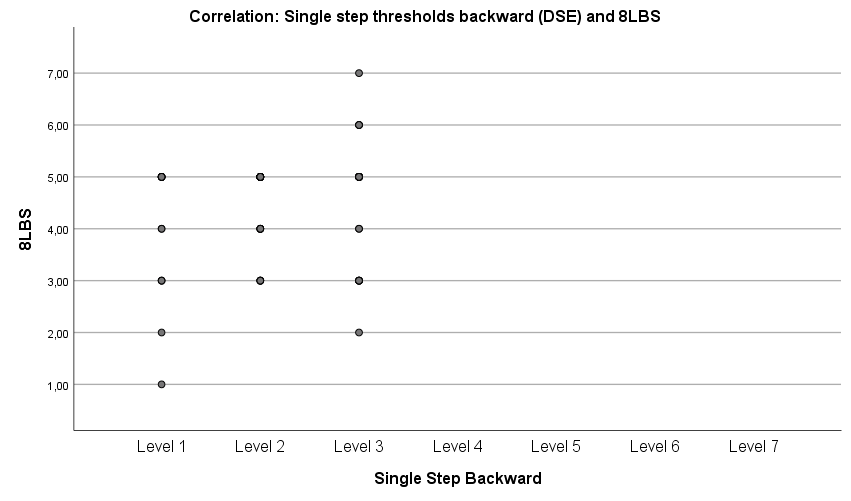


Scatter plot 59. DSE: Direction-Sensitive Evaluation. 8LBS: 8-level balance scale


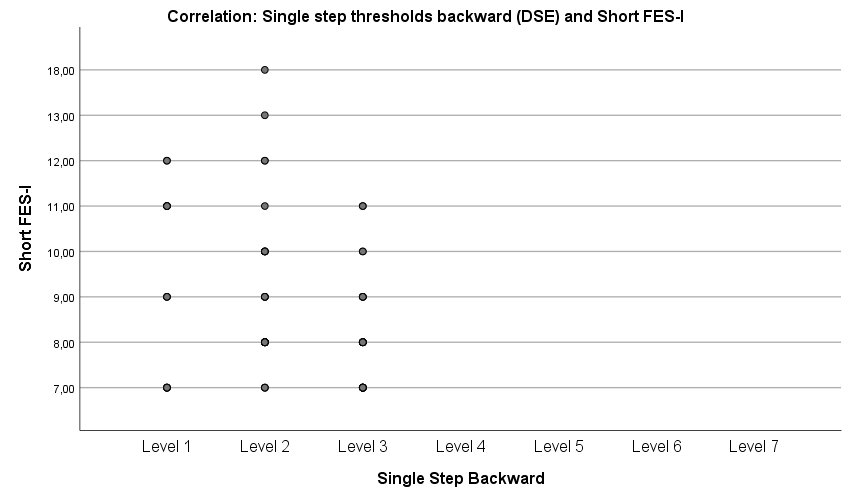


Scatter plot 60. DSE: Direction-Sensitive Evaluation. Short FES-I: Short Falls Efficacy Scale - International


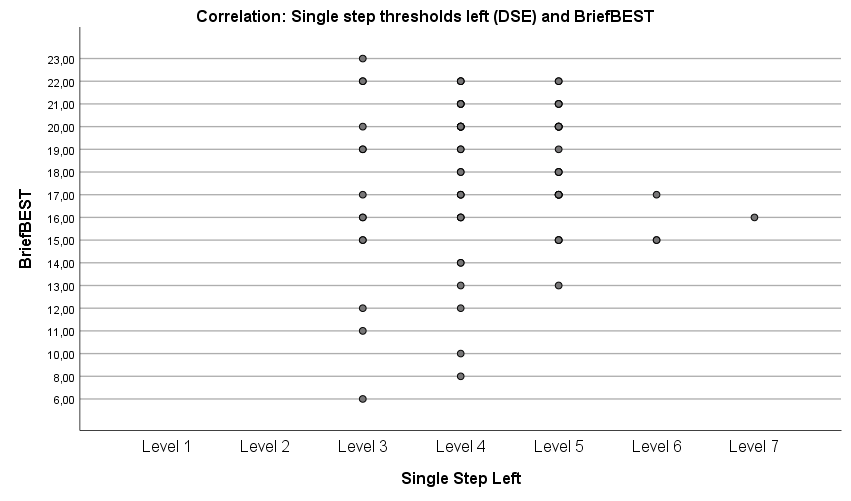


Scatter plot 61. DSE: Direction-Sensitive Evaluation. BriefBEST: Brief Balance Evaluation Systems Test.


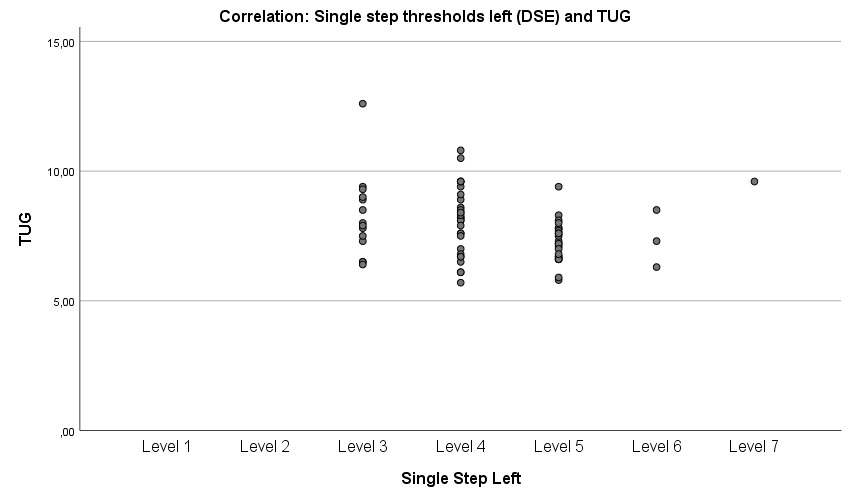


Scatter plot 62. DSE: Direction-Sensitive Evaluation. TUG: Timed Up and Go


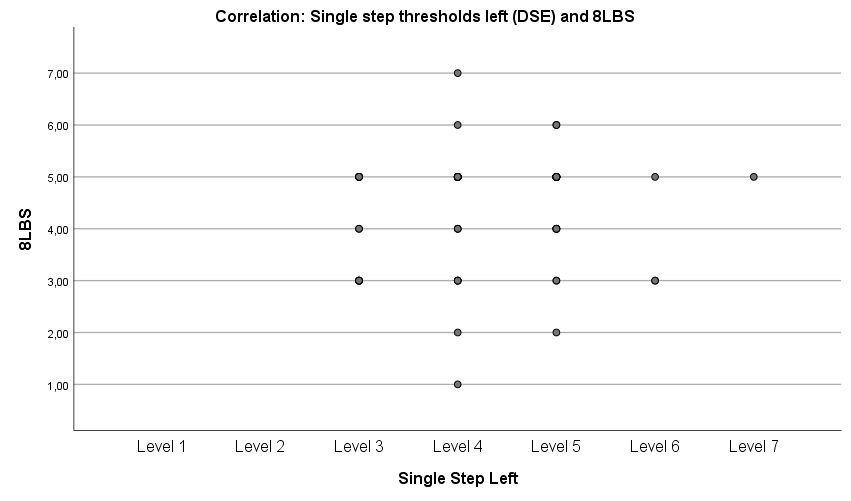


Scatter plot 63. DSE: Direction-Sensitive Evaluation. 8LBS: 8-level balance scale


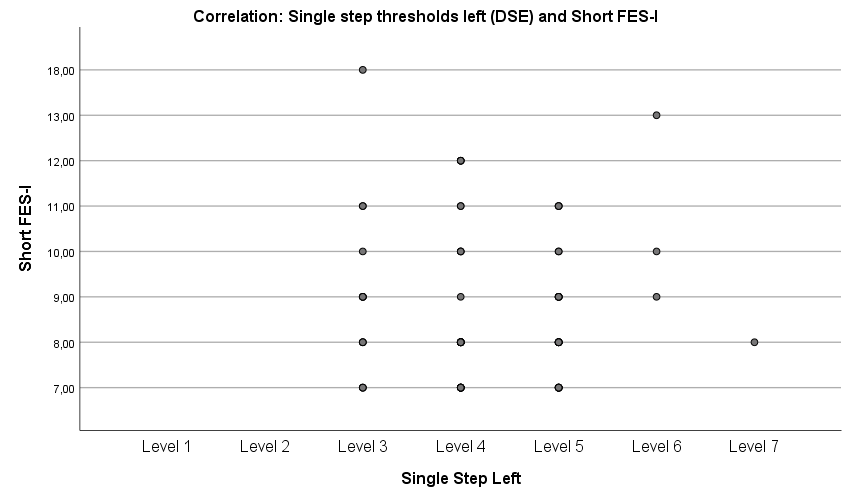


Scatter plot 64. DSE: Direction-Sensitive Evaluation. Short FES-I: Short Falls Efficacy Scale - International


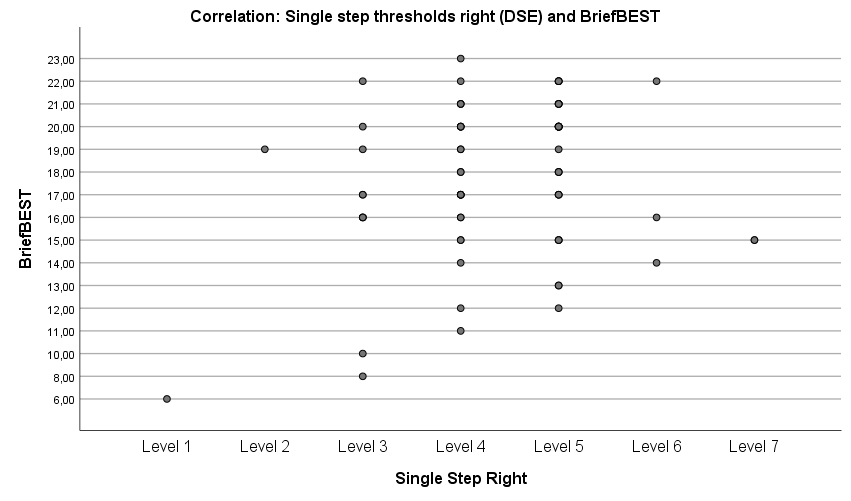


Scatter plot 65. DSE: Direction-Sensitive Evaluation. BriefBEST: Brief Balance Evaluation Systems Test.


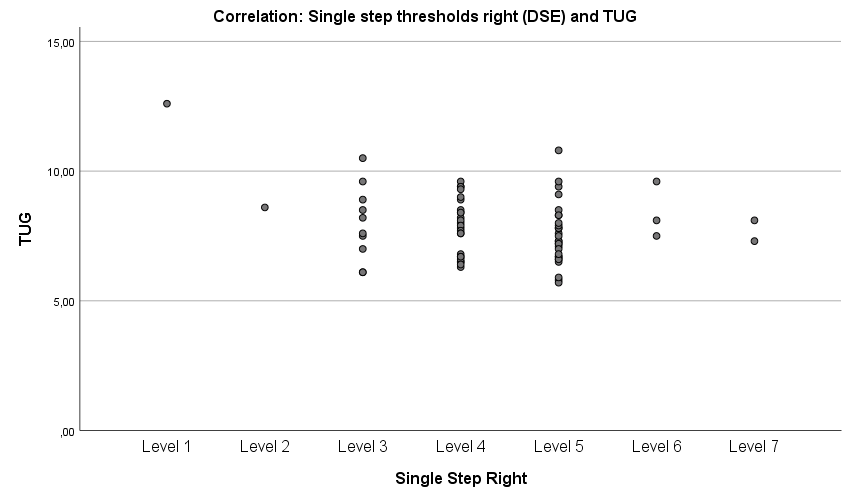


Scatter plot 66. DSE: Direction-Sensitive Evaluation. TUG: Timed Up and Go


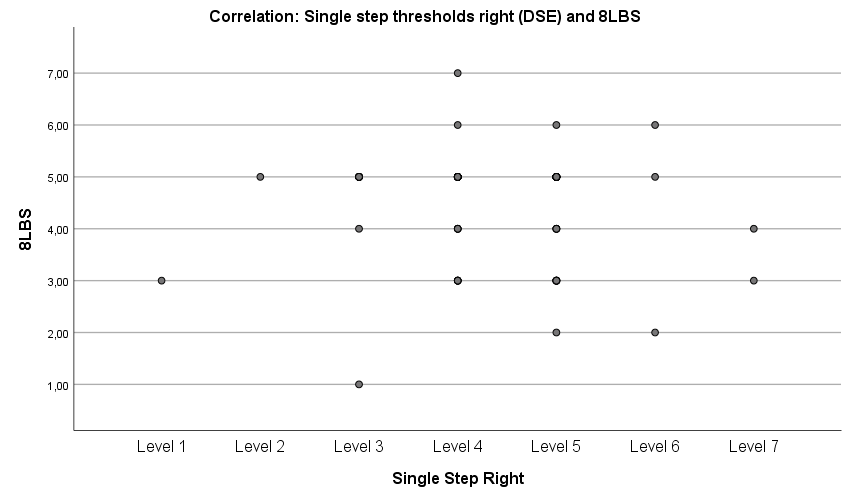


Scatter plot 67. DSE: Direction-Sensitive Evaluation. 8LBS: 8-level balance scale


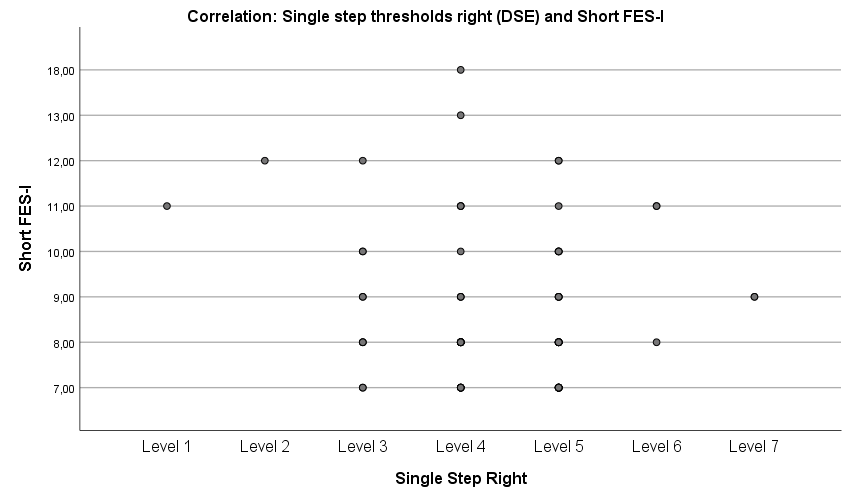


Scatter plot 68. DSE: Direction-Sensitive Evaluation. Short FES-I: Short Falls Efficacy Scale - International


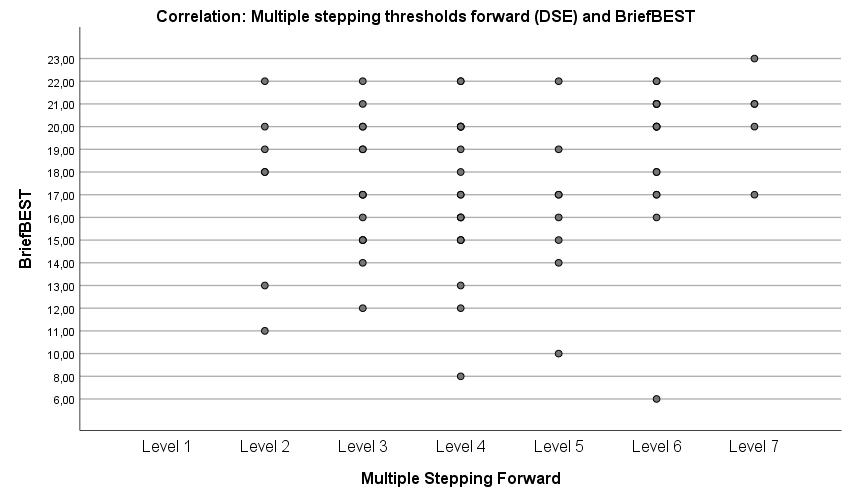


Scatter plot 69. DSE: Direction-Sensitive Evaluation. BriefBEST: Brief Balance Evaluation Systems Test


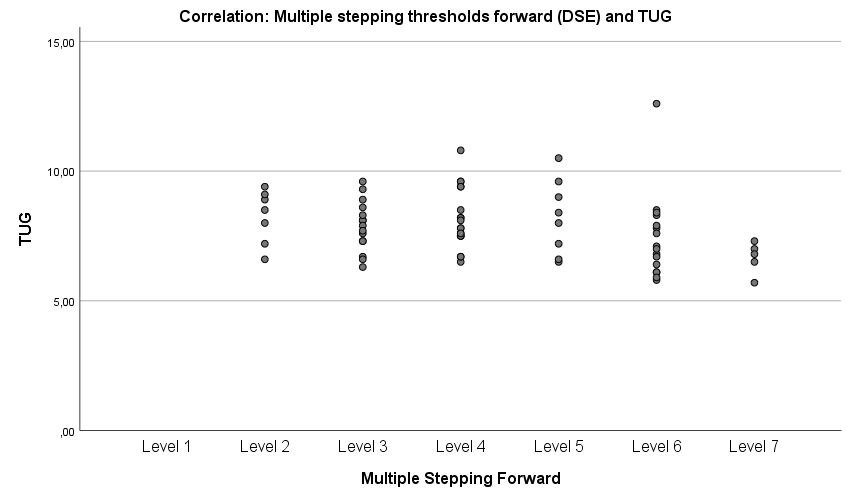


Scatter plot 70. DSE: Direction-Sensitive Evaluation. TUG: Timed Up and Go


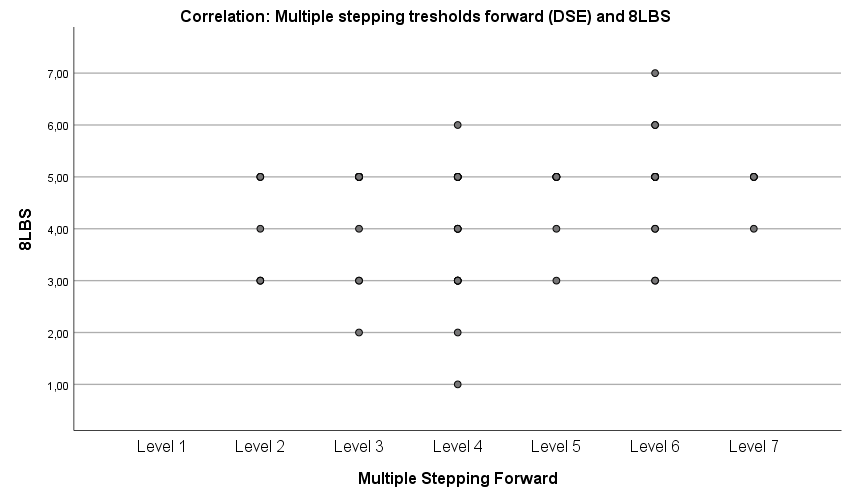


Scatter plot 71. DSE: Direction-Sensitive Evaluation. 8LBS: 8-level balance scale


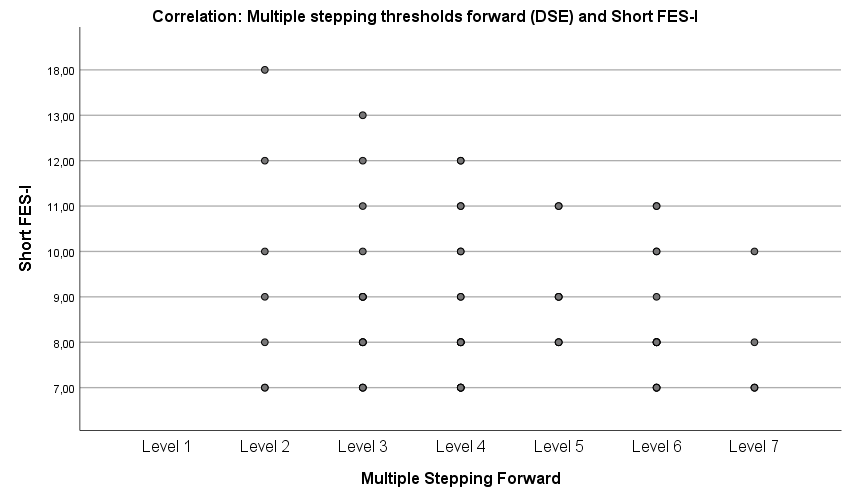


Scatter plot 72. DSE: Direction-Sensitive Evaluation. Short FES-I: Short Falls Efficacy Scale - International


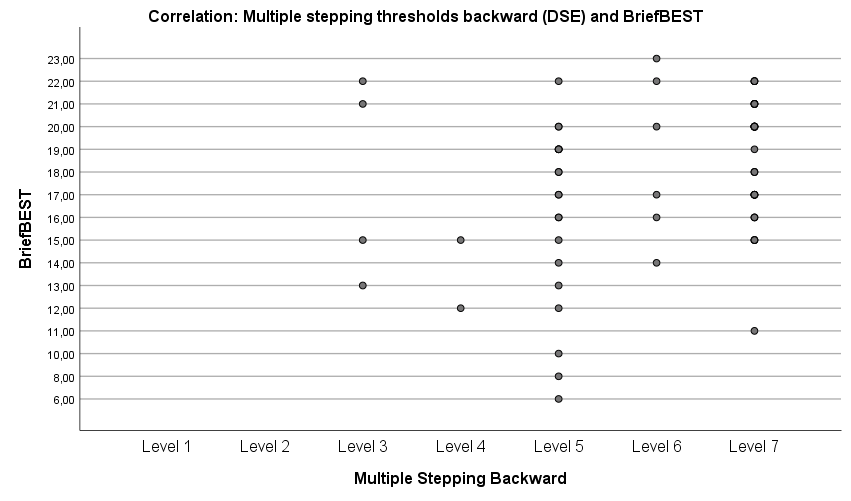


Scatter plot 73. DSE: Direction-Sensitive Evaluation. BriefBEST: Brief Balance Evaluation Systems Test


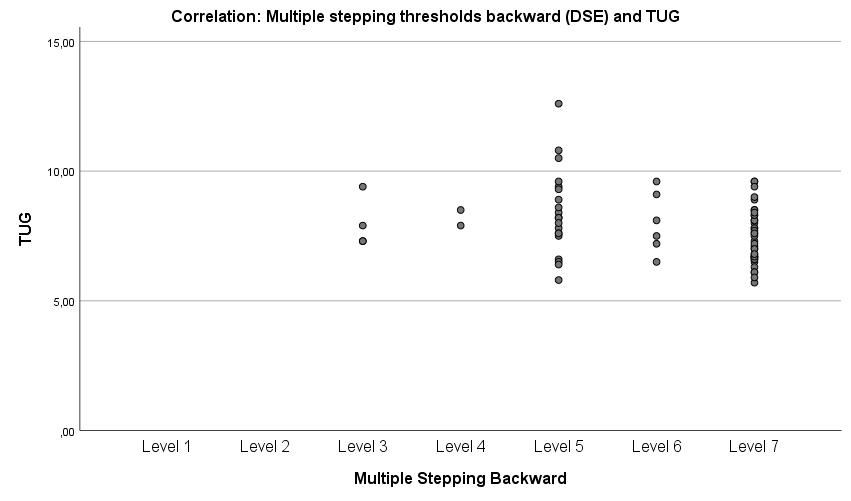


Scatter plot 74. DSE: Direction-Sensitive Evaluation. TUG: Timed Up and Go


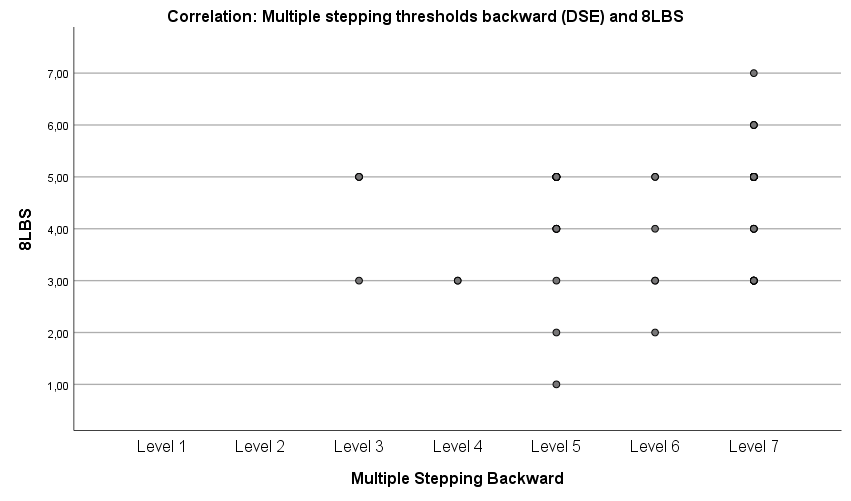


Scatter plot 75. DSE: Direction-Sensitive Evaluation. 8LBS: 8-level balance scale


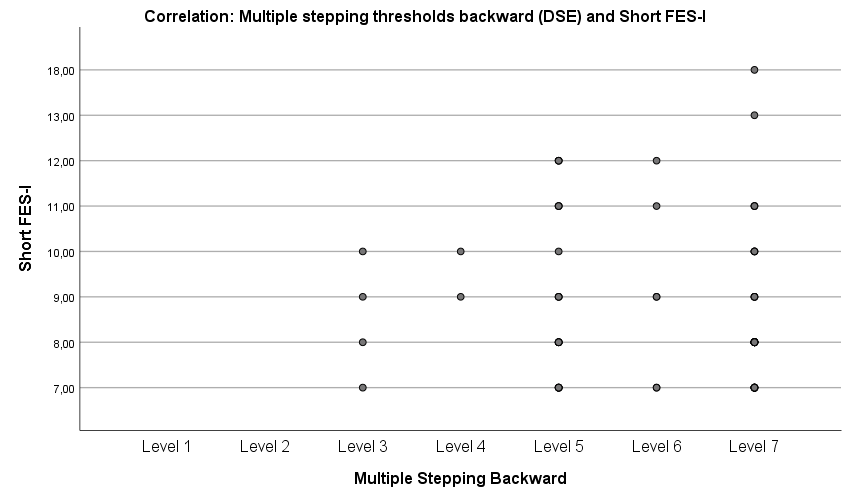


Scatter plot 76. DSE: Direction-Sensitive Evaluation. Short FES-I: Short Falls Efficacy Scale - International


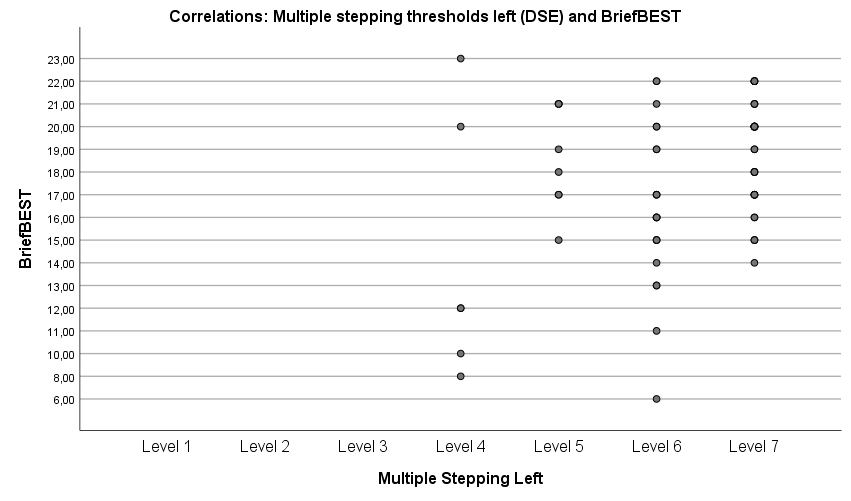


Scatter plot 77. DSE: Direction-Sensitive Evaluation. BriefBEST: Brief Balance Evaluation Systems Test


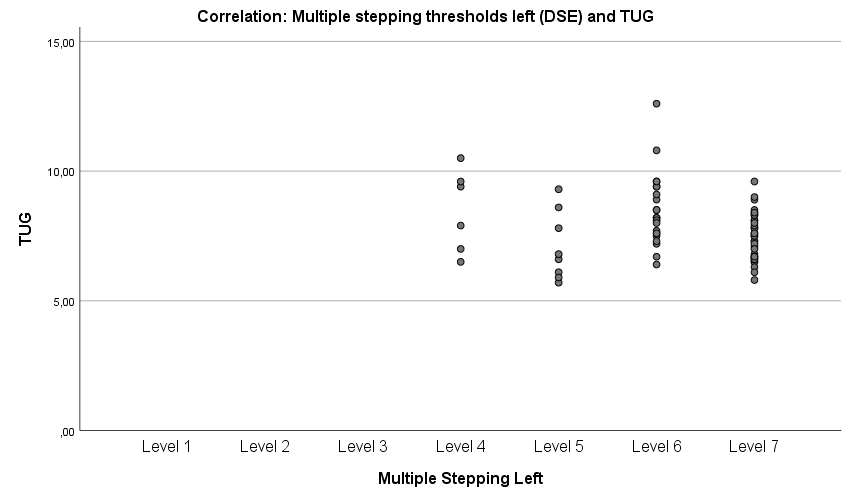


Scatter plot 78. DSE: Direction-Sensitive Evaluation. TUG: Timed Up and Go


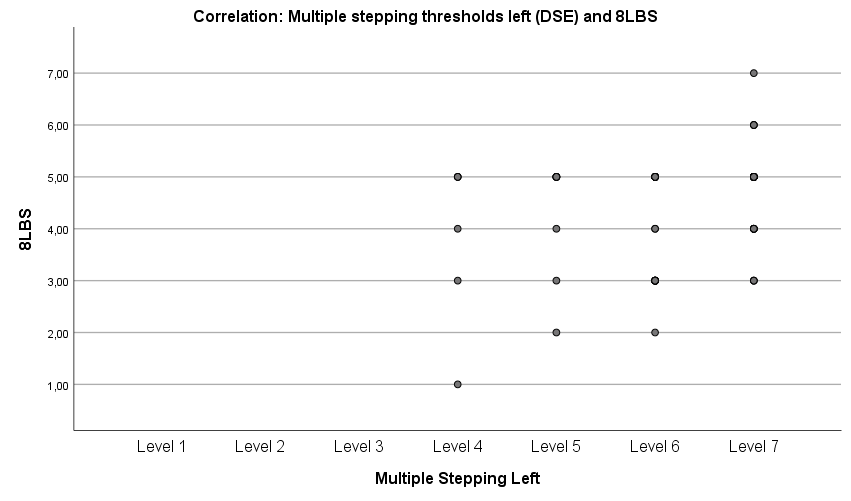


Scatter plot 79. DSE: Direction-Sensitive Evaluation. 8LBS: 8-level balance scale


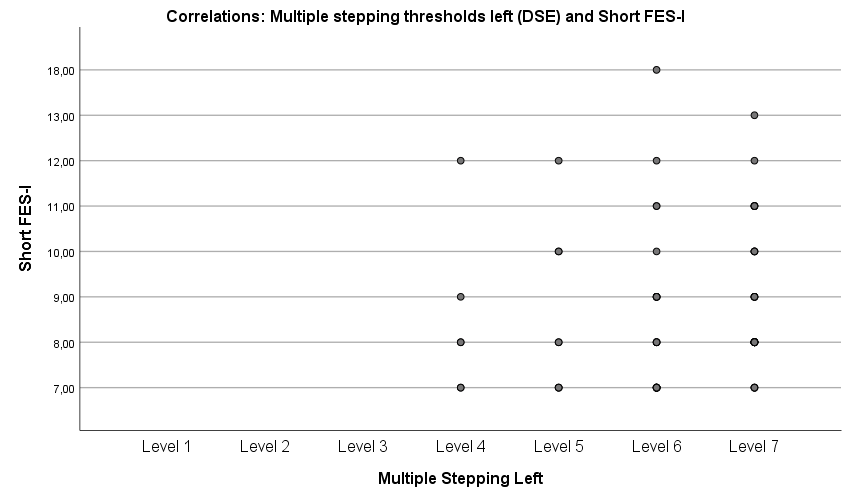


Scatter plot 80. DSE: Direction-Sensitive Evaluation. Short FES-I: Short Falls Efficacy Scale - International


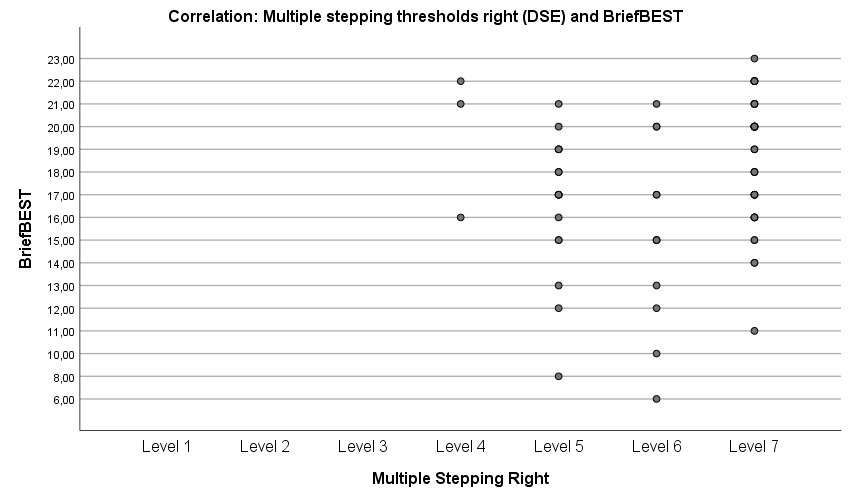


Scatter plot 81. DSE: Direction-Sensitive Evaluation. BriefBEST: Brief Balance Evaluation Systems Test


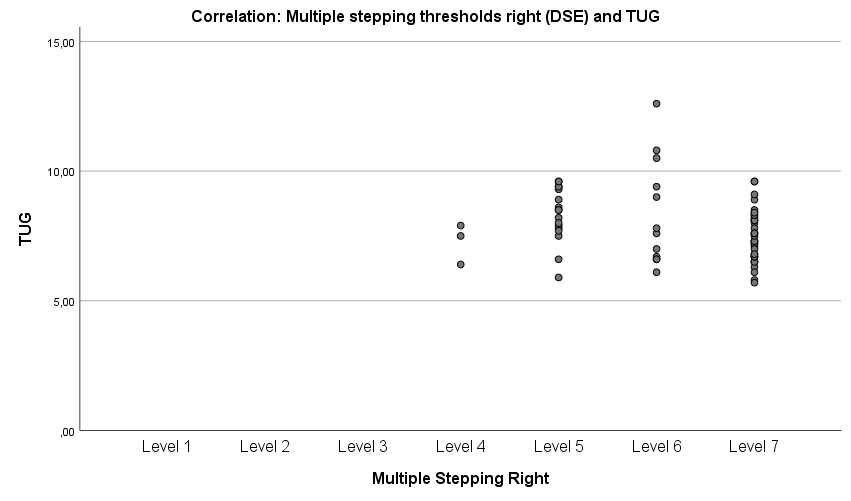


Scatter plot 82. DSE: Direction-Sensitive Evaluation. TUG: Timed Up and Go


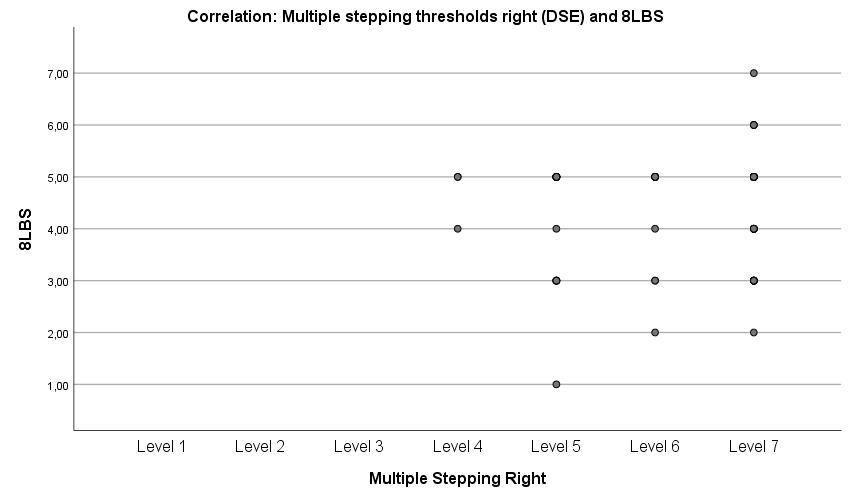


Scatter plot 83. DSE: Direction-Sensitive Evaluation. 8LBS: 8-level balance scale


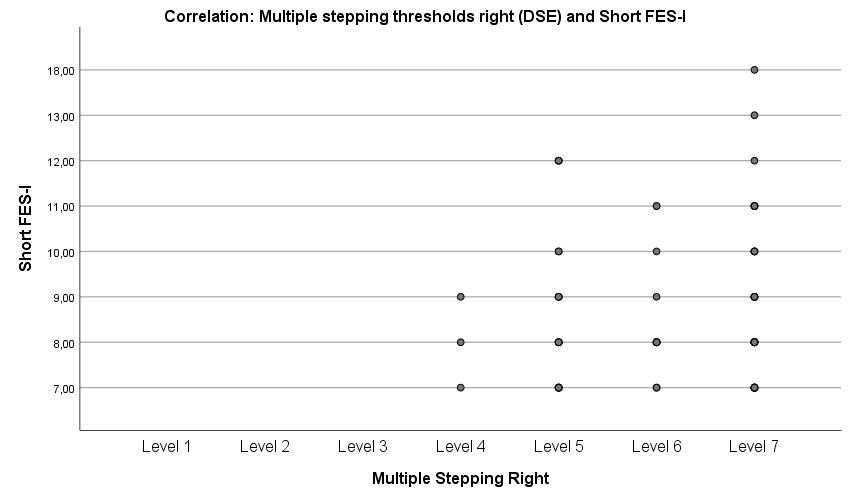


Scatter plot 84. DSE: Direction-Sensitive Evaluation. Short FES-I: Short Falls Efficacy Scale - International


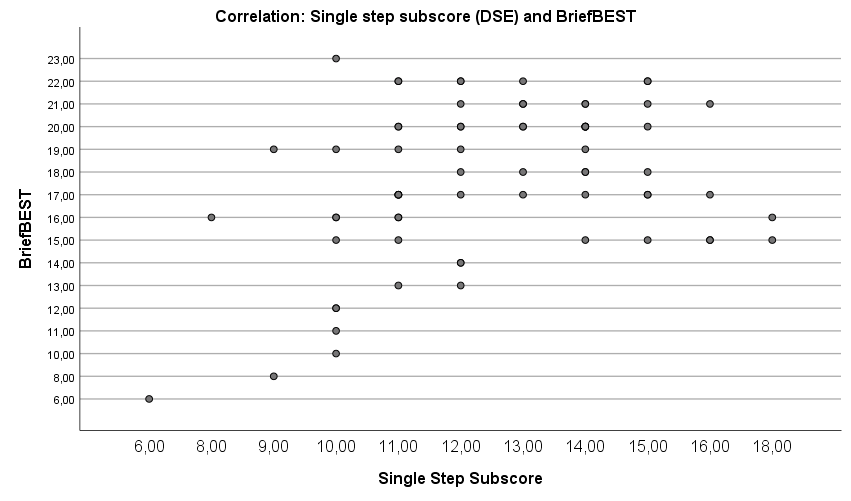


Scatter plot 85. DSE: Direction-Sensitive Evaluation. BriefBEST: Brief Balance Evaluation Systems Test


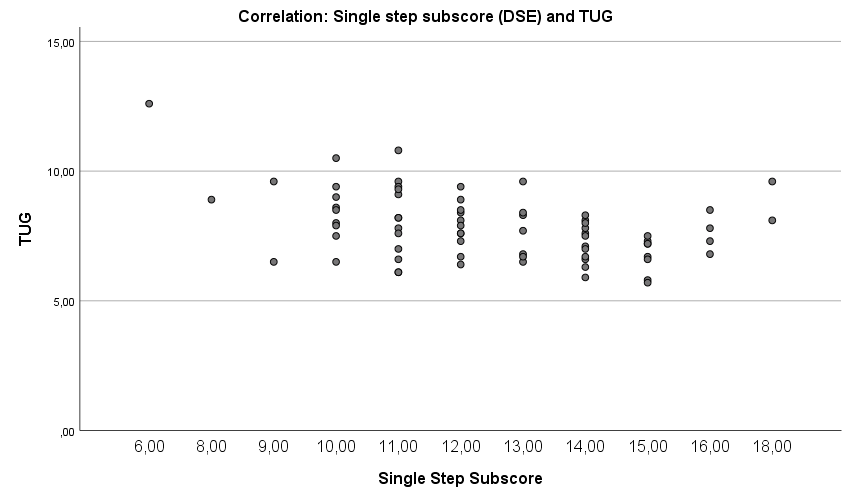


Scatter plot 86. DSE: Direction-Sensitive Evaluation. TUG: Timed Up and Go


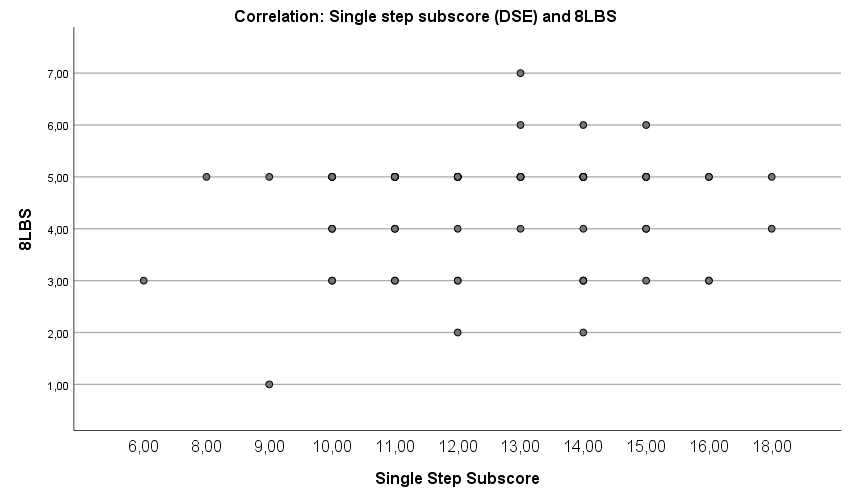


Scatter plot 87. DSE: Direction-Sensitive Evaluation. 8LBS: 8-level balance scale


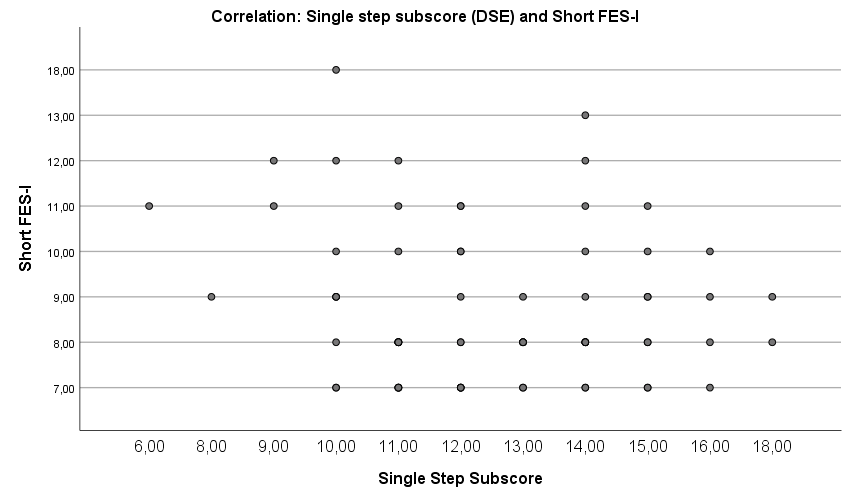


Scatter plot 88. DSE: Direction-Sensitive Evaluation. Short FES-I: Short Falls Efficacy Scale - International


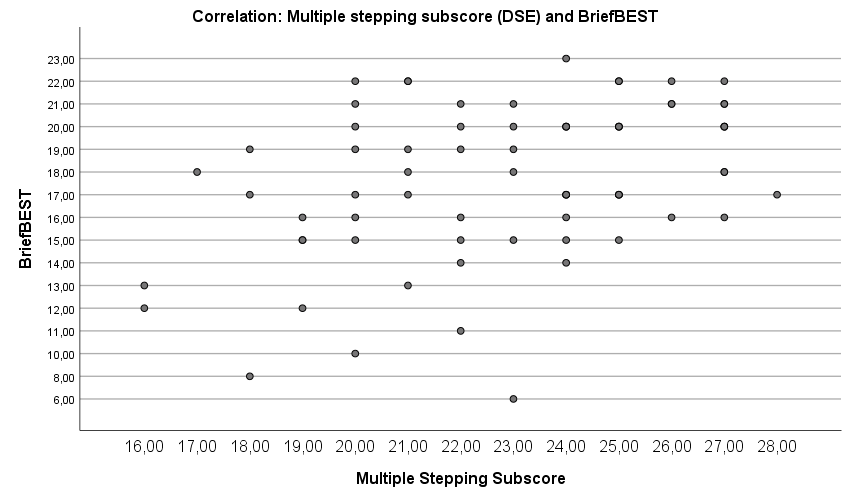


Scatter plot 89. DSE: Direction-Sensitive Evaluation. BriefBEST: Brief Balance Evaluation Systems Test


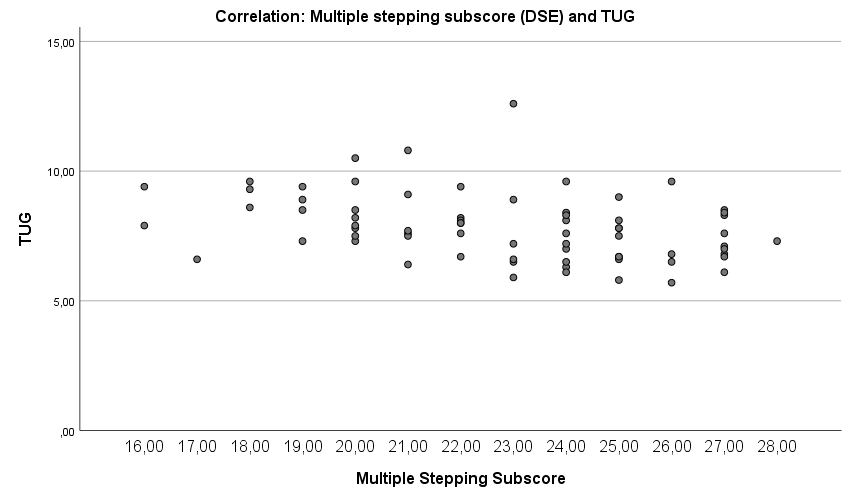


Scatter plot 90. DSE: Direction-Sensitive Evaluation. TUG: Timed Up and Go


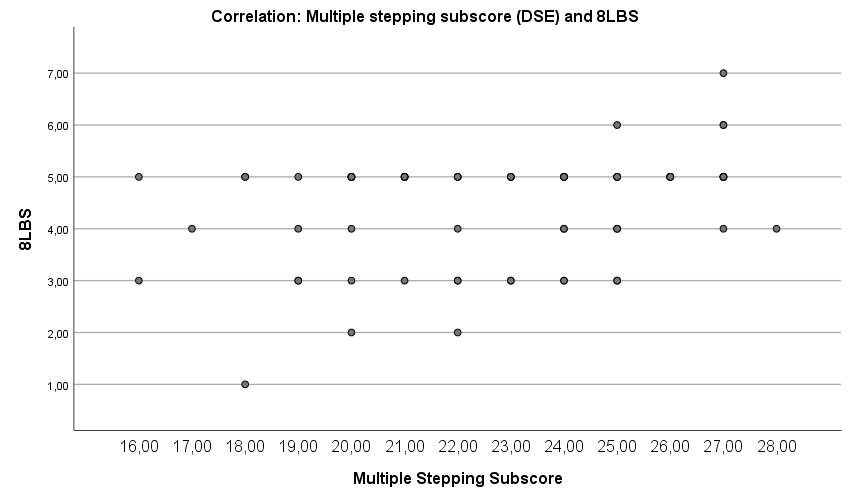


Scatter plot 91. DSE: Direction-Sensitive Evaluation. 8LBS: 8-level balance scale


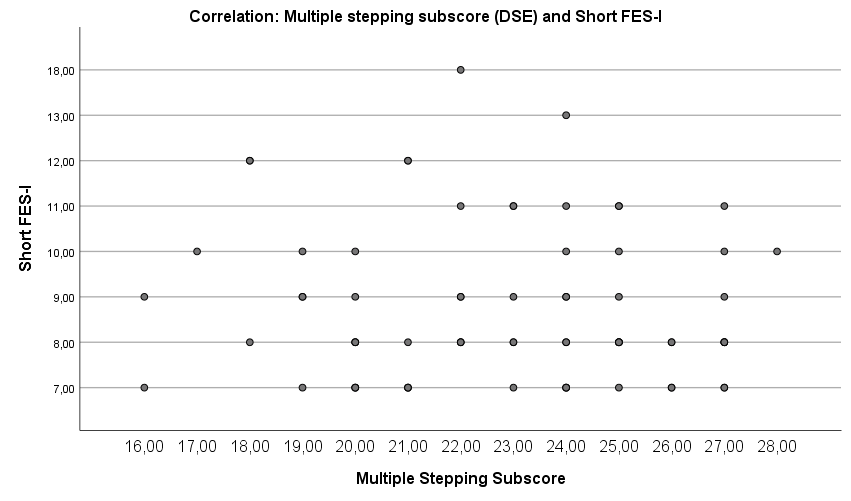


Scatter plot 92. DSE: Direction-Sensitive Evaluation. Short FES-I: Short Falls Efficacy Scale - International


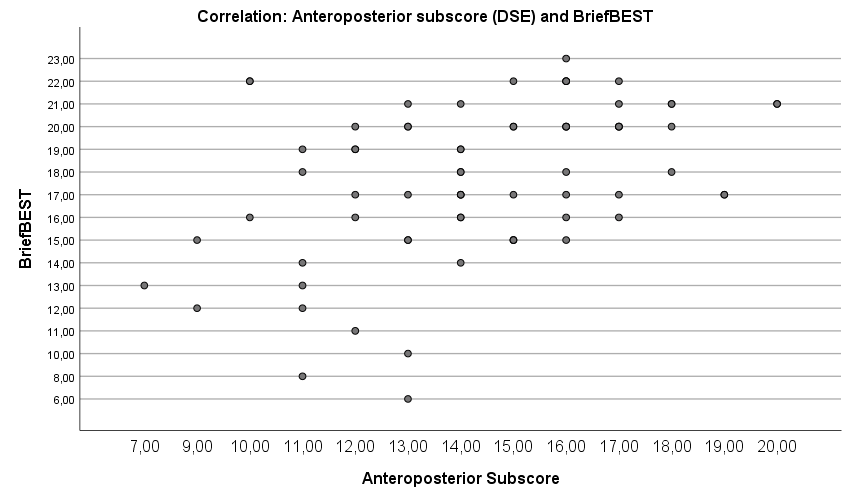


Scatter plot 93. DSE: Direction-Sensitive Evaluation. BriefBEST: Brief Balance Evaluation Systems Test


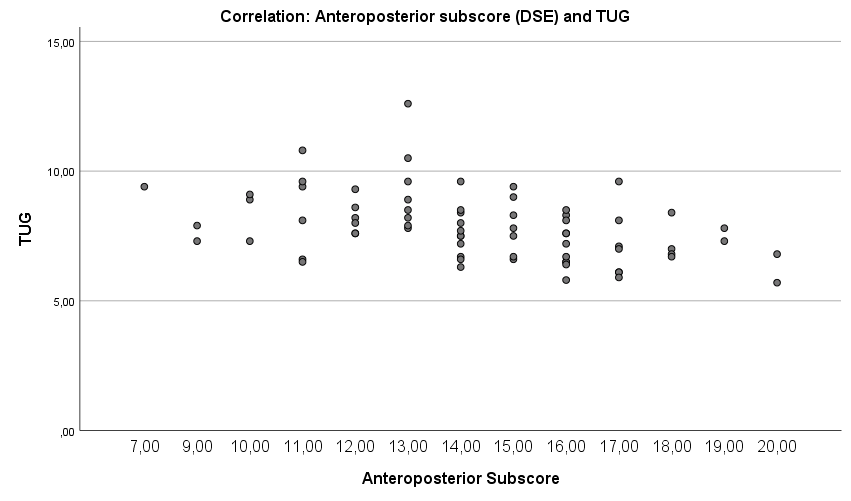


Scatter plot 94. DSE: Direction-Sensitive Evaluation. TUG: Timed Up and Go


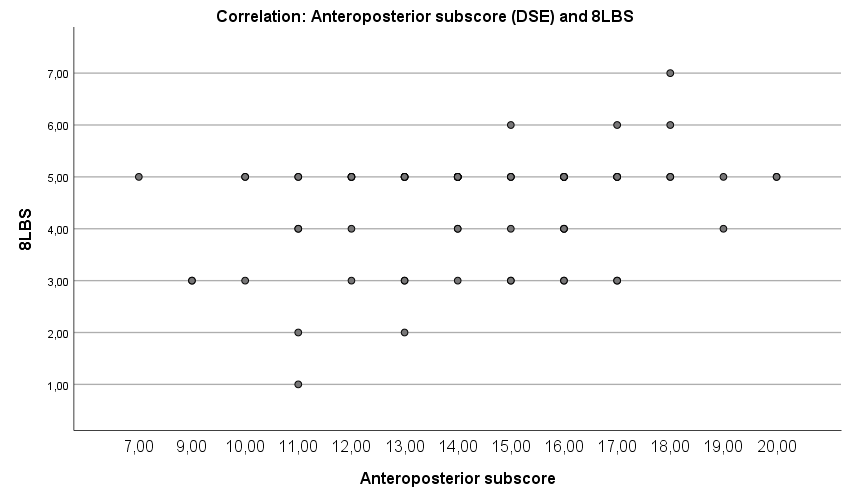


Scatter plot 95. DSE: Direction-Sensitive Evaluation. 8LBS: 8-level balance scale


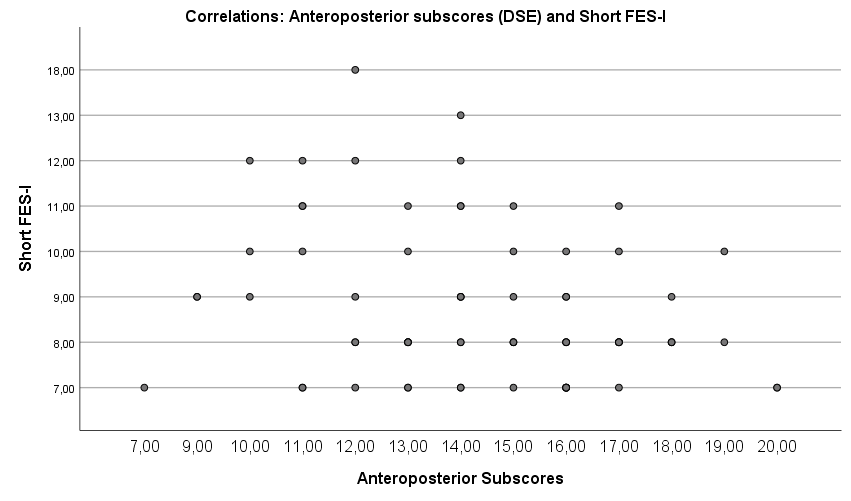


Scatter plot 96. DSE: Direction-Sensitive Evaluation. Short FES-I: Short Falls Efficacy Scale - International


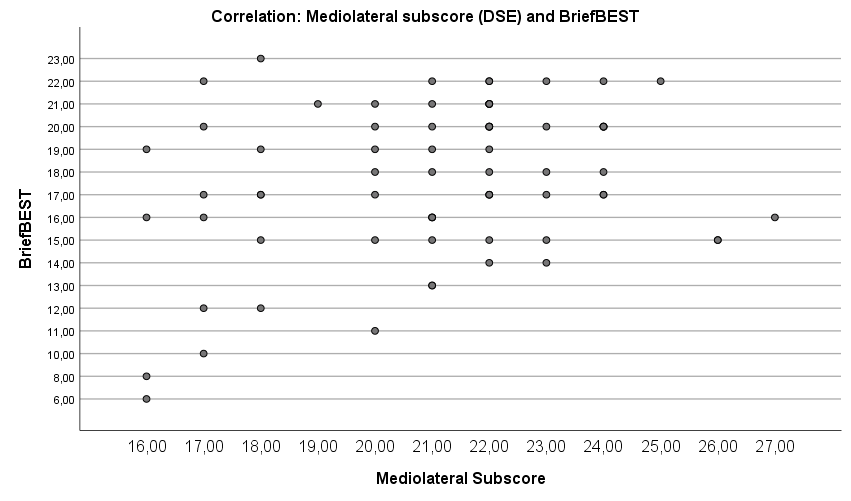


Scatter plot 97. DSE: Direction-Sensitive Evaluation. BriefBEST: Brief Balance Evaluation Systems Test


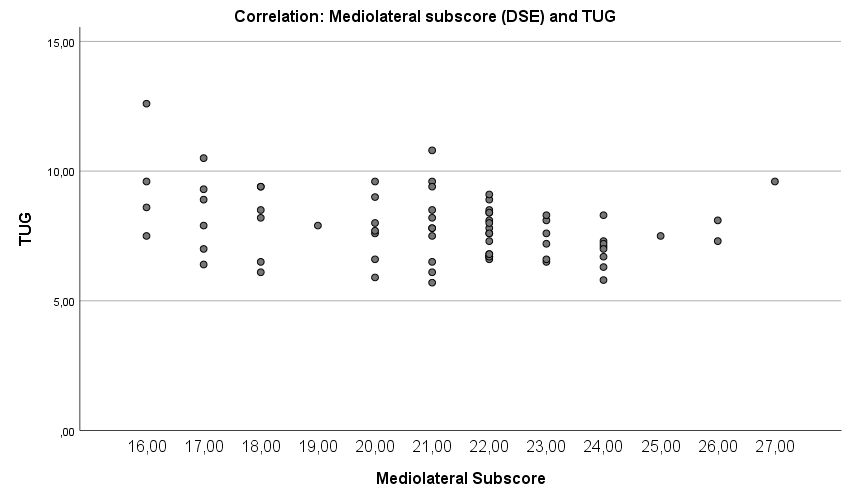


Scatter plot 98. DSE: Direction-Sensitive Evaluation. TUG: Timed Up and Go


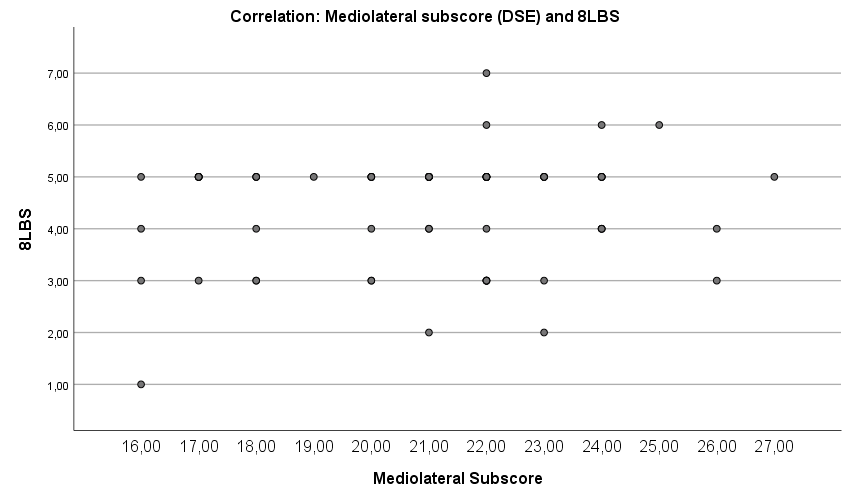


Scatter plot 99. DSE: Direction-Sensitive Evaluation. 8LBS: 8-level balance scale


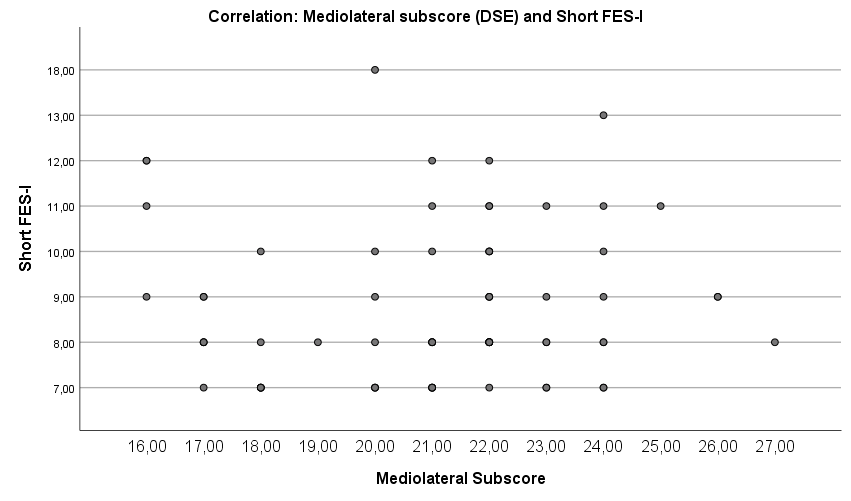


Scatter plot 100. DSE: Direction-Sensitive Evaluation. Short FES-I: Short Falls Efficacy Scale - International

Scatter plot 101. STT: Stepping Threshold Test. DSE: Direction-Sensitive Evaluation. BriefBEST: Brief Balance Evaluation Systems Test

Scatter plot 102. STT: Stepping Threshold Test. DSE: Direction-Sensitive Evaluation. TUG: Timed Up and Go

Scatter plot 103. STT: Stepping Threshold Test. DSE: Direction-Sensitive Evaluation. 8LBS: 8-level balance scale

Scatter plot 104. STT: Stepping Threshold Test. DSE: Direction-Sensitive Evaluation. Short FES-I: Short Falls Efficacy Scale - International
