# Supplementary material for: The Stepping Threshold Test for Reactive Balance: Validation of Two Observer-Based Evaluation Strategies to Assess Stepping Behavior in Fall-Prone Older Adults
Source: Front Sports Act Living. 2021 Oct 11;3:715392. doi: 10.3389/fspor.2021.715392 (PMC8542787; doi:10.3389/fspor.2021.715392)
Supplement: Supplementary file 1 [file Data_Sheet_1.zip › Supplement 4.1, 4.2.DOCX]

Supplementary Material

Supplement 4.1 Correlation between STT (DSE) and reference measures

|  | **STT-Thresholds** |  | **Brief BEST** | **TUG** | **8LBS** | **Short FES-I** |
| --- | --- | --- | --- | --- | --- | --- |
| Antero-posterior | Single Step Forward | r | 0.222 | -0.232 | 0.040 | -0.106 |
|  |  | CI95 | -0.02 - 0.44 | -0.45 - 0.01 | -0.2 - 0.27 | -0.33 - 0.13 |
|  | Single Step Backward | r | 0.415 | -0.342 | 0.203 | -0.241* |
|  |  | CI95 | 0.19 - 0.6 | -0.54 - -0.11 | -0.04 - 0.42 | -0.45 - 0 |
| Medio-lateral | Single Step Left | r | 0.027 | -0.238 | 0.082 | <0.001 |
|  |  | CI95 | -0.21 - 0.26 | -0.45 - 0 | -0.16 - 0.31 | -0.24 - 0.23 |
|  | Single Step Right | r | 0.135 | -0.156 | -0.105 | -0.052 |
|  |  | CI95 | -0.1 - 0.36 | -0.38 - 0.08 | -0.33 - 0.13 | -0.28 - 0.19 |
| Antero-posterior | Multiple Stepping Forward | r | 0.263 | -0.318 | 0.228 | -0.174 |
|  |  | CI95 | 0.03 - 0.47 | -0.52 - -0.08 | -0.01 - 0.44 | -0.39 - 0.07 |
|  | Multiple Stepping Backward | r | 0.287 | -0.267 | 0.205 | -0.128 |
|  |  | CI95 | 0.05 - 0.49 | -0.48 - -0.03 | -0.03 - 0.42 | -0.35 - 0.11 |
| Medio-lateral | Multiple Stepping Left | r | 0.262 | -0.173 | 0.289 | 0.157 |
|  |  | CI95 | 0.02 - 0.47 | -0.39 - 0.07 | 0.05 - 0.49 | -0.08 - 0.38 |
|  | Multiple Stepping Right | r | 0.261 | -0.238 | 0.106 | 0.066 |
|  |  | CI95 | 0.02 - 0.47 | -0.45 - 0 | -0.13 - 0.33 | -0.17 - 0.3 |

CI95: confidence interval of 95%; r: correlation coefficient rho, calculated by means of the Spearman-rank-correlation; TUG: Timed Up and Go Test; Brief BEST: Brief Balance Evaluations Systems Test; 8LBS: Eight level balance scale; FES-I: Short Falls Efficacy Scale – International

Supplement 4.2 Differences between non-fallers and fallers in the STT (DSE)

|  | Non-fallers (n=38) | | | | |  | Fallers (n=32) | | | | |  |  |
| --- | --- | --- | --- | --- | --- | --- | --- | --- | --- | --- | --- | --- | --- |
|  | Mean | Median | IQR | Min. | Max. |  | Mean | Median | IQR | Min. | Max. |  | Sign. |
| Single Step Forward | 1.82 | 2.0 | 1.25 | 1 | 3 |  | 1.63 | 2.0 | 1.0 | 1 | 3 |  | 0.399 |
| Single Step Backward | 2.05 | 2.0 | 2.0 | 1 | 3 |  | 2.31 | 2.0 | 1.0 | 1 | 3 |  | 0.155 |
| Single Step Left | 4.26 | 4.0 | 1.0 | 3 | 7 |  | 4.25 | 4.0 | 1.0 | 3 | 6 |  | 0.990 |
| Single Step Right | 4.55 | 5.0 | 1.0 | 1 | 7 |  | 4.13 | 4.0 | 2.0 | 2 | 7 |  | 0.029* |
| Multiple Stepping Forward | 4.55 | 4.5 | 3.0 | 2 | 7 |  | 4.16 | 4.0 | 2.75 | 2 | 7 |  | 0.240 |
| Multiple Stepping Backward | 5.87 | 6.5 | 2.0 | 3 | 7 |  | 6.22 | 7.0 | 2.0 | 3 | 7 |  | 0.285 |
| Multiple Stepping Left | 6.32 | 7.0 | 1.0 | 4 | 7 |  | 6.06 | 6.0 | 1.75 | 4 | 7 |  | 0.332 |
| Multiple Stepping Right | 6.08 | 6.0 | 2.0 | 4 | 7 |  | 6.38 | 7.0 | 1.0 | 4 | 7 |  | 0.191 |

Sign.: Two-tailed significance calculated by means of the Mann-Whitney-U test. Significance level was set to p < 0.05. ; * p < 0.05
