# Supplementary material for: The Stepping Threshold Test for Reactive Balance: Validation of Two Observer-Based Evaluation Strategies to Assess Stepping Behavior in Fall-Prone Older Adults
Source: Front Sports Act Living. 2021 Oct 11;3:715392. doi: 10.3389/fspor.2021.715392 (PMC8542787; doi:10.3389/fspor.2021.715392)
Supplement: Supplementary file 1 [file Data_Sheet_1.zip › Supplement 5.1, 5.2.DOCX]

Supplementary Material

Supplement 5.1 Floor and ceiling effects of the STT sum scores (ACE)

|  | **Floor effect^a^** | **Ceiling effect^b^** |
| --- | --- | --- |
| STT sum score | 0.00% | 0.00% |
| SS subscore | 4.29% | 0.00% |
| MS subscore | 0.00% | 0.00% |
| AP subscore | 0.00% | 0.00% |
| ML subscore | 0.00% | 0.00% |

Floor or ceiling effect exist if the value is above 15 %. a Percentage of participants who reached the lowest level in the single or multiple stepping thresholds. b Percentage of participants who reached the highest single or multiple stepping thresholds. SS: Single step; MS: Multiple Stepping AP: Anteroposterior; ML: Mediolateral

Supplement 5.2 Floor and ceiling effects of the STT sum scores (DSE)

|  | **Floor effect^a^** | **Ceiling effect^b^** |
| --- | --- | --- |
| STT sum score | 0.00% | 0.00% |
| SS subscore | 0.00% | 0.00% |
| MS subscore | 0.00% | 1.43% |
| AP subscore | 0.00% | 0.00% |
| ML subscore | 0.00% | 0.00% |

Floor or ceiling effect exist if the value is above 15 %. a Percentage of participants who reached the lowest level in the single or multiple stepping thresholds. b Percentage of participants who reached the highest single or multiple stepping thresholds. SS: Single step; MS: Multiple Stepping AP: Anteroposterior; ML: Mediolateral
